# Diploid and tetraploid genomes of *Acorus* and the evolution of monocots

**DOI:** 10.1038/s41467-023-38829-3

**Published:** 2023-06-20

**Authors:** Liang Ma, Ke-Wei Liu, Zhen Li, Yu-Yun Hsiao, Yiying Qi, Tao Fu, Guang-Da Tang, Diyang Zhang, Wei-Hong Sun, Ding-Kun Liu, Yuanyuan Li, Gui-Zhen Chen, Xue-Die Liu, Xing-Yu Liao, Yu-Ting Jiang, Xia Yu, Yang Hao, Jie Huang, Xue-Wei Zhao, Shijie Ke, You-Yi Chen, Wan-Lin Wu, Jui-Ling Hsu, Yu-Fu Lin, Ming-Der Huang, Chia-Ying Li, Laiqiang Huang, Zhi-Wen Wang, Xiang Zhao, Wen-Ying Zhong, Dong-Hui Peng, Sagheer Ahmad, Siren Lan, Ji-Sen Zhang, Wen-Chieh Tsai, Yves Van de Peer, Zhong-Jian Liu

**Affiliations:** 1 Key Laboratory of National Forestry and Grassland Administration for Orchid Conservation and Utilization, Fujian Agriculture and Forestry University, Fuzhou, 350002 China; 2 Tsinghua-Berkeley Shenzhen Institute (TBSI), Center for Biotechnology and Biomedicine, Shenzhen Key Laboratory of Gene and Antibody Therapy, State Key Laboratory of Chemical Oncogenomics, State Key Laboratory of Health Sciences and Technology, Institute of Biopharmaceutical and Health Engineering (iBHE), Shenzhen International Graduate School, Tsinghua University, Shenzhen, 518055 China; 3 Department of Plant Biotechnology and Bioinformatics, Ghent University, 9052 Ghent, Belgium; 4 VIB Center for Plant Systems Biology, VIB 9052 Ghent, Belgium; 5 Orchid Research and Development Center, National Cheng Kung University, Tainan City, 701 Taiwan; 6 Center for Genomics and Biotechnology, Haixia Institute of Science and Technology, Fujian Provincial Laboratory of Haixia Applied Plant Systems Biology, College of Life Sciences, Fujian Agriculture and Forestry University, 350002 Fuzhou, China; 7 BGI Genomics, BGI-Shenzhen, Shenzhen, 518083 China; 8 Henry Fok College of Biology and Agriculture, Shaoguan University, Shaoguan, 512005 China; 9 Institute of Tropical Plant Sciences and Microbiology, National Cheng Kung University, Tainan, 701 Taiwan; 10 Department of Life Sciences, National Cheng Kung University, Tainan, 701 Taiwan; 11 Department of Biological Sciences, National Sun Yat-sen University, Kaohsiung, 80424 Taiwan; 12 Department of Applied Chemistry, National Pingtung University, Pingtung City, Pingtung County 900003 Taiwan; 13 PubBio-Tech, Wuhan, 430070 China; 14 State Key Lab for Conservation and Utilization of Subtropical AgroBiological Resources and Guangxi Key Lab for Sugarcane Biology, Guangxi University, Nanning, 530004 China; 15 Centre for Microbial Ecology and Genomics, Department of Biochemistry, Genetics and Microbiology, University of Pretoria, Pretoria, South Africa; 16 College of Horticulture, Nanjing Agricultural University, Academy for Advanced Interdisciplinary Studies, Nanjing, 210095 China; 17 Institute of Vegetable and Flowers, Shandong Academy of Agricultural Sciences, Jinan, 250100 China; 18 Zhejiang Institute of Subtropical Crops, Zhejiang Academy of Agricultural Sciences, Wenzhou, 325005 China

**Keywords:** Genome, DNA sequencing, Plant evolution, Phylogenetics

## Abstract

Monocots are a major taxon within flowering plants, have unique morphological traits, and show an extraordinary diversity in lifestyle. To improve our understanding of monocot origin and evolution, we generate chromosome-level reference genomes of the diploid *Acorus gramineus* and the tetraploid *Ac. calamus*, the only two accepted species from the family Acoraceae, which form a sister lineage to all other monocots. Comparing the genomes of *Ac. gramineus* and *Ac. calamus*, we suggest that *Ac. gramineus* is not a potential diploid progenitor of *Ac. calamus*, and *Ac. calamus* is an allotetraploid with two subgenomes A, and B, presenting asymmetric evolution and B subgenome dominance. Both the diploid genome of *Ac. gramineus* and the subgenomes A and B of *Ac. calamus* show clear evidence of whole-genome duplication (WGD), but Acoraceae does not seem to share an older WGD that is shared by most other monocots. We reconstruct an ancestral monocot karyotype and gene toolkit, and discuss scenarios that explain the complex history of the *Acorus* genome. Our analyses show that the ancestors of monocots exhibit mosaic genomic features, likely important for that appeared in early monocot evolution, providing fundamental insights into the origin, evolution, and diversification of monocots.

## Abstract (web summary)

Acorales is sister to all other monocots and contains only one family with just one genus, *Acorus*. Here, the authors assemble the genome of the diploid *Ac. gramineus* and the tetraploid *Ac. calamus*, reconstruct an ancestral monocot karyotype and gene toolkit, and discuss the origin and evolution of the two species and other monocots.

## Introduction

With >85,000 species, representing about 21% of the world’s plant species, monocots form one of the most species-rich, ecologically dominant, and economically important lineages of land plants^1^. Monocots are renowned for their specialized morphological traits, show a huge diversity of terrestrial growth forms, have been successful colonizers of a wide variety of different habitats, and directly and indirectly form the basis for most of the human diet in the form of grain or food crops such as rice, wheat, and maize. Understanding the origin and patterns of morphological divergence, geographic diversification, and ecological adaptation of monocots is therefore of interest to a great number of plant and evolutionary biologists.

Based on morphological and molecular data, monocots are classified into 77 families and 12 orders^2,3^. They differ from other angiosperms because they have one cotyledon in the embryo, vascular bundles in the stem that are star-scattered with only primary tissue while the cambia and secondary xylem are absent. The monocot order Acorales is sister to all other monocots and contains only one family, Acoraceae^1^, with just one genus, *Acorus*. Because of its unique phylogenetic position, comparative analysis with other angiosperms could yield important insights into key evolutionary innovations during the evolution of monocots, such as vascular cambia and secondary xylem development and cotyledon development. In addition, *Acorus* has a complex evolutionary history^4–7^. Although officially only two species—with three varieties—have been accepted by Plants of the World Online^5^, four to five species and a few dozen of varieties have been suggested in *Acorus*^4^. The two accepted species, i.e., *Acorus gramineus* Solander ex Aiton and *Ac. calamus* Linnaeus (Supplementary Fig. 1), are confined to the humid areas of temperate, tropical, and subtropical Asia and North America^6^. On top of that, species and varieties in *Acorus* have different ploidy levels. *Ac. calamus* L., for instance, has been acknowledged to have diploid, triploid, and tetraploid varieties, suggesting that genome sequences of *Acorus* are of importance to understand polyploid formation and evolution in the genus, as well as the flower development and adaptation to wetland environments.

Here, we present the complete genome sequences of the diploid species *Ac. gramineus* and the tetraploid species *Ac. calamus*. Comparing the genomes of *Acorus* and other angiosperms, especially the ones from other monocots, allows us to understand the origin and evolution of the two species in *Acorus* and reconstruct the ancestral monocot gene toolkit, hence providing insights into the origin, evolution, and diversification of monocots.

## Results and discussion

### Genome sequencing and genome characteristics

Chromosomes of *Acorus gramineus* and *Ac. calamus* were fluorescently dye-stained. The diploid *Ac. gramineus* has a karyotype of 2n = 2× = 24, while *Ac. calamus* has a karyotype of 2n = 4× = 44 (Supplementary Fig. 2). Flow cytometry analyses estimated that *Ac. gramineus* has a genome size of 362.01 Mb and *Ac. calamus* has a genome size of 747.46 Mb (Supplementary Fig. 3). To sequence both genomes as completely as possible, we used PacBio Sequel and generated a total of 57.12 Gb and 86.45 Gb of raw reads for *Ac. gramineus* and *Ac. calamus*, respectively. The average lengths of the reads are 13.03 kb for *Ac. gramineus* and 13.27 kb for *Ac. calamus* (Supplementary Table 1). Through *K*-mer analysis using Smudgeplot and GenomeScope2^8^, we found that AB type *K*-mer pair of *Ac. gramineus* had a proportion of up to 60%, indicating that *Ac. gramineus* was a diploid and estimated the genome size at 409.66 Mb (Supplementary Note 1, Supplementary Fig. 4). As expected, *Ac. calamus* had AABB type *K*-mer pair with a higher proportion (43% AABB type vs 23% AAAB type), which is congruent with the fact that *Ac. calamus* was an allotetraploid, and the average size of two subgenomes is estimated as 348.65 Mb (Supplementary Note 1, Supplementary Fig. 5). The total length of the assembled genome was 391.63 Mb with a contig N50 value of 1.74 Mb for *Ac. gramineus*, and 700.94 Mb with a contig N50 value of 0.87 Mb for *Ac. calamus*. The lower contig N50 value of *Ac. calamus*, compared with that of *Ac. gramineus*, is due to its allotetraploid nature, containing more polymorphic loci, leading to more ‘bubble’ structures in the assembly graphs (Supplementary Table 2). We further evaluated the quality of the two genome assemblies by Benchmarking Universal Single-Copy Orthologs (BUSCO)^9^ and obtained BUSCO scores of 96.34% ‘complete genes’ for *Ac. gramineus* and 96.10% ‘complete genes’ for *Ac. calamus*. In line with *Ac. calamus* being a tetraploid, 72.18% of the BUSCO genes were found ‘duplicated’ in the *Ac. calamus* genome, compared to 6.07% in the diploid *Ac. gramineus* genome (Supplementary Table 3). Compared with complete BUSCO scores of 98.64% for rice, 98.20% for *Setaria viridis*, 98.02% for *Zea mays* B73, and 98.14% for *Z. mays* SK, the BUSCO assessments suggest that both *Acorus* genome assemblies are nearly complete with respect to gene space (Supplementary Fig. 6; Supplementary Tables 3, 4).

**Fig. 1:**
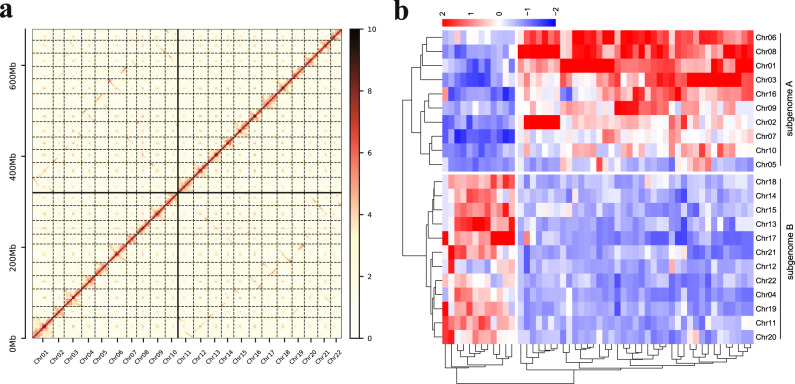
Hi-C scaffolding of the allotetraploid *Ac. calamus* genome and subgenome reconstruction. **a** Hi-C contact matrix of the 22 chromosomes in the *Ac. calamus* genome. We used Illumina sequencing reads from Hi-C libraries to reconstruct physical maps by ordering and clustering the assembled scaffolds into 22 pseudomolecules in the haploid genome of *Ac. calamus*. The vertical colorbar on the right of the axis indicates the logarithm (log2) of chromatin contact frequency. **b** Subgenome construction (see Methods). The 22 pseudomolecules of *Ac. calamus* were divided into two subgenomes, subgenome A including Chr01, 02, 03, 05–10 and 16, subgenome B including Chr04, 11–15 and 17–22 (Supplementary Table 8). The colorbar at the top indicates the Z-scaled relative abundance of *k*-mers, the larger the Z score, the higher the relative abundance of a *k*-mer. Source data are provided as a Source Data file.

To reconstruct physical maps, we further generated 50.43 Gb and 66.30 Gb reads from two Hi-C libraries of *Ac. gramineus* and *Ac. calamus*, respectively (Supplementary Table 5), and clustered and ordered the assembled contigs into pseudomolecules (Fig. 1a; Supplementary Fig. 7). The chromatin interaction results showed that the interaction signal intensity for diagonal positions was higher than for non-diagonal positions, suggesting that both the *Ac. gramineus* and *Ac. calamus* assemblies based on the Hi-C data are of high quality (Supplementary Fig. 7). For *Ac. gramineus*, the lengths of the 12 pseudochromosomes ranged from 13.73 Mb to 32.55 Mb, with a scaffold N50 value of 24.59 Mb (Supplementary Tables 6 and 7).

The allotetraploid genome of *Ac. calamus* was scaffolded using Hi-C read pairs into 22 pseudomolecules (see Methods). The Hi-C contact matrix shows a high quality of the chromosome assembly according to the chromatin contacts within one chromosome. Also, because only uniquely mapped Hi-C reads were used, traces of chromatin contacts between two chromosomes hint at some, if not all, homoeologous chromosomes from the two subgenomes of the allotetraploid *Ac. calamus* (Fig. 1a). Because only uniquely mapped Hi-C reads were used, linear traces of chromatin contacts between two chromosomes rather than within one chromosome left somewhat clues about homoeology between two chromosomes. Further, we clustered the 22 chromosomes based on the specific consensus sequences of 13-mer sequence to assign the chromosomes into the two subgenomes based on Mitros et al.^10^ (see Methods, and code executed in Codes 1–8 [https://github.com/2017dingkun/Acorus-genome]). As a result, 10 and 12 chromosomes were sorted as subgenomes A and B, respectively (Fig. 1b), in line with the observed linear traces of chromatin contacts (Fig. 1a). In addition, the results generated by SubPhaser^11^ were consistent with the above subgenome assignment (see Methods; Supplementary Fig. 8). *Ac. calamus* subgenome A (referred to as *Ac. calamus* A below) amounts to 323.33 Mb, with a contig N50 size of 0.74 Mb. The lengths of the ten pseudochromosomes ranged from 21.69 Mb to 45.83 Mb with a scaffold N50 value of 29.86 Mb. *Ac. calamus* subgenome B (referred to as *Ac. calamus* B below) amounts to 360.99 Mb, with a contig N50 size of 0.70 Mb. The lengths of the 12 pseudochromosomes ranged from 24.30 Mb to 33.96 Mb, with a scaffold N50 value of 29.84 Mb (Supplementary Tables 6, 8). In addition, analysis of the distribution of tandem repeat showed that the putative centromeric region could be detected in 16 of 22 *Ac. calamus* chromosomes and 6 of 12 *Ac. gramineus* chromosomes, the assembly of *Ac. calamus* A and B might have more complete centromeric regions than *Ac. gramineus* (Supplementary Fig. 9).

A total of 198.59 Mb, 145.66 Mb and 167.52 Mb of repetitive elements from *Ac. gramineus*, *Ac. calamus* A, and *Ac. calamus* B, respectively, were annotated using a combination of structural information and homology prediction (Supplementary Table 9). The results showed that the percentages of de novo predicted repeats in *Ac. gramineus* (47.13%), *Ac. calamus* A (42.18%) and *Ac. calamus* B (42.96%) were much higher than the predicted repeats based on homology in *Ac. gramineus* (6.01%), *Ac. calamus* A (5.09%) and *Ac. calamus* B (5.02%) obtained by Repbase (v21.12)^12^, indicating that *Ac. gramineus* and *Ac. calamus* (A and B) have many unique repeats undocumented in the Repbase library (version 20170127)^12^ (Supplementary Table 10; Supplementary Figs. 10–12). Further, Extensive *De-novo* TE Annotator (EDTA)^13^ substantiated the high percentages of de novo TEs after filtering false positives in the de novo TE predictions. The classification of repeat sequences showed that a substantial part of the *Ac. gramineus* and *Ac. calamus* genomes contain retrotransposable elements, and the most abundant subtypes are *Copia* and *Gypsy* (Supplementary Tables 11–14).

We annotated 25,090, 21,743 and 24,322 protein-coding genes for *Ac. gramineus*, and *Ac. calamus* A and B, respectively (Supplementary Table 15**)**. We used BUSCO to assess the completeness of the gene prediction and identified 94.98% of the complete set of BUSCO genes in *Ac. gramineus*, and 94.48% of the complete set of BUSCO genes in *Ac. calamus* with 81.78% in *Ac. calamus* A and 81.79% in *Ac. calamus* B (Supplementary Table 16). The number of complete BUSCO genes of each *Ac. calamus* subgenome is lower than that of the combination of the two *Ac. calamus* subgenomes, suggesting that each subgenome has been undergoing reciprocal gene loss after allopolyploidization^14–18^.

We further identified the noncoding RNAs of the *Ac. gramineus* and *Ac. calamus* A and B genomes. There are 57, 44 and 55 microRNAs, 596, 481 and 477 transfer RNAs, 159, 96 and 38 ribosomal RNAs and 200, 170 and 184 small nuclear RNAs in the genomes of *Ac. gramineus*, *Ac. calamus* A, and B, respectively (Supplementary Table 17). We predicted the target genes for each microRNA by PsRobot_tar from psRobot (v1.2)^19^ and find 175, 87 and 101 target genes in *Ac. gramineus*, *Ac. calamus* A, and *Ac. calamus* B, respectively. The GO enrichment results showed that the target genes regulated by microRNAs are mainly involved in “protein complex”, “cell periphery” and “sequence-specific DNA binding” in *Ac. gramineus*; “nitrogen compound metabolic process” and “heterocyclic compound binding” in *Ac. calamus* A; “intracellular part” and “heterocyclic compound binding” in *Ac. calamus* B (Supplementary Figs. 13–15). In addition, KEGG enrichment results showed the target genes to be predominantly participating in “biosynthesis of amino acids” and “glycerolipid metabolism” in *Ac. gramineus*; “endocytosis”, “plant hormone signal transduction” and “ribosome” in *Ac. calamus* A; and “ubiquitin-mediated proteolysis”, “spliceosome” and “microbial metabolism in diverse environments” in *Ac. calamus* B (Supplementary Figs. 16–18). These results indicate that microRNAs are mainly involved in regulating basic amino acids and glycerolipid metabolism in *Ac gramineus*, while in *Ac. calamus*, they are mainly involved in interaction with the environment^20^.

### Evolution of gene families

**Fig. 2:**
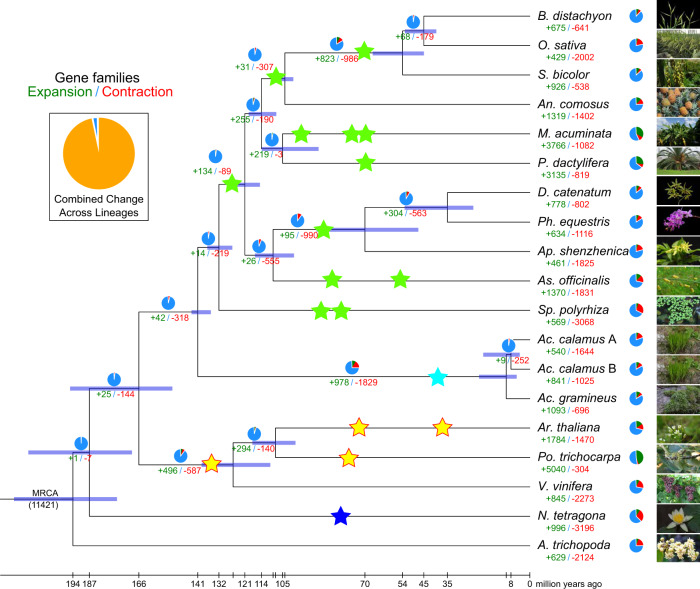
Phylogenetic tree showing divergence times and the evolution of gene family size in 19 species. The green and red numbers are the numbers of expanded and contracted gene families, respectively. The blue portions of the pie charts represent the gene families whose copy numbers are constant. The orange portions of the pie charts represent the proportion of the 11,421 gene families found in the most recent common ancestor (MRCA) that expanded or contracted during recent differentiation. Star, showing the time and position of genome polyploidy event, the blue, yellow, cyan and green stars represent *N. tetragona*, core eudicot, *Acorus* and other monocots, respectively. For each branch, the pie chart shows the gene families number of contracted (green), expanded (red) and stable (blue).

We constructed a high-confidence phylogenetic tree and estimated the divergence times of 19 different plant species based on the nucleotide and amino acid sequences from a total of 379 single-copy gene families (see Methods, Supplementary Note 2 and Supplementary Table 18). The phylogenetic trees constructed by both the concatenated and coalescent methods were similar and showed that *Ac. gramineus* is sister to *Ac. calamus* A and B (Fig. 2, Supplementary Fig. 19). As expected, *Acorus* forms an independent clade, i.e., Acorales, as a sister group to all other monocots (Fig. 2, Supplementary Fig. 19). Expansion and contraction of orthologous gene families were determined by CAFÉ v4.2.1 (https://github.com/hahnlab/CAFE)^21^. A total of 42 and 496 gene families were expanded while 318 and 587 families became contracted in the lineage leading to the monocots and eudicots, respectively (Fig. 2). In the lineage leading to Acorales, 978 gene families were expanded, whereas 1829 families were contracted. In *Ac. gramineus* 1093 were expanded, a larger number than the 540 expanded gene families in *Ac. calamus* A and the 841 expanded gene families in *Ac. calamus* B. In contrast, a smaller number of contracted gene families, i.e., 696, was observed in *Ac. gramineus*, compared to 1644 in *Ac. calamus* A, and 1025 in *Ac. calamus* B, which is likely due to the substantial gene loss after the polyploidization of the *Ac. calamus* genome (Fig. 2). As indicated by the BUSCO analyses above, both *Ac. calamus* A and B have lost more genes than *Ac. gramineus*. After becoming allotetraploid, it seems that both subgenomes of *Ac. calamus* have lost genes reciprocally. For instance, 185 of the 242 missing BUSCOs in the subgenome A retain in the subgenome B, while 94 of the 151 missing BUSCOs in subgenome B retain in the subgenome A, leading to the gene family contractions in subgenomes A and B^15,22^.

To reveal the effects of gene loss and gain during the formation of the most recent common ancestor (MRCA) of monocots, we performed GO enrichment analysis for the 28 significantly changed gene families on the branch leading to the MRCA of monocots. We found that the 20 significantly contracted gene families were enriched in the terms ‘transferase activity, transferring hexosyl groups’, ‘iron ion binding’, and ‘catalytic activity’ (Supplementary Data 1), probably reflecting the decoration of hexoses at iron ion binding activity that might not be active in monocot species. The eight significantly expanded gene families were enriched in ‘O-methyltransferase activity’, ‘fatty acid biosynthetic process’ and ‘protein dimerization activity’ (Supplementary Data 2), implying that the transfer of the methyl group to the oxygen atom of acceptor molecules and biosynthesis of diversified fatty acids might contribute to unique monocot characteristics such as the formation of rhizomes and habitat at wetland zone. The KEGG enrichment results showed that 20 significantly contracted gene families were particularly enriched in the ‘cytochrome P450’ and ‘oxidative phosphorylation’ pathways (Supplementary Data 3). The eight significantly expanded gene families were enriched in the KEGG pathways of ‘sphingolipid metabolism’, and ‘metabolism of xenobiotics by cytochrome P450’ (Supplementary Data 4). Sphingolipids act as physiological mediators regulating ABA-dependent guard cell closure, programmed cell death, pathogen resistance, and cold stress signalling^23^. Plants have the ability to produce a vast array of metabolites by versatile cytochrome P450 activity to protect themselves as well as affect other organisms in the same ecosphere^24^. KEGG enrichment of these two pathways implies that sphingolipids and xenobiotics produced in monocots might play important roles in adaptation to wetland growth niches. We also identified gene families that were present in 14 monocot genomes but absent in the genomes of five non-monocot species, and found seven gene families enriched in the GO terms ‘regulation of transcription, DNA-template’, ‘nucleosome assembly’, ‘phosphorelay signal transduction system’, and in the KEGG pathways of ‘plant hormone signal transduction’, and ‘biosynthesis of amino acids’ (Supplementary Figs. 20, 21; Supplementary Data 5). These results provide a reference for further study of biological processes and species differentiation in monocots.

### Whole-genome duplication in *Acorus* and monocots

**Fig. 3:**
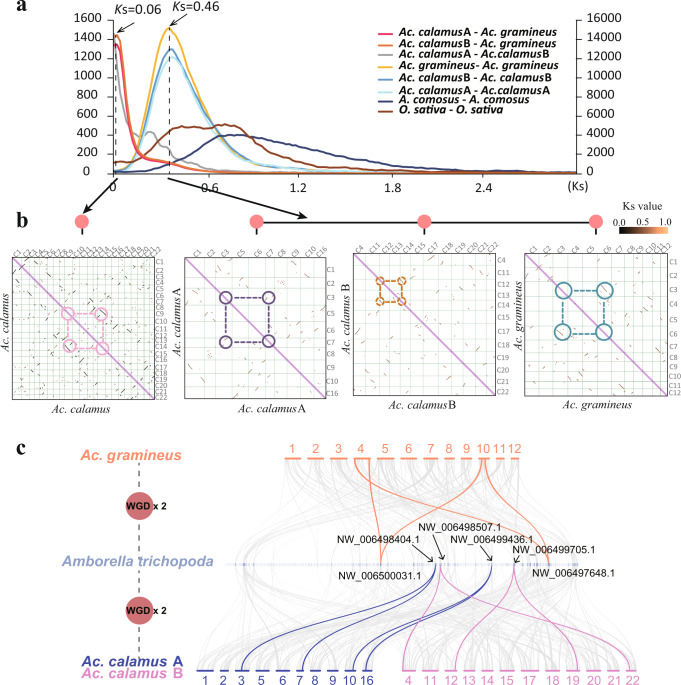
WGD in *Acorus*. **a** *Ks* distribution. Left y axis, orthologues of *Ac. gramineus*-*Ac. calamus* A, *Ac. gramineus*-*Ac. calamus* B, and *Ac. calamus* A-*Ac. calamus* B. Right y axis, paralogues of *Ac. calamus* A, *Ac. calamus* B, *Ac. gramineus*, pineapple and rice. **b** Dot plots of paralogues in the *Ac. calamus* A, *Ac. calamus* B and *Ac. gramineus* genomes illustrating the shared WGD and paralogues in the *Ac. calamus* genome clarifying the independent WGD event of *Ac. calamus* (Supplementary Fig. 24). **c** A collinear comparison of the *Ac. calamus* and *Ac. gramineus* genomes with the *Amborella* genome. Macrosynteny results showed that a collinear block pattern of 1 to 2 was retained between *Amborella* and *Ac. calamus* and between *Amborella* and *Ac. gramineus*. The corresponding contig IDs of *Amborella* are marked in the figure. Source data are provided as a Source Data file.

We constructed *K*s-based age distributions for anchor pairs, i.e., duplicated genes retained in collinear regions of a genome, uncovered in the three *Acorus* (sub)genomes (see Methods) and observed peaks that signal WGDs at *K*s values of about 0.46. The *K*s peak values of the three anchor-pair distributions are all lower than the peak value in the *K*_S_ distribution of the one-to-one orthologues between *Ac. gramineus* and *Spirodela polyrhiza* (*K*s = 1.88), indicating that the WGD signatures are specific to Acorales and not shared with other monocots (Fig. 3, Supplementary Fig. 22). Furthermore, we estimated the synonymous substitution rate in the lineage to *Acorus* as 5.26 × 10^−9^ per site per year (see Methods), hence the *Acorus*-specific WGD would have occurred at ~41.7 Mya, with a 95% confidence interval (CI) of 38.9–42.8 Mya. In turn, the *K*_S_ peak for orthologs between *Ac. gramineus* and *Ac. calamus* (A and B) is at 0.058 (Fig. 3a, Supplementary Fig. 22), implying that the divergence between *Ac. gramineus* and the common ancestor of *Ac. calamus* A and B occurred ~9.9 Mya (95% CI: 5.6–21.6 Mya), while the divergence time for the (progenitors of the) subgenomes A and B of *Ac. calamus* A and *Ac. calamus* B, with a *K*s value of 0.055 was estimated at ~8.5 Mya (95% CI: 4.4–19.8 Mya) (Fig. 2, Supplementary Fig. 22, see Methods).

Combining previously published data^25–41^, our analyses of the *Acorus* genomes suggest the following major paleopolyploidy events during the evolution of monocots (Fig. 2): (1) the τ WGD was shared by most monocots and supposed to have occurred ~120 Mya (95% CI: 110–135 Mya)^29^, (2) the σ WGD was shared by all Poales and supposed to have occurred ~110 Mya (95% CI: 100–120 Mya)^29,34,35,39^, (3) the two consecutive WGDs SPα and SPβ in the lineage leading to Sp. *Polyrhiza* within a short period of time about 95 Mya^30,40^, (4) the ρ WGD was shared by all Poaceae and supposed to have occurred ~70 Mya^36^, (5) the orchid WGD was shared by all orchids and also supposed to have occurred ~74 Mya (95% CI: 72–78 Mya)^32^, (6) the *Acorus* WGD, which have occurred ~41.7 Mya in the common ancestor of *Ac. gramineus* and *Ac. calamus*. All the ancient WGDs reported in monocots above are younger than the divergence between Acorales and other monocots, estimated to be at ~140 Mya (95% CI: 135–144 Mya), which agrees with the fact that we could not detect any signal for WGD events older than the *Acorus* specific ones. For instance, intergenomic collinear analyses between the two *Acorus* (sub)genomes and the *Amborella* genome, which has not experienced a WGD since the divergence of angiosperms, only showed two collinear segments in an *Acorus* (sub)genome to one collinear segment in the *Amborella* genome, in support of a single WGD duplication shared by *Acorus* (Fig. 3c, Supplementary Fig. 23).

### Karyotype evolution in *Acorus* and monocots

**Fig. 4:**
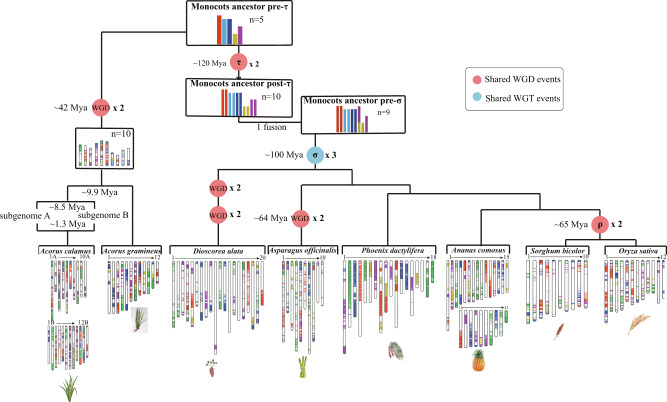
The karyotype evolution in monocots. Evolutionary scenario of *Acorus* and other representative angiosperm plants from the most recent common ancestor (MRCA) of 15 protochromosomes. After the divergence of monocots and eudicots, eudicots have been suggested to consist of seven (pre-γ ancestor) or 21 (post-γ ancestor) protochromosomes, with γ indicating an ancestral whole-genome triplication shared by most, eudicots (WGT-γ), such as *Ar. thaliana* and *C. sinensis*. In monocots, such as *An. comosus* and *O. sativa*, consisting of five (pre-τ ancestor) or ten (post-τ ancestor) chromosomes, with τ indicating the ancient WGD shared by most monocots, of which *Acorus* did not experience τ-WGD and have five chromosomes of pre-τ ancestor and experienced a WGD until 42 Mya, leading to 12, 10 and 12 chromosomes in *Ac. gramineus*, *Ac. calamus* A and *Ac. calamus* B, respectively. Thereafter, *Ac. calamus* A and *Ac. calamus* B formed an allotetraploid *Ac. calamus* with 22 chromosomes. The composition of ancestral chromosomes in modern plant genomes is shown below, with different colours representing different ancestral chromosomes. Distant polyploidization events are represented by circles in different colours. Source data are provided as a Source Data file.

We compared chromosome structure and collinearity between *Ac. gramineus* and *Ac. calamus* (A and B) by MCSCANX^42^ (Fig. 3b, Supplementary Fig. 24 and Supplementary Tables 19, 20). We used LAST to pairwise compare the genome protein sequences and made a hits dotplot (Supplementary Fig. 25). By mapping the scaffold breaking points to dotplots between *Ac. calamus* A and *Ac. gramineus*, *Ac. calamus* B and *Ac. gramineus*, and *Ac. calamus* A and *Ac. calamus* B, we show that large collinear regions exist in all three genome comparisons. These large collinear regions do not coincide with scaffold breakpoints, suggesting high continuity of the assembled *Acorus* genomes. Furthermore, to investigate the karyotype evolution of *Acorus*, the genomes of *Arabidopsis*^43^, *Citrus sinensis*^44^, and grape^45^ were selected as representative species of eudicots, and the genomes of pineapple^29^, sorghum^46^, Sp. *polyrhiza*^40^, *Phoenix dactylifera*^41^, *Asparagus officinalis*^37^, rice^47^, *Dioscorea elata*^48^ and both *Acorus* species were selected as representative species of monocots. The grape genome is especially important in elucidating eudicot genome evolution and is considered to have the most similar to the ancestral eudicot karyotype (AEK). In turn, the oil palm genome^49^ retains a large number of ancestral monocot karyotype (AMK), so it plays a crucial role in elucidating monocot genome evolution. Then, each monocot genome was compared with oil palm while each eudicot genome was compared with grape using MCSCANX, the karyotype structure of each genome could be obtained according to the collinear blocks (see Methods, Fig. 4).

By comparing collinearity between *Ac. calamus* A, *Ac. calamus* B, and *Ac. gramineus*, we inferred the karyotype of their MRCA (Supplementary Figs. 26–29). Fusion and fission events between two chromosomes could be identified by observing the gene homology dotplot between the *Ac. calamus* subgenomes and *Ac. gramineus*^50^. We showed an example of the deduction of one of the ancestral chromosomes corresponding to Chr11 of *Ac. calamus* B, as shown in Supplementary Figs. 26–28. Chr1 of *Ac. calamus* A has well collinearity with Chr11 of *Ac. calamus* B (A1-1 and A1-3), except for A1-2 (Supplementary Fig. 28), indicating either Chr1 of *Ac. calamus* A or Chr11 of *Ac. calamus* B retained the ancestral karyotype. We further compared Chr1 of *Ac. calamus* A with *Ac. gramineus* (Supplementary Fig. 26). Chr1 of *Ac. calamus* A was divided into three segments (A1-1, A1-2 and A1-3), and A1-2 was aligned to two segments of Chr6 (G6-1 and G6-4) of *Ac. gramineus*, indicating that Chr1 of *Ac. calamus* A was rearranged after the divergence of *Ac. gramineus* and *Ac. calamus*. Together, these results show that *Ac. calamus* B Chr11 corresponding to A1-1 and A1-3 of Chr1 of *Ac. calamus* were conserved after the divergence of *Ac. gramineus* and *Ac. calamus*, and retained the ancestral karyotype. Based on these deductions, we thus reconstructed the MRCA karyotype for *Ac. gramineus* and *Ac. calamus* with ten chromosomes (Supplementary Fig. 29). We found that *Ac. calamus* B (B15 and B19 in Supplementary Fig. 29) and *Ac. gramineus* (G1 and G2 in Supplementary Fig. 29) experienced specific chromosome split events, which may explain why the chromosome number of *Ac. calamus* B and *Ac. gramineus* was 12. In summary, reconstruction of the ancestral *Acorus* genome, and considering WGD events, suggests that the number of ancestral monocot chromosomes was five. *Acorus* did not share any of the older WGDs in monocots, but experienced a lineage-specific WGD at ~41.7 Mya (see above), which ultimately led to 12, 10 and 12 chromosomes in *Ac. gramineus*, *Ac. calamus* A and *Ac. calamus* B, respectively. Next, progenitors of *Ac. calamus* A and *Ac. calamus* B formed an allotetraploid with 22 chromosomes (Fig. 4).

### Allotetraploid formation

**Fig. 5:**
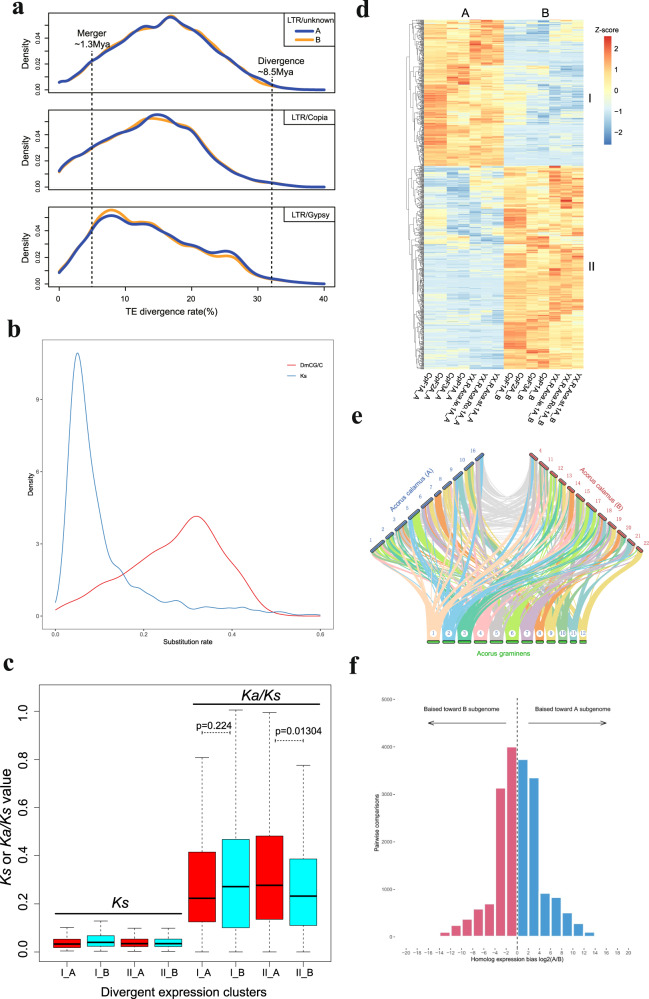
The time of allotetraploidization and DNA methylation in homoeologous expression bias in *Ac. calamus*. **a** The distribution of sequence divergence rates of TEs as percentages of subgenome size of *Ac. calamus*. The TE content segregation between subgenomes A and B indicates the events of diploid progenitor divergence ( ~ 8.5 Mya) and subgenome merger ( ~ 1.3Mya) in tetraploid of *Ac. calamus*. **b** The synonymous substitution rate (*Ks*) of CG body-methylated homoeologous genes and the substitution rate distribution of gene-body DmCG. **c** Boxplot of the *Ka*/*Ks* ratio distribution of homoeologous genes of two extremely divergent expression clusters in two subgenomes as show in **d**. **d** Heatmaps of two extremely divergent co-expression clusters, of which one of two homoeologous genes in subgenome A or B was extensively transcribed while the other copies suppressed in seven tissues (flower, inflorescence, peduncle, leaf, root, bract, and stem). Cluster I, presents A bias genes; Cluster II, present B bias genes. FPKM, fragments per kilobase of exon per million fragments mapped for each predicted transcript. **e** Collinearity of *Ac. calamus* subgenome A, *Ac. calamus* subgenome B and *Ac. gramineus* after the WGD and in specific. **f** The distribution of homolog expression bias (HEB) of homologous gene pairs in all tissues of *Ac. calamus* A and B. HEB > 0 indicates a bias toward the subgenome A, and HEB < 0 indicates a bias toward the subgenome B. Source data are provided as a Source Data file.

*Ac. calamus* resulted from a hybridization of two ancestral diploid *Acorus* species, the GenomeScope analysis based on *K*-mer above also provides support for the allopolyploidization event (Supplementary Fig. 4). Phylogenetic analysis shows that *Ac. calamus* A and B form a clade and are sister to *Ac. gramineus*, and that the two parents have diverged around 8.5 Mya (95% CI: 4.4–19.8 Mya) (Fig. 2). Therefore, we believe these results provide substantial evidence for an allotetraploid origin of *Ac. calamus*. Because there is only one extant diploid *Acorus* species left, it is challenging to identify the diploid ancestral lineages and to unveil the exact evolutionary origin of *Ac. calamus*. However, the low consistency of collinearity between *Ac. calamus* subgenomes A and B and the genome of extant *Ac. gramineus* (Fig. 5e, Supplementary Figs. 29, 30) suggests that both subgenome progenitors have been derived from quite different diploid lineages, not closely related to *Ac. gramineus*. Likely, these progenitors are extinct diploids from a relatively distant lineage in *Acorus*. Then, to estimate the age of the allopolyploidy event, i.e., the hybridization event when the two parental genomes were merged, we collected transposable elements (TEs) from the two subgenomes of *Ac. calamus* and assessed their divergence rates^15,51^ (Supplementary Fig. 11). The TE sequence divergence between the two subgenomes of the tetraploid *Ac. calamus* shows a high degree of overlap, which suggests the consistency of the TE evolutionary rate in two subgenomes^18^ (Fig. 5a). The non-overlapping segregation region indicates the time frame from the divergence between the two diploid progenitors (estimation of 8.5 Mya) to the allopolyploidy event when the two progenitors hybridized as a tetraploid genome at 1.3Mya (Fig. 5a; see Methods).

### Asymmetric evolution of subgenomes in the allotetraploid *Ac. calamus*

Subgenome dominance occurs when one of the subgenomes has genes showing higher expression, experiencing stronger purifying selection, or maintaining lower levels of DNA methylation than the other subgenome^15,52,53^. In *Ac. calamus*, subgenome A has ten chromosomes with a total length of 318.86 Mb and 21,743 genes, while subgenome B has 12 chromosomes with a total length of 360.79 Mb and 24,322 genes. Gene family clustering analysis identified 13,754 homoeologous gene pairs between *Ac. calamus* subgenomes A and B (see Supplementary Note 3, Methods), enabling us to compare the expression profiles of the homoeologous pairs from the two subgenomes A and B in seven tissues, i.e., the flower, leaf, stem, root, bract, peduncle, and inflorescence base. We analyzed the expression bias of the homoeologous pairs in subgenome A and B and identified homoeologous pairs with biased expression, i.e., higher gene expression towards subgenome A or B (Fig. 5d). Our results show more homoeologous gene pairs with biased expressions towards subgenome B than those with biased expression towards subgenome A across the seven sampled tissues (see Supplementary Note 3, Methods; Fig. 4d, f; Supplementary Figs. 31, 32; Supplementary Table 21). The results hence suggest that the *Ac. calamus* subgenome B is the dominant subgenome with, in general, genes that are expressed at higher levels than genes of subgenome A. Interestingly, despite subgenome B being dominant, the differently expressed homoeologous pairs in the two subgenomes also have different functions. Our GO enrichment analyses show that the biased expressed homoeologous pairs towards subgenome A are mainly involved in ‘diacylglycerol kinase activity’, ‘protein kinase C-activating G-protein coupled receptor’, ‘signaling pathway’ and ‘protein phosphatase 1 binding’, while the biased expressed homoeologous pairs towards subgenome B are mainly involved in ‘intramolecular transferase activity’, ‘metabolic process’ and ‘riboflavin biosynthetic process’ (Fig. 5d; Supplementary Table 22).

We then calculated the ratio of nonsynonymous substitutions to synonymous substitutions for each homoelogous gene pair to evaluate selection pressure on genes of both subgenomes. Selection pressure on these 808 homologs (338 and 470 genes that are subgenome A biased and B biased, respectively) with extreme divergent expression in the two subgenomes (Supplementary Data 6) showed that the dominantly transcribed homoeologous copies, regardless of their subgenome location, were more likely experiencing stronger purifying selection than their homoeologs (Mann–Whitney *U*-test, I_A/I_B, *p*-value = 0.224, II_A/II_B, *p*-value = 0.013) (Fig. 5c).

Some studies on polyploid species have shown that the difference in subgenome methylation is also related to subgenome dominance^54,55^. Hence, we compared the distributions of mCG, mCHG, and mCHH in the 13,754 homoeologous protein-coding genes with their 2 kb upstream and downstream regions in the two *Ac. calamus* subgenomes (see Methods). The results showed that the CG methylation level in subgenome B was lower than that in subgenome A in both the upstream and downstream regions (Wilcoxon rank-sum test, *P-*value = 0.0187), but subgenome B had higher CG methylation levels than subgenome A in the gene bodies (Wilcoxon rank-sum test, *P*-value = 4.8E-4). Both subgenomes had similar CHG and CHH methylation levels in the upstream and downstream regions (Wilcoxon rank-sum test, *P-*value > 0.05) (Supplementary Fig. 33), while the CHG methylation level in the gene bodies of subgenome B was higher than that in subgenome A, and the CHH methylation levels in the gene bodies of subgenome B was lower than that in subgenome A (Wilcoxon rank-sum test, *P*-value = 0.0483) (Supplementary Table 23).

Previous studiehave shown that the hypermethylated methylation level of gene body CG mostly appeared in conservative constitutively expressed genes^56,57^. Therefore, it is possible that more constitutively expressed genes were retained in the *Ac. calamus* subgenome B. CG hypermethylation in the promoter region usually inhibits gene expression, while the methylation level of the upstream and downstream regions in subgenome B is lower than that of subgenome A (Supplementary Table 23). Therefore, the genes showing the subgenome B expression bias may be the result of the lower and higher CG methylation in the promoter regions and gene bodies of subgenome B, respectively.

To explore the relationship between methylation and sequence evolution in protein-coding regions, we obtained the *P-*value of each gene’s methylation level by a binomial test, and homoeologous genes (both genes in a homologous pair are methylated) with *P*_CG_ < 0.05 were selected as CG body-methylated genes. To reduce the influence of non-CG methylation, we eliminated genes with *P*_CHG_ < 0.05 and *P*_CHH_ < 0.05 and finally obtained 1513 CG gene body-methylated homoeologous genes. The *K*s distribution and DmCG/C distribution of these genes shows that the rate of CG methylation changes in these genes (DmCG/C) was significantly higher than the substitution rate (*K*s) of the coding regions (Fig. 5b, Supplementary Table 23), indicating that after the two subgenomes diverged, and the methylation substitution rate was higher than the rate of synonymous substitutions^13^.

The above analyses suggest that subgenome B of *Ac. calamus* is dominant over subgenome A. Compared with subgenome A, subgenome B has lost fewer genes, underwent stronger purifying selection, and has a higher expression of genes and reduced CG methylation levels in the promotor region, indicating asymmetrical genome evolution of tetraploid *Ac. calamus* after genome merging.

### The evolution of unique morphological traits

MADS-box genes are known to be involved in many important processes during plant development but are especially known for their roles in flower development^58^. Because *Acorus* is a sister group to the rest of the monocots and is famous for its flower morphology with six tepals and stamens in two whorls of three, we focused on identifying and characterizing the MADS-box genes in more detail. In total, 90 and 90 putative MADS-box genes were identified in *Ac. gramineus* and *Ac. calamus* with 45 in subgenome A and 45 in subgenome B, respectively (Table 1 and Supplementary Data 7, 8). The numbers of MADS-box genes in the two *Acorus* genomes were higher than those found in other monocots, such as rice (74 members)^59^, *Phalaenopsis equestris* (51 members), and *Apostasia shenzhenica* (36 members)^32,60^. Interestingly, the tetraploid *Acorus* has the same number of MADS-box genes as the diploid *Acorus* species. We identified 63 type I MADS-box genes in *Ac. gramineus* and 42 type I MADS-box genes in *Ac. calamus*, with 22 in subgenome A and 20 in subgenome B, which were further classified into three subfamilies: Mα, Mβ, and Mγ (Table 1). Tandem gene duplications seem to have contributed to the increase in the number of type I MADS-box genes^61^ and suggest that type I M*α* and M*γ* genes have been mainly duplicated by smaller-scale and more recent duplications (Supplementary Figs. 34, 35).

**Table 1:** MADS-box genes in *Ar. thaliana*, Sp. *polyrhiza*, *Ap. shenzhenica*, *Ph. equestris*, *O. sativa*, *Ac. gramineus*, *Ac. calamus* A and *Ac. calamus* B

Category	*Ac. gramineus*	*Ac. calamus* A	*Ac. calamus* B	*Ap. shenzhenica*	*Ph. equestris*	Sp. *polyrhiza*	*Ar. thaliana*	*O. sativa*
Type II (Total)	27	23	25	27	29	20	45	43
MIKCc	22	19	19	25	28	18	38	37
MADS*	5	4	6	2	1	2	7	6
A	2	3	2	2	3	2	4	4
Bs	4	2	2	1	1	1	2	3
AP3	1	1	1	2	4	0	1	1
PI	1	1	1	1	1	2	1	2
C/D	4	3	3	4	5	3	4	5
E	2	2	1	3	6	1	4	5
AGL6	1	1	2	2	3	1	2	2
AGL15	0	0	0	0	0	0	2	1
FLC	0	0	0	0	0	0	6	0
OsMADS32	1	0	1	1	0	0	0	1
AGL12	2	3	2	1	0	0	1	2
SOC1	1	0	1	2	2	0	5	3
ANR1	2	2	2	4	2	1	4	5
SVP	1	1	1	2	1	7	2	3
Type I (Total)	63	22	20	9	22	20	58	31
Mβ	1	1	1	0	0	6	17	9
Mγ	21	10	10	4	12	3	21	10
Ma	41	11	9	5	10	11	20	12
Total	90	45	45	36	51	40	103	74

*Ac. gramineus* has 27 type II MADS-box MIKC^c^ genes and five MIKC* genes, and *Ac. calamus* has 38 type II MADS-box MIKC^c^ genes with 19 genes in each of subgenomes A and B, respectively, and ten MIKC* genes with four and six in subgenomes A and B, respectively. Phylogenetic analysis showed that most of the genes in the type II MADS-box clades had been duplicated, except those in the *B-PI*, *AP3*, *AGL6*, *SOC1*, *SVP*, and *OsMADS32* clades. In addition, *FLC* and *AGL15* clade genes could not be found in *Acorus* (Supplementary Fig. 34). *Ac. gramineus* and *Ac. calamus* have four *Bs*-like genes, more than other sequenced monocot genomes (Table 1). The *Bs* gene is involved in the differentiation and development of ovules^62^. Type I genes have been associated with the development of the embryo, central cell, and endosperm^63–65^. The duplication of the Type II *Bs* and Type I Mα and Mγ genes may be related to the fact that the inner integument in *Acorus* forms the micropyle and is much larger than the outer integument. The *FLC* genes have been found in cereals, but they are difficult to identify because they are highly divergent and relatively short^66^. However, genes in the *AGL15* clades are present in the genomes of rice and *Arabidopsis thaliana*; therefore, orthologues of *FLC* and *AGL15* might have been specifically lost in *Acorus* and orchids^67,68^ (Supplementary Fig. 34).

The A, B-AP3/PI, C/D, and E subfamilies are the major components in the well-known ‘ABCDE’ model in flowering plants that describe their roles in the development of petals, calyx petals, stamens, and ovaries^58,69^. We further investigated the expression of MADS-box genes, based on their classifications in the ABCDE model, in both *Acorus* species, by RNA-seq analyses (Supplementary Figs. 36, 37). The results showed that the ABCDE genes were majorly expressed in reproductive organs, except that expression of A-class genes were very low (Supplementary Fig. 37). B-class *AP3*-like genes were mostly expressed in stamen and moderately in tepals, while B-class *PI*-like genes were predominantly expressed in the stamens of *Ac. gramineus* and have strong expressions in tepals and stamens in *Ac. calamus*. This suggests that the expression of B-class genes is critical for both tepal and stamen identity in early monocot floral development. As the expression of C/D-class genes in carpels is conserved in other angiosperms, their expression was mostly detected in carpel. The differentially accumulated transcripts of E-class genes could be observed in various floral organs (Supplementary Fig. 36). The expression profiles of ABCDE genes revealed in *Acorus* showed similar expression to those of rice floral identity genes, where B-class genes were predominantly expressed at second- and third whorls, C-class genes were mainly expressed at third- and central whorls, and E-class genes were expressed at all the floral whorls^61,62^. These results suggested that MADS-box genes from these subfamilies create the basic blueprint of monocot floral development and form a very interesting system to study evolution of monocot floral morphogenesis.

### Vascular cambial and secondary xylem development

Woodiness, a secondary xylem derived from vascular cambium, has been gained and lost multiple times in angiosperms but has been lost in the MRCA of all monocots. Roodt et al.^70^ constructed a network of genes involved in early vascular cambial differentiation for *Ar. thaliana* from the literature, and these genes are conserved in eudicots and monocots (Supplementary Fig. 38). Based on this network, we identified these genes and their expression in *Ac. gramineus* and *Ac. calamus* A and B, as well as species from early diverging angiosperms, magnoliids and monocots (Supplementary Table 24, Supplementary Data 9–11). Previous studies have shown that *TMO5* (*TARGET OF MONOPTEROS 5*), *BDL* (*INDOLE-3-ACETIC ACID INDUCIBLE 12*), and *BEN1* (*BRI1-5 ENHANCED 1*) play important roles in the differentiation of early vascular cambia^70,71^. We found that these genes were all lost in *Acoru*s, *Oryza sativa*, and *Z. mays* (Supplementary Table 24, Supplementary Data 11), which would suggest that these genes were already lost in the ancestors of all monocots. *OBP1* (*OBF binding protein 1*) plays an important role in development and growth and is involved in cell cycle regulation^72–74^. The results of our analysis suggested that *OBP1* was lost in the MRCA of monocots as well as in *Amborella trichopoda* and *Nymphaea colorata*. In contrast, OBP1 genes are present and conserved in the genomes of eudicot species, suggesting that the loss of these genes in monocots may be specifically associated with the absence of vascular cambium differentiation in the monocot lineage (Supplementary Fig. 38).

Although monocots lack some key genes for vascular cambium differentiation and vascular cambium activity maintenance, they do have secondary cell walls., which deposited in cell during the secondary xylem developed of poplar^75,76^. We searched for *Ar. thaliana* genes involved in secondary cell wall formation^69,73,77–79^, and these genes are highly conserved across eudicots (Supplementary Data 11). However, the *XTH16* (*XYLOGLUCAN ENDOTRANSGLUCOSYLASE/HYDROLASE 16*), *CEV1* (*CONSTITUTIVE EXPRESSION OF VSP 1*), and *AIL6/7* (*AINTEGUMENTA-LIKE 6/7*) genes were lost in monocots (Supplementary Table 24, Supplementary Data 11). Despite the high conservation of the genes involved in early xylogenesis in all dicots, monocots seem to have lost important genes associated with secondary xylem development.

In summary, for plants without a vascular cambium, such as *N. colorata* and monocot species, we analyzed the genes involved in the formation of vascular cambium, and found that the *OBP1*, *TMO5*, *REVOLUTA MOL1*, and *PEAR1* genes were absent (Supplementary Data 11). However, in comparison with other angiosperms, monocots have further lost the genes *BEN1* and *BDL*. Eudicots (*Arabidopsis* and *Populus*) retained *CLE41/44*, *CEV1*, *PRR1, AIL6/7*, which are involved in the formation of vascular cambium and secondary cell wall (Supplementary Data 11). We found that many genes involved in vascular cambia and secondary cell wall formation were retained and expanded during angiosperm evolution, particularly in magnoliids and eudicots. Similar to *N. colorata*, monocots have lost genes related to vascular cambium development, which may explain their scattered vascular bundles in the stem (Supplementary Fig. 38).

### The evolution of the cotyledon

Based on the number of cotyledons, angiosperms are classified as monocotyledonous if their embryos have only one cotyledon and dicotyledonous if their embryos have two cotyledons. There are a few exceptions, such as species in the genus *Alocasia* from Araceae, which have two cotyledons, but these are considered as derived features. The cotyledons in monocotyledons can transfer organic matter from the endosperm to the plumule, hypocotyl, and radicle and absorb nutrients, while the cotyledons in dicotyledons mainly store nutrients to ensure embryo germination^80^. We analysed genes related to cotyledon development in the sequenced genome of several species, including the early diverging angiosperms (*Amborella trichopoda* and *N. colorata*), monocots (*Alocasia*, *O. sativa*, *Z. mays*, *Ac. calamus*, and *Ac. gramineus*), and a eudicot (*Ar. thaliana*) (Supplementary Data 12), and found that these genes that regulate the development of cotyledons are conserved in angiosperms (Supplementary Table 25). Among those genes, the redundant *CUC* and *SHOOT MERISTEMLESS* (*STM*) are required for shoot apical meristem formation and cotyledon separation^81,82^. Interestingly, the *STM* gene family has expanded to three members in *Ac. gramineus*, and two in *Ac. calamus* A and two in *Ac. calamus* B, but has been lost in *O. sativa* and *Z. mays*. Compared to the investigated early diverging angiosperms and eudicots, all having three members of the CUC genes, fewer numbers of *CUC* genes were found in all the investigated monocot (sub)genomes (Supplementary Table 25). Single mutations in the *PIN-FORMED1* (*PIN1*) and *PINOID* (*PID*) genes moderately disrupt the symmetric patterning of cotyledons^82^, and the *PIN1* and *PID* double mutant displays a striking phenotype that completely lacks cotyledons and bilateral symmetry^82^. *PIN1* and *PID* were duplicated in *Ac. calamus* (*PIN1*: *Ac. calamus* A, two members, and *Ac. calamus* B, two members; *PID*: *Ac. calamus* A, two members, and *Ac. calamus* B, two members), *Ac. gramineus* (*PIN1*: two members, *PID*: two members), rice (*PIN1*: two members, *PID*: two members) and corn (*PIN1*: three members, *PID*: two members) but only single copies were found in *Amborella* and *Arabidopsis*, and *N. colorata* (*PIN1*: two members) (Supplementary Fig. 39; Supplementary Table 25).

In dicotyledonous plants, the aboveground part of the seedling exhibits bilateral symmetry, as evidenced by two symmetrically located cotyledons with the shoot apical meristem (SAM) between them^83^. We infer that the expansion of *PIN1* and *PID* genes and the contraction of *CUC* genes specifically affect SAM formation, resulting in a flat or aberrant structure at the site normally occupied by the SAM of monocots (Supplementary Table 25). We also found that the expansion of the *PIN1* genes in *N. colorata* was like that of monocots, which could explain the fact that *N. colorata*, similar to monocots, also has one cotyledon^84^, but this hypothesis requires further verification.

### Adaptation to wetland environments

It has been shown that the immune signalling complex—ENHANCED DISEASE SUSCEPTIBILITY 1 (EDS1)/PHYTOALEXIN DEFICIENT 4 (PAD4)/SENESCENCE ASSOCIATED GENE101 (SAG101)—and some key signalling pathways downstream of nucleotide binding leucine-rich repeat receptors (NLR)—such as NON-RACE SPECIFIC DISEASE RESISTANCE-1 (NDR1)—were lost in five angiosperm species including Sp. *polyrhiza*, *Z. marina*, *As. officinalis*, *Utricularia gibba*, and *Genlisea aurea*^85^. Except for *As. officinalis*, the other four species are adapted to an aquatic environment. These results indicate that minimal plant immune system required for life under water, and highlight additional components required for the life of land plants^85^. Because both aquatic monocot and eudicot species lost the same well-known immune signalling complex and both *Acorus* species live in a wetland habitat, this inspired us to adopt comparative genomics for investigating genes encoding the five components of the immune signalling complex including *EDS1*, *PAD4*, *SAG101*, *NDR1*, and *ACTIVATED DISEASE RESISTANCE-LIKE 1* (*ADR1*) in the two *Acorus* species and further compared the *Acorus* genes with those from early diverging angiosperms, monocots, and eudicots. Our results show that both *Acorus* species lost all components in the immune signalling complex except *SAG101*, which is similar to what has been observed in aquatic monocots and eudicots (Supplementary Data 13).

Interestingly, fossil evidence indicates that mycorrhizal associations have occurred since 400 million years ago and implies that fungal interactions were critical for plant terrestrialization^86,87^. In addition, preventing the formation of the immunity complex could repress rice immunity by depleting the signalling of receptor-like kinase OsCERK1 to promote establishment of AM symbiosis in rice^88^. Thus, we suggest that reduction of the number of immune signalling genes might promote *Acorus* species to develop ecological associations with symbiotic fungi for adaptation to a wet land environment.

The *Acorus* rhizome is in great demand for its essential oils, which are used in the perfumery and pharmaceutical industries^89^. The *Acorus* roots interact with endophytic fungi, such as *Penicillium citrinum* AVGE1^90^, to form the rhizome, conferring benefits to the *Acorus* species ecologically by tolerating environmental stresses^90^. We investigated the expression patterns of the biosynthetic strigolactone (SL) pathway, which is a plant hormone that helps in the establishment of symbiotic relationships between plants and fungi, in both *Acorus* species (Supplementary Fig. 40). The results show that only the expression of *MAX1* (*MORE AXILLARY GROWTH1*) orthologs (*DACA002484* and *DACA010471*) was highly induced in the stems of *Ac. gramineus*, and those of *CP_A004141* and *CP_A021526* from *Ac. calamus* A were expressed in the stem and *CP_B008100* from *Ac. calamus* B was expressed in the root of *Ac. calamus* (Supplementary Fig. 40). MAX1 in *Arabidopsis* can convert carlactone into a carboxylated metabolite, i.e., carlactonoic acid^91^. Similar to the *MAX1* orthologs in rice, two *MAX1* orthologs were also discovered in the genome of *Ac. gramineus* and both subgenomes of *Ac. calamus*, suggesting that the two *MAX1* members were already present in the common ancestor of monocots. Furthermore, one *MAX1* ortholog was specifically expressed in the stem and the other was expressed in the root of *Ac. calamus*, suggesting that the MAX1 orthologs in the two subgenomes have experienced subfunctionalization. It has been reported that the *Arabidopsis MAX1* mutant shows a shoot branching phenotype and can be fully rescued to wild type by adding strigolactone^92^. High expression of *MAX1s* in the stem of *Ac. gramineus* and *Ac. calamus* confirmed that *MAX1* genes have a biological function in inhibiting shoot branching. The reason that expressions of SL biosynthetic genes were not highly detected in the roots might be that SLs stimulate early symbiotic responses in both of symbionts but not at the stable stage of symbiosis^93^. Further study of SLs regulating symbiosis with fungi at early stage of establishment in *Acorus* will get insight into the understanding of monocots adapting to terrestrialization.

To improve our understanding on the origin and evolution of monocots, we generated chromosome-level reference genomes of two species of *Acorus*, namely the diploid *Ac. gramineus* and the tetraploid *Ac. calamus*. Both species make up the Acoraceae, a sister group of all other monocots. We uncovered that the only remaining extant diploid species within Acorales, *Ac. gramineus*, is most likely not a direct diploid progenitor of *Ac. calamus*, an allotetraploid consisting of two subgenomes, ‘A’ with 20 chromosomes and ‘B’ with 24 chromosomes. Comparison of the subgenomes of *Ac. calamus* and the genomes of *Ac. gramineus* and other monocots showed clear evidence of a WGD shared by both *Acorus* species after their divergence from other monocots. Evidence for older WGDs in the *Acorus* lineage could not be found. In addition, the *Acorus* genomes allowed us to reconstruct the ancestral karyotype of monocot chromosomes, while comparisons between the gene content of *Acorus* species and other monocots and angiosperms permitted the reconstruction of an ancestral monocot gene toolkit. Subgenome B of *Ac. calamus* has lost fewer genes than subgenome A, while genes on subgenome B experienced stronger purifying selection, have higher levels of expression and show reduced CG methylation levels in the promotor region, suggesting asymmetric evolution of the tetraploid *Ac. calamus* genome, and dominance of subgenome B. We identified gene families, gene family expansions and contractions that appeared in ancestral monocots. Our analyses showed that early in monocot evolution, species already exhibited many genomic features related to flower development and cotyledon evolution, vascular cambia, secondary xylem development, adaptation to wetland environments, providing fundamental insights into the origin, evolution and diversification of monocots.

## Methods

### Sample preparation and sequencing

The plant materials (leaves, stems, and flowers) used in this study were collected an individual from wild *Ac. gramineus* and *Ac. calamus* growing in Youxi County, Fujian Province, China (26°6′57.43″N, 118°2′27.18″E), respectively. The plant materials were cleaned with 75% alcohol and then pure water for DNA extraction. Genomic DNA was extracted based on cetyltrimethylammonium bromide (CTAB) methods. DNA sequencing was performed using PacBio to sequence a 20 kb single-molecule real-time (SMRT) DNA library on the PacBio Sequel platform (for details of SMRT DNA library construction we refer to the reference link Procedure Checklist—Preparing gDNA Libraries Using the SMRTbell Express Template Preparation Kit v2.0 (pacb.com)). SMRTbell template preparation involved DNA concentration, damage repair, end repair, ligation of hairpin adapters, and template purification, and was performed using AMPure PB Magnetic Beads (Pacific Biosciences). In the process of library construction, AMPure Beads was used to purify DNA. Finally, we obtained 57.12 Gb and 86.45 Gb PacBio data for genome assembly (read quality ≥0.80 and mean read length ≥7 kb) (Supplementary Tables 1, 2).

The tissues including the flower, inflorescence, seed, leaf, root, bract and stem from an *Ac. gramineus* individual and an *Ac. calamus* individual in wild were sampled for transcriptome sequencing. Total RNA was qualified and quality-checked using Nano Drop and Agilent 2100 bioanalyzer (Thermo Fisher Scientific). Libraries were constructed using the mRNA-seq Prep Kit (Illumina) and then sequenced by the Illumina HiSeq 4000 platform.

### Genome size estimation and sequence assembly

Before genome assembly, we used clean Illumina reads to estimate genomic features. According to the Lander-Waterman theory^94^, the genome size and heterozygosity can be calculated by the total number of *K*-mers divided by the peak value of the *K*-mer distribution. *K*-mer analysis iteratively selected K bp sequences from a continuous sequence; if the length of reads was L and the length of the *K*-mer was K, then we obtained an L-K + 1 *K*-mer. Here, we took K as 17 bp, and the 17 mer frequency table was generated by Jellyfish v2.1.4^95^. Finally, we used the GenomeScope2^8,96^ software to estimate the genome size, heterozygosity, and repeat sequence. According to the *K*-mer distribution, we found that the heterozygosity rate in *Ac. gramineus* and *Ac. calamus* was very high (Supplementary Fig. 4). With the peak as the expected *K*-mer depth and the formula

1 $${{{{{\rm{Genome}}}}}}\,{{{{{\rm{size}}}}}}={{{{{\rm{Total}}}}}}\,{K}-{{{{{\rm{mer}}}}}}/{{{{{\rm{Expected}}}}}}\,{K}-{{{{{\rm{mer}}}}}}\,{{{{{\rm{depth}}}}}}$$

the size of *Ac. gramineus* genome was estimated to be 409.66 Mb, and *Ac. calamus* two subgenomes average size at 348.65 Mb, respectively (Supplementary Fig. 4).

The *Ac. gramineus* and *Ac. calamus* genomes were assembled by PacBio reads. First, we used Falcon^97^ to correct the raw data and then used Smartdenovo v1.0^98^ to assemble the corrected data. Due to high error rate of the PacBio reads, indel and SNP errors still existed in the assembly results. The assembly results of Smartdenovo were corrected with polishing using arrows (https://github.com/PacificBiosciences/GenomicConsensus). Finally, the Illumina reads were aligned to the assembly result by bwa, and Pilon v1.22^99^ was used to correct the assembly results to further eliminate indel and SNP errors.

### The Hi-C scaffolding

The leaves were fixed in 1% formaldehyde for library construction. For Hi-C scaffolding, the cell lysis, chromatin digestion, proximity-ligation treatments, DNA recovery and subsequent DNA manipulations were performed^100^. Fixed tissue was frozen in liquid nitrogen and grounded to powder, and the cross-linked DNA was digested with restriction endonuclease Mbol or DpnII. Digested DNA was marked by incubating with biotin-dCTP resulting in blunt-ended repaired DNA strands. After interacting DNA fragments were ligated to form chimeric junctions in blunt-end ligation buffer, the cross-linking was reversed, and DNA fragments tagged with biotin were enriched with beads and then sent to Hi-C library construction. The library was sequenced on the Illumina HiSeq X platform for 150 bp paired-end reads. The Hi-C reads were aligned to the draft assembly using the BWA aln algorithm^101^ with default parameters, and the quality was then assessed using HiC-Pro v.2.8.0 (http://github.com/nservant/HiC-Pro).

We obtained 66.30 Gb raw data, which was first filtered using SOAPnuke v1.5.3 with the following parameters: filter -n 0.01 -l 20 -q 0.4 -d -M 3 -A 0.3 -Q 2 -i -G -seqType 1. Juicer was applied to align the clean data to genome, then the invalid interaction pairs, including self-circle ligation, dangling ends, PCR duplicates and other potential assay-specific artefacts, were discarded. The locations and directions of the contigs were determined by 3d-DNA (v 180922) preliminarily. The result of the first iteration of 3d-DNA was used as input for Juicerbox (v1.11.08) (available at https://github.com/aidenlab/Juicebox/wiki/Download). We visualized the Hi-C contact map and performed extensive manual curation by Juicebox to adjust, reset, and cluster the genome sequence. The resulting assembly was subjected to Pilon program for error correction. Finally, high quality chromosome-level genome was obtained including two subgenomes with ten and 12 chromosomes (Supplementary Fig. 7).

To visualize the chromatin contacts and check the assembly quality, Hi-C reads were mapped to a genome and filtered by Juicerbox (v1.11.08) (available at https://github.com/aidenlab/Juicebox). Then the genome was divided into non overlapping bin, counted the number of pairs of Hi-C reads between each two bins, and generated a cross-linking strength matrix. Then we normalized each value in the matrix with log2, and finally visualized the cross-linking strength matrix with matplotlib.

### Gene and non-coding RNA prediction

Gene prediction and functional annotation were conducted by a combination of homology-based prediction, de novo prediction and transcriptome-based prediction methods. In the homology-based prediction method, we mapped the protein sequences of three published plant genomes (*Arabidopsis thaliana*, *Zea mays* and *Oryza sativa*) onto the *Ac. gramineus* and *Ac. calamus* genomes by TBLASTN (*E*-value 1 × 10^−5^) and then used GeneWise v.2.4.1^102^ to predict the gene structures. In the de novo prediction method, the homology-based results, Augustus v.2.7^103^, GlimmerHMM v.3.02^104^ and SNAP (version 2006-07-28)^105^ were combined to predict the genes. The transcriptome data from multiple tissues were mapped onto the genome assembly using TopHat v2.1.1^106^, and then Cufflinks v2.1.1^106^ was used to assemble the transcripts into gene models. MAKER v.1.0^107^ was used to generate a consensus gene set based on the homology-based, de novo, and transcriptome-based predictions (Supplementary Table 15). Functional annotation of the predicted protein sequences was achieved by aligning protein sequences against public databases, including SwissProt, TrEMBLE and KEGG, with BLASTP (*E*-value < 1 × 10^−5^). Additionally, protein motifs and domains were annotated using the InterPro and Gene Ontology (GO) databases by InterProScan v.4.8^108^.

The tRNA genes were searched by tRNAscan-SE^109^. For rRNA identification, we downloaded the *Arabidopsis* rRNA sequences from NCBI and aligned them with the *Acorus* genomes to identify possible rRNAs. Additionally, other types of noncoding RNAs, including miRNAs and snRNAs, were identified by using INFERNAL^110^ to search the Rfam database.

### Repetitive sequences prediction

Repetitive sequence annotation was combined with homology prediction based on the Repbase Library (http://www.girinst.org/repbase) and de novo prediction based on self-sequence alignment. In the homology-based method, RepeatMasker and RepeatProteinMask v.4.1.0^111^ with the Repbase database were used to search for known repeat sequences. In the de novo prediction method, LTR_FINDER v.1.0.2^112^, PILER v.1.3.4^113^, and RepeatModeler v.1.0.3^114^ were used to construct a de novo repeat sequence database for searching repeats in the genome by RepeatMasker. To verify the percentage of de novo repeats, we employed EDTA package (https://github.com/oushujun/EDTA) for de novo TE annotation. To identity candidate centromeres, we detected tandem repeats across the genome with TRF (v4.09), the parameter is “2 7 7 80 10 50 2000 -d”. We draw the distribution along the chromosome with a window size of 100 Kb. In the distribution, we found *Ac. calamus* A and B has a more complete centromeric region than *Ac. gramineus*.

### Subgenome reconstruction

We partitioned the *Ac. calamus* genome into subgenomes A and B^10^, the details as follows. The current allotetraploid genome of *Ac. calamus* is a result of divergence and fusion of the two diploid ancestors. During the evolution process, there are specific TE insertions after their divergence and these TE sequences are the key to identify subgenomes. Chromosomes can be divided into homologous pairs based on their collinearity, therefore the chromosomes that are subject to the same subgenome should have the identical specific sequences. We used Jellyfish v2.3.0 to break the genome sequence into 13 bp sequences (13-mers), and used these sequences to identify specific sequences in subgenomes. If 13-mers that (1) present >100 times across the genome; (2) were at least twofold enriched in one member of each homoeologous chromosome pair. Clustering of counts of identified 13-mers using cluster v3.0 (http://bonsai.ims.u-tokyo.ac.jp/~mdehoon/software/cluster), that differentiates homoeologous chromosomes, enables partitioning of the genome into two subgenomes (Fig. 1b). In subgenome reconstruction, we only compared the 13-mers’ frequency between two homologous chromosome pairs.

We used the software SubPhaser^11^ to construct subgenomes, and obtained an identical result as our custom code with respect to assign chromosomes into the two subgenomes (Supplementary Fig. 8). Further, using SubPhaser, we could detect potential exchange between the two subgenomes. For instance, circles of Supplementary Fig. 8c show the inferred homoeologous exchanges between the two subgenomes, such as the one at the 3' tail of Chr10.

### Gene family identification

Single-copy gene families and multicopy gene families were obtained by identifying homologous genes and clusters of gene families. First, protein sequence data sets were constructed, including those for *Ac. gramineus*, *Ac. calamus* A, *Ac. calamus* B and 16 other plant species: *Amborella trichopoda*, *Ananas comosus*, *Apostasia shenzhenica*, *Arabidopsis thaliana*, *Asparagus officinalis*, *Brachypodium distachyon*, *Dendrobium catenatum*, *Musa acuminata*, *Nymphaea tetragona, Phalaenopsis equestris*, *Phoenix dactylifera*, *Populus trichocarpa*, *Sorghum bicolor*, *Spirodela polyrhiza*, *Vitis vinifera*, and *Oryza sativa*. Then, the protein sequences were used to perform all-against-all BLASTP searches. Because we aimed at identifying orthogroups across angiosperms and OrthoMCL^115^ can deal with mis-specified homologous sequence pairs occasionally produced by BLASTP, we filtered the BLASTP results with an E-value threshold of 1 × 10^−5^, a similarity threshold of 30%, and a coverage (alignment length divided by query sequence length) threshold of 50%. Lastly, the filtered results were used to construct orthologous groups through OrthoMCL v2.0.9^115,116^.

### Whole-genome duplication

*K*s-based age distributions for all the paralogues of *Ac. gramineus*, *Ac. calamus* A and *Ac. calamus* B were constructed^117^. We simulate the evolution of coding sequences and re-calculate synonymous distances to measure specific effects. Then, we include these effects in a population dynamics model and simulate age distributions based on *K*s values. This allows us to see how *K*s stochasticity and saturation affect the detection of whole-genome duplications. In addition, paralogous gene pairs located in duplicated segments (anchors) were identified in the chromosome-level assembled genomes of *Ac. gramineus*, *Ac. calamus* A and *Ac. calamus* B using i-ADHoRe (v3.0)^118,119^. *Ks* of homologous gene pairs was calculated using Codeml (model = 2, runmode = −2) in the PAML4.9 package. The results of *K*s distributions for *Ac. gramineus*, *Ac. calamus* A and *Ac. calamus* B are shown in Fig. 3a and Supplementary Fig. 22. *Ks* peaks were identified in *Ks* distribution by an R function (https://github.com/stas-g/findPeaks).

We selected the closest outgroup of *Acorus* and a sister branch, ((*Acorus*, Sp. *polyrhiza*), grape) and used the peak *Ks* value among the three species, the divergence time of *Acorus* and its sister branch, calculated the *Ks* rate of *Acorus* branch, which was 5.26e-9 per site per year. Therefore, the time of the *Acorus*‘s own WGD was calculated from the formula

2 $${{{{{\rm{T}}}}}}={Ks}/2{{{{{\rm{r}}}}}}$$

r means the *K*s rate, which was 5.26e-9 per site per year for *Acorus* branch.

### Phylogenetic tree construction and phylogenomic dating

To obtain a reliable phylogenetic tree, it is necessary to obtain a reliable single-copy gene dataset. Orthogroups were constructed with *Ac. gramineus*, *Ac. calamus* A, *Ac. calamus* B and 16 sequenced plant genomes (Supplementary Note 2). Single-copy gene families containing proteins <200 bp in length were filtered out. The filtered protein sequences were aligned by MUSCL v3.8.31^120^, and the CDS (coding sequence) alignment results were obtained according to the relationship between the protein and CDS. The conserved sequences were obtained from the CD alignment results using Gblocks software^121^, and the supergene was concatenated by all of the conserved sequences. A phylogenetic analysis of the dataset was performed using MrBayes^122^ under the GTR + GAMMA model with four categories (Ngammacat = 4) in the discrete Gamma model to take the heterogeneity of substitution rates among sites into consideration. It has been shown that as few as four rate categories in the discrete Gamma model are not only computationally practical but can also approximate the continuous Gamma distribution to model variable rates among sites^123^. The parameters were set to ngen = 100,000, nchains = 4, burnin = 250. The rate of sampling was every 100 generations as default.

The divergence time was estimated by MCMCtree of the PAML v.4.7^124^ package, which was used to estimate divergence times in many studies^66,125–129^. The nucleotide replacement model was the GTR model. The Markov chain Monte Carlo (MCMC) process consists of a burn-in of 500,000 iterations and 1,500,00,000 iterations with a sample frequency of 150. The default setting used other parameters. The calibration times were as follows: (1) Divergence time of *Oryza sativa* and *Brachypodium distachyon* was 40–54 Mya. (2) Divergence time of *Arabidopsis thaliana* and *Populus tomentosa* was 100–120 Mya. (3) The lower limit of the divergence time of monocotyledons and dicotyledons was 140 Mya^130^. (4) The upper limit of angiosperm formation time was 200 Mya^131^.

### Estimating the time of allopolyploidization

We collected the transposable elements (TE) from both subgenomes and assessed their divergence rates in each subgenome (Fig. 5a). TE divergence was assessed by PercDivs (Percentage of substitutions in the matching region compared with the consensus) calculated in RepeatMasker. TE sequence divergence between both subgenomes of the tetraploid *Ac. calamus* displaying a high degree of overlap suggests the consistency of the TE evolutionary rate in the two subgenomes (Fig. 5a). The non-overlapping segregation region indicator of divergence to genomes merging was tetraploid genome^51,52^.

### The biased expressed homoeologous pairs in subgenomes

We used BLAST to perform all-vs-all alignment for protein sequences of *Ac. calamus* subgenome A, subgenome B and *A. gramineus* (*E*-value < 1e-5) and clustered the results using OrthoMCL (expansion coefficient as 1.5) to obtain gene family cluster results. In cluster results, we selected single copy gene families of subgenomes A and B as their orthologous pairs. We further used Bowtie2 to align clean reads from seven tissues to reference genome sequence and calculated gene expression level via RSEM and used R package EdgeR to conduct differential gene expression analysis. Homoeologous bias expression was detected in the entire 35 tissue dataset through pairwise t-tests with significance thresholds set at *P* < 0.01, FDR < 0.05, and at least two fold-changes in average expression levels^132^.

### Karyotype evolution of *Acorus*

A comparative analysis was performed with the *Acorus*, *Arabidopsis*^43^, orange^44^, grape^45^, pineapple^29^, sorghum^46^, rice^47^, Sp. *polyrhiza*^40^, *P. dactylifera*^41^, *As. officinalis*^37^, and *Dioscorea elata*^48^ genomes. To reconstruct the karyotype evolution model of *Acorus*, we compared representative eudicots and monocots plants with grape and oil palm^49^, respectively, and inferred the chromosome composition of each species according to their collinear relationships. In detail, first, to identify syntenic blocks, we performed an all-against-all LAST^133^ and connected the LAST hits at a distance cut-off of 20 genes while requiring at least five pairs for each syntenic block using MCSCANX^42^. Then, we obtained an anchors file containing the homologs identified via LAST. According to the position of grape/oil palm gene on the ancestral chromosome karyotype, combined with the collinear relationship between grape/oil palm and analysed species, we can infer which ancestral chromosome the gene of analysed species is on. The final visualization of the karyotype result is achieved through the graphics module of MCSCANX.

For the construction of MRCA of *Acorus*, a fusion between two chromosomes could be identified by observing the dot-plot in different comparison groups^50,134,135^. For example, in Supplementary Fig. 27, the whole chromosome 11 (Chr11) of *Ac. calamus* B shows good collinearity with chromosome 1 (Chr1) of *Ac. calamus A*. But in Supplementary Fig. 26, Chr11 of *Ac. calamus* B breaks into two segments collinear with chromosome 4 (Chr4) and Chr11 of *Ac. gramineus*, respectively. So, by observing the results in Supplementary Fig. 25, we can determine the ancestor karyotype if it remains intact or breaks into two segments. In Supplementary Fig. 25, the segment G1-3 of chromosome 1, segment G4-1 and G4-4 of chromosome 4 of *Ac. gramineus* together form Chr1 of *Ac. calamus* A. Above results suggest that the ancestor karyotype remains intact structure like Chr11 of *Ac. calamus* B. And so on, we reconstructed their MRCA karyotype with ten chromosomes. We found that *Ac. calamus* B and *Ac. gramineus* experienced specific chromosome split events which may explain why the chromosome number of *Ac. calamus* B and *Ac. gramineus* was 12. Above all, we agree with the hypothesis that the ancestral chromosome number of monocots was five. The paired synonymous substitution rates (*Ks*) were calculated using the Nei-Gojobori method (https://github.com/tanghaibao/bio-pipeline/tree/master/synonymous_calculation/synonymous_calc.py).

### Evolution and expression analysis of MADS box genes

We identified MADS-box genes by searching the InterProScan^136^ results of all of the predicted *Ac. gramineus* and *Ac. calamus* (A and B) proteins. The MADS-box domain comprises 60 amino acids, which were identified for all the MADS-box genes using SMART^137^. We then aligned all of the identified genes using the ClustalW^138^ program. An unrooted neighbour-joining phylogenetic tree was constructed in MEGA5^139^ with default parameters.

### Transcriptome sequencing and assembly

For the two *Acorus* species, the total RNA was extracted from fresh plant organs (roots, stems, leaves, and flowers) using the RNAprep Pure Plant Kit, and genomic DNA was removed using RNase-Free DNase I (both from Tiangen, Beijing, China). Raw reads were generated by the Illumina platform. Transcripts were assembled from filtered reads using Trinity v.2.4.8^140^.

### Selection pressure analyses

We extracted the genes that have bias expression in seven tissues (flower, leaf, stem, root, bract, peduncle and inflorescence), yielding 338 and 470 genes that are subgenome A biased and B biased, respectively. The heatmap were generated based on the expression level (TPM) of the above 808 genes in seven tissues, showing two clusters (subgenome A bias or B bias). The homologs of these 808 genes in *Ac. gramineus* were identified based on Blast RBH, and multiple sequence alignment was performed using Muscle. The *K*a and *K*s calculation was conducted using codeml in PAML with the input tree as (SCP, *Ac. gramineus*; CP_A, *Ac. calamus_*A; CP_B, *Ac. calamus_*B). The *K*a or *K*s value for each clade was calculated using the free-ratio model, and the values were presented as a box-plot (Supplementary Note 3, Supplementary Figs. 41, 42, Supplementary Tables 26–28, Supplementary Data 14, 15).

### Methylation substitution rate of *Ac. Calamus*

We used the binomial distribution test to determine whether the cytosine loci in the genome were methylated. We used function: binom_test (*x* ≥ *k*; *n*, *p*) from scipy package to calculate the binomial distribution probability of each cytosine locus, which represents the read coverage depth of the cytosine loci, where k is the coverage depth of the methylated cytosine loci and p is the error rate. We further used the following formula to determine the methylation level of the genes.

3 $${P}_{{{{{{\rm{CG}}}}}}}=\mathop{\sum }\limits_{i={m}_{{{{{{\rm{cg}}}}}}}}^{{n}_{{{{{{\rm{cg}}}}}}}}\left({{{n}_{{{{{{\rm{cg}}}}}}}}\atop{i}}\right){p}_{{{{{{\rm{cg}}}}}}}^{i}{(1-{p}_{{{{{{\rm{cg}}}}}}})}^{{n}_{{{{{{\rm{cg}}}}}}}-i},$$

In this formula, *P*_*CG*_ represents the *P-*value of the methylation level, *n*_*cg*_ is the number of C residues at the CG loci with a read coverage depth >5, and m_cg_ is the number of C residues at the methylated CG loci with a read coverage >5. The number of C residues at the methylated CG loci in the whole genome was divided by the number of C residues at the CG loci to obtain *p*_cg_, which is the proportion of C residues at the methylated CG loci in the genome.

Gene body and methylation levels of different patterns in upstream and downstream genes were calculated by “cal_methylation_distribution_in_genic_region.py” (https://github.com/2017dingkun/Acorus-genome). The differential expression of homologous genes between subgenomes was calculated by “allelic_gene_expression_compare.py” (https://github.com/2017dingkun/Acorus-genome).

### Reporting summary

Further information on research design is available in the Nature Portfolio Reporting Summary linked to this article.

## Supplementary information

Supplementary Information

Peer Review File

Description of Additional Supplementary Files

Supplementary Data 1

Supplementary Data 2

Supplementary Data 3

Supplementary Data 4

Supplementary Data 5

Supplementary Data 6

Supplementary Data 7

Supplementary Data 8

Supplementary Data 9

Supplementary Data 10

Supplementary Data 11

Supplementary Data 12

Supplementary Data 13

Supplementary Data 14

Supplementary Data 15

Reporting Summary

## Data Availability

The raw genome and transcriptome sequencing data for *Ac. calamus* and *Ac. gramineus* have been deposited to NCBI under BioProject accession PRJNA782402. The sequencing assembly and annotation data of *Ac. calamus* and *Ac. gramineus* reported in this paper have been deposited in the Genome Warehouse in National Genomics Data Center, Beijing Institute of Genomics, Chinese Academy of Sciences/China National Center for Bioinformation under accession PRJCA017027; specifically, *Ac. calamu* is under accession number GWHCBII00000000 and *Ac. gramineus* is under accession number GWHCBIH00000000. Source data are provided with this paper.

## Source data

Source Data

## Code availability

The in-house analysis scripts have been deposited in Github [https://github.com/2017dingkun/Acorus-genome].

## References

[1] Givnish TJ (2018). Monocot plastid phylogenomics, timeline, net rates of species diversification, the power of multi-gene analyses, and a functional model for the origin of monocots. Am. J. Bot..

[2] Angiosperm Phylogeny Group. (2009). An update of the Angiosperm Phylogeny Group classification for the orders and families of flowering plants: APG III. Bot. J. Linn. Soc..

[3] Angiosperm Phylogeny Group. (2016). An update of the Angiosperm Phylogeny Group classification for the orders and families of flowering plants: APG IV. Bot. J. Linn. Soc..

[4] Cheng Z (2020). From folk taxonomy to species confirmation of *Acorus* (Acoraceae): evidences based on phylogenetic and metabolomic analyses. Front. Plant Sci..

[5] Acorus, L. *Plants of World Online*. https://powo.science.kew.org/taxon/urn:lsid:ipni.org:names:2667-1#children (2022).

[6] Wang H, Li WL, Gu ZJ, Chen YY (2001). Cytological study on *Acorus* L. in Southwestern China, with some cytogoegraphical notes on *A. calamus*. J. Integr. Plant Biol..

[7] Morin, N. R. (Ed.). *Flora of North America: North of Mexico Volume 22: Magnoliophyta: Alismatidae, Arecidae, Commelinidae (in Part), and Zingiberidae*, 151 (OUP USA, 1993).

[8] Ranallo-Benavidez TR, Jaron KS, Schatz MC (2020). GenomeScope 2.0 and Smudgeplot for reference-free profiling of polyploid genomes. Nat. Commun..

[9] Simão FA, Waterhouse RM, Ioannidis P, Kriventseva EV, Zdobnov EM (2015). BUSCO: assessing genome assembly and annotation completeness with single-copy orthologs. Bioinformatics.

[10] Mitros T (2020). Genome biology of the paleotetraploid perennial biomass crop *Miscanthus*. Nat. Commun..

[11] Jia KH (2022). SubPhaser: a robust allopolyploid subgenome phasing method based on subgenome‐specific *k*‐mers. N. Phytol..

[12] Bao W, Kojima KK, Kohany O (2015). Repbase Update, a database of repetitive elements in eukaryotic genomes. Mob. DNA.

[13] Su, W., Ou, S., Hufford, M. B., Peterson, T. A tutorial of EDTA: extensive *de novo* TE Annotator. In: Cho, J. (eds) *Plant Transposable Elements. Methods in Molecular Biology*, vol 2250. Humana, New York, NY. (2021). 10.1007/978-1-0716-1134-0_4 33900591

[14] Edger PP, Pires JC (2009). Gene and genome duplications: the impact of dosage-sensitivity on the fate of nuclear genes. Chromosome Res..

[15] Jiao Y, Paterson AH (2014). Polyploidy-associated genome modifications during land plant evolution. Philos. Trans. R. Soc. Lond., B, Biol. Sci..

[16] Blanc G, Wolfe KH (2004). Functional divergence of duplicated genes formed by polyploidy during *Arabidopsis* evolution. Plant Cell.

[17] Aravind L (2000). Lineage-specific loss and divergence of functionally linked genes in eukaryotes. Proc. Natl Acad. Sci. USA.

[18] Xu P (2019). The allotetraploid origin and asymmetrical genome evolution of the common carp *Cyprinus carpio*. Nat. Commun..

[19] Wu HJ, Ma YK, Chen T, Wang M, Wang XJ (2012). PsRobot: a web-based plant small RNA meta-analysis toolbox. Nucleic Acids Res..

[20] Fu L (2022). Microtubules promote the non-cell autonomous action of microRNAs by inhibiting their cytoplasmic loading onto ARGONAUTE1 in Arabidopsis. Dev. Cell.

[21] De Bie T, Cristianini N, Demuth JP, Hahn MW (2006). CAFE: a computational tool for the study of gene family evolution. Bioinformatics.

[22] Comai L (2005). The advantages and disadvantages of being polyploid. Nat. Rev. Genet..

[23] Luttgeharm, K. D., Kimberlin, A. N., Cahoon, E. B. Plant sphingolipid metabolism and function. In: *Lipids in Pant and Algae Development*. Eds: Nakamura, Y., and Li-Beisson, Y. pp. 249–286 (Springer, 2016). 10.1007/978-3-319-25979-6_11 27023239

[24] Sandermann H (1992). Plant metabolism of xenobiotics. Trends Biochem. Sci..

[25] Paterson AH, Bowers JE, Chapman BA (2004). Ancient polyploidization predating divergence of the cereals, and its consequences for comparative genomics. Proc. Natl Acad. Sci. USA.

[26] Yu J (2005). The genomes of *Oryza sativa*: a history of duplications. PLoS Biol..

[27] D’Hont A (2012). The banana (*Musa acuminata*) genome and the evolution of monocotyledonous plants. Nature.

[28] Jiao Y, Li J, Tang H, Paterson AH (2014). Integrated syntenic and phylogenomic analyses reveal an ancient genome duplication in monocots. Plant Cell.

[29] Ming R (2015). The pineapple genome and the evolution of CAM photosynthesis. Nat. Genet..

[30] Wang W (2014). The *Spirodela polyrhiza* genome reveals insights into its neotenous reduction fast growth and aquatic lifestyle. Nat. Commun..

[31] Van de Peer Y, Mizrachi E, Marchal K (2017). The evolutionary significance of polyploidy. Nat. Rev. Genet..

[32] Zhang GQ (2017). The *Apostasia* genome and the evolution of orchids. Nature.

[33] Zhang Q, Luo F, Zhong Y, He J, Li L (2019). Modulation of NAC transcription factor NST1 activity by XYLEM NAC DOMAIN1 regulates secondary cell wall formation in Arabidopsis. J. Exp. Bot..

[34] Paterson AH (2009). The *Sorghum bicolor* genome and the diversification of grasses. Nature.

[35] Tang H, Bowers JE, Wang X, Paterson AH (2010). Angiosperm genome comparisons reveal early polyploidy in the monocot lineage. Proc. Natl Acad. Sci. USA.

[36] Wang X (2015). Genome alignment spanning major Poaceae lineages reveals heterogeneous evolutionary rates and alters inferred dates for key evolutionary events. Mol. Plant.

[37] Harkess A (2017). The *Asparagus* genome sheds light on the origin and evolution of a young Y chromosome. Nat. Commun..

[38] Barrett CF (2019). Ancient polyploidy and genome evolution in palms. Genome Biol. Evol..

[39] Mckain MR (2016). A phylogenomic assessment of ancient polyploidy and genome evolution across the Poales. Genome Biol. Evol..

[40] Michael TP (2017). Comprehensive definition of genome features in *Spirodela polyrhiza* by high-depth physical mapping and short-read DNA sequencing strategies. Plant J..

[41] Al-Mssallem IS (2013). Genome sequence of the date palm *Phoenix dactylifera* L. Nat. Commun..

[42] Wang Y (2012). MCScanX: a toolkit for detection and evolutionary analysis of gene synteny and collinearity. Nucleic Acids Res..

[43] Cheng CY (2017). Araport11: a complete reannotation of the *Arabidopsis thaliana* reference genome. Plant J..

[44] Xu Q (2013). The draft genome of sweet orange (*Citrus sinensis*). Nat. Genet..

[45] Jaillon O (2007). The grapevine genome sequence suggests ancestral hexaploidization in major angiosperm phyla. Nature.

[46] McCormick RF (2018). The *Sorghum bicolor* reference genome: improved assembly, gene annotations, a transcriptome atlas, and signatures of genome organization. Plant J..

[47] Sasaki T, International Rice Genome Sequencing Project (2005). The map-based sequence of the rice genome. Nature.

[48] Bredeson JV (2022). Chromosome evolution and the genetic basis of agronomically important traits in greater yam. Nat. Commun..

[49] Singh R (2013). Oil palm genome sequence reveals divergence of interfertile species in Old and New worlds. Nature.

[50] Murat F, Armero A, Pont C, Klopp C, Salse J (2017). Reconstructing the genome of the most recent common ancestor of flowering plants. Nat. Genet..

[51] Ye CY (2020). The genomes of the allohexaploid *Echinochloa crus-galli* and its progenitors provide insights into polyploidization-driven adaptation. Mol. Plant.

[52] Edger PP, McKain MR, Bird KA, VanBuren R (2018). Subgenome assignment in allopolyploids: challenges and future directions. Curr. Opin. Plant Biol..

[53] Cheng F (2018). Gene retention, fractionation and subgenome differences in polyploid plants. Nat. Plants.

[54] Bird KA, VanBuren R, Puzey JR, Edger PP (2018). The causes and consequences of subgenome dominance in hybrids and recent polyploids. N. Phytol..

[55] Alger EI, Edger PP (2020). One subgenome to rule them all: underlying mechanisms of subgenome dominance. Curr. Opin. Plant Biol..

[56] Takuno S, Gaut BS (2013). Gene body methylation is conserved between plant orthologs and is of evolutionary consequence. Proc. Natl Acad. Sci. USA.

[57] Niederhuth CE (2016). Widespread natural variation of DNA methylation within angiosperms. Genome Biol..

[58] Chen F, Zhang X, Liu X, Zhang L (2017). Evolutionary analysis of MIKCc-type MADS-box genes in gymnosperms and angiosperms. Front. Plant Sci..

[59] Arora R (2007). MADS-box gene family in rice: genome-wide identification, organization and expression profiling during reproductive development and stress. BMC Genomics.

[60] Cai J (2015). The genome sequence of the orchid *Phalaenopsis equestris*. Nat. Genet..

[61] Par̆enicová L (2003). Molecular and phylogenetic analyses of the complete MADS-box transcription factor family in *Arabidopsis*: new openings to the MADS world. Plant Cell.

[62] Sang X (2012). *CHIMERIC FLORAL ORGANS1*, encoding a monocot-specific MADS box protein, regulates floral organ identity in rice. Plant Physiol..

[63] Colombo M (2008). *AGL23*, a type I MADS-box gene that controls female gametophyte and embryo development in *Arabidopsis*. Plant J..

[64] Portereiko MF (2006). *AGL80* is required for central cell and endosperm development in *Arabidopsis*. Plant Cell.

[65] Steffen JG, Kang IH, Portereiko MF, Lloyd A, Drews GN (2008). *AGL61* interacts with *AGL80* and is required for central cell development in Arabidopsis. Plant Physiol..

[66] Ruelens P (2013). FLOWERING LOCUS C in monocots and the tandem origin of angiosperm-specific MADS-box genes. Nat. Commun..

[67] Li MH (2022). Genomes of leafy and leafless *Platanthera* orchids illuminate the evolution of mycoheterotrophy. Nat. Plants.

[68] Liu ZJ, Lan S (2022). The evolutionary mechanisms of mycoheterotrophic orchids. Nat. Plants.

[69] Zhang L (2019). The water lily genome and the early evolution of flowering plants. Nature.

[70] Roodt D, Li Z, Van de Peer Y, Mizrachi E (2019). Loss of wood formation genes in monocot genomes. Genome Biol. Evol..

[71] Etchells JP, Provost CM, Mishra L, Turner SR (2013). *WOX4* and *WOX14* act downstream of the PXY receptor kinase to regulate plant vascular proliferation independently of any role in vascular organisation. Development.

[72] Yanagisawa S (2002). The Dof family of plant transcription factors. Trends Plant Sci..

[73] Smetana O (2019). High levels of auxin signalling define the stem-cell organizer of the vascular cambium. Nature.

[74] Skirycz A (2008). The DOF transcription factor OBP1 is involved in cell cycle regulation in *Arabidopsis thaliana*. Plant J..

[75] Kaneda M, Rensing K, Samuels L (2010). Secondary cell wall deposition in developing secondary xylem of poplar. J. Integr. Plant Biol..

[76] Oda Y, Fukuda H (2012). Secondary cell wall patterning during xylem differentiation. Curr. Opin. Plant Biol..

[77] Jouannet V, Brackmann K, Greb T (2015). (Pro)cambium formation and proliferation: two sides of the same coin?. Curr. Opin. Plant Biol..

[78] Pesquet E, Korolev AV, Calder G, Lloyd CW (2010). The microtubule-associated protein AtMAP70-5 regulates secondary wall patterning in *Arabidopsis* wood cells. Curr. Biol..

[79] Mitsuda N (2007). NAC Transcription factors, NST1 and NST3, are key regulators of the formation of secondary walls in woody tissues of *Arabidopsis*. Plant Cell.

[80] Treml BS (2005). The gene *ENHANCER OF PINOID* controls cotyledon development in the *Arabidopsis* embryo. Development.

[81] Raman S (2008). Interplay of miR164, CUP‐SHAPED COTYLEDON genes and LATERAL SUPPRESSOR controls axillary meristem formation in Arabidopsis thaliana. Plant J..

[82] Aida M, Ishida T, Tasaka M (1999). Shoot apical meristem and cotyledon formation during *Arabidopsis* embryogenesis: interaction among the *CUP-SHAPED COTYLEDON* and *SHOOT MERISTEMLESS* genes. Development.

[83] Furutani M (2004). *PIN-FORMED1* and *PINOID* regulate boundary formation and cotyledon development in *Arabidopsis* embryogenesis. Development.

[84] Yang J, Wang H, Yan G, Qin Y (2000). Callus induction and differentiation from the cotyledon of *Capsicum annuum* L. J. Jilin Agric. Uni..

[85] Baggs EL (2020). Convergent loss of an EDS1/PAD4 signaling pathway in several plant lineages reveals coevolved components of plant immunity and drought response. Plant Cell.

[86] Berbee ML, James TY, Strullu-Derrien C (2017). Early diverging fungi: diversity and impact at the dawn of terrestrial life. Annu. Rev. Microbiol..

[87] Miguel MA, Gabaldón T (2019). Fungal evolution: major ecological adaptations and evolutionary transitions. Biol. Rev..

[88] Zhang C (2021). Discriminating symbiosis and immunity signals by receptor competition in rice. Proc. Natl Acad. Sci. USA.

[89] Motley TJ (1994). The ethnobotany of sweet flag, *Acorus calamus* (Araceae). Econ. Bot..

[90] Mani PG, Audipudi AV (2016). *Penicillium citrinum* AVGE1 an endophyte of *Acorus calamus* its role in biocontrol and PGP in chilli seedlings. Int. J. Curr. Microbiol. Appl. Sci..

[91] Abe S, Sado A, Tanaka K, Nomura T (2014). Carlactone is converted to carlactonoic acid by MAX1 in *Arabidopsis* and its methyl ester can directly interact with AtD14 in vitro. Proc. Natl Acad. Sci. USA.

[92] Crawford S (2010). Strigolactones enhance competition between shoot branches by dampening auxin transport. Development.

[93] Lanfranco L, Fiorilli V, Venice F, Bonfante P (2018). Strigolactones cross the kingdoms: plants, fungi, and bacteria in the arbuscular mycorrhizal symbiosis. J. Exp. Bot..

[94] Lander ES, Waterman MS (1988). Genomic mapping by fingerprinting random clones: a mathematical analysis. Genomics.

[95] Marçais G, Carl K (2011). A fast, lock-free approach for efficient parallel counting of occurrences of *k*-mers. Bioinformatics.

[96] Vurture GW (2017). GenomeScope: fast reference-free genome profiling from short reads. Bioinformatics.

[97] Jue, R. *Smartdenovo: Ultra-Fast De Novo Assembler Using Long Noisy Reads*. https://github.com/ruanjue/smartdenovo (2016). 10.46471/gigabyte.15 PMC9632051 36824332

[98] Chin CS (2016). Phased diploid genome assembly with single-molecule real-time sequencing. Nat. Methods.

[99] Walker BJ (2014). Pilon: an integrated tool for comprehensive microbial variant detection and genome assembly improvement. PLoS ONE.

[100] Burton JN (2013). Chromosome-scale scaffolding of de novo genome assemblies based on chromatin interactions. Nat. Biotechnol..

[101] Li H, Durbin R (2009). Fast and accurate short read alignment with Burrows– Wheeler transform. Bioinformatics.

[102] Birney E, Clamp M, Durbin R (2004). GeneWise and Genomewise. Genome Res..

[103] Stanke M, Waack S (2003). Gene prediction with a hidden Markov model and a new intron submodel. Bioinformatics.

[104] Majoros WH, Pertea M, Salzberg SL (2004). TigrScan and GlimmerHMM: two open source ab initio eukaryotic gene-finders. Bioinformatics.

[105] Korf I (2004). Gene finding in novel genomes. BMC Bioinform.

[106] Trapnell C (2012). Differential gene and transcript expression analysis of RNA-seq experiments with TopHat and Cufflinks. Nat. Protoc..

[107] Holt C, Yandell M (2011). MAKER2: an annotation pipeline and genome- database management tool for second-generation genome projects. BMC Bioinform.

[108] Finn RD (2017). InterPro in 2017—beyond protein family and domain annotations. Nucleic Acids Res.

[109] Lowe TM, Eddy SR (1997). tRNAscan-SE: a program for improved detection of transfer RNA genes in genomic sequence. Nucleic Acids Res.

[110] Nawrocki EP, Kolbe DL, Eddy SR (2009). Infernal 1.0: inference of RNA alignments. Bioinformatics.

[111] Smit, A., Hubley, R. & Green, P. *RepeatMasker Open-4.0* http://www.repeatmasker.org/RMDownload.html (2013).

[112] Xu Z, Wang H (2007). LTR_FINDER: an efficient tool for the prediction of full-length LTR retrotransposons. Nucleic Acids Res.

[113] Edgar RC, Myers EW (2005). PILER: identification and classification of genomic repeats. Bioinformatics.

[114] Smit, A. & Hubley, R. *RepeatModeler Open-1.0.* http://www.repeatmasker.org/RepeatModeler (2008).

[115] Li L, Stoeckert CJ, Roos DS (2003). OrthoMCL: identification of ortholog groups for eukaryotic genomes. Genome Res.

[116] Chen F (2006). OrthoMCL-DB: querying a comprehensive multi-species collection of ortholog groups. Nucleic Acids Res..

[117] Vanneste K, Baele G, Maere S, Van de Peer Y (2014). Analysis of 41 plant genomes supports a wave of successful genome duplications in association with the Cretaceous-Paleogene boundary. Genome Res.

[118] Proost S (2012). i-ADHoRe 3.0—fast and sensitive detection of genomic homology in extremely large data sets. Nucleic Acids Res.

[119] Fostier J (2011). A greedy, graph-based algorithm for the alignment of multiple homologous gene lists. Bioinformatics.

[120] Edgar RC (2004). MUSCLE: multiple sequence alignment with high accuracy and high throughput. Nucleic Acids Res.

[121] Talavera G, Castresana J (2007). Improvement of phylogenies after removing divergent and ambiguously aligned blocks from protein sequence alignments. Syst. Biol..

[122] Huelsenbeck JP, Ronquist F (2001). MRBAYES: Bayesian inference of phylogenetic trees. Bioinformatics.

[123] Yang Z (1994). Maximum likelihood phylogenetic estimation from DNA sequences with variable rates over sites: approximate methods. J. Mol. Evol..

[124] Yang Z (2007). PAML 4: phylogenetic analysis by maximum likelihood. Mol. Biol. Evol..

[125] Yang Y (2020). Prickly waterlily and rigid hornwort genomes shed light on early angiosperm evolution. Nat. Plants.

[126] Guo X (2021). *Chloranthus* genome provides insights into the early diversification of angiosperms. Nat. Commun..

[127] Zhang J (2020). The hornwort genome and early land plant evolution. Nat. Plants.

[128] Niu S (2022). The Chinese pine genome and methylome unveil key features of conifer evolution. Cell.

[129] Yang Z, Rannala B (2006). Bayesian estimation of species divergence times under a molecular clock using multiple fossil calibrations with soft bounds. Mol. Biol. Evol..

[130] Chaw SM, Chang CC, Chen HL, Li WH (2004). Dating the monocot–dicot divergence and the origin of core eudicots using whole chloroplast genomes. J. Mol. Evol..

[131] Magallón S, Hilu KW, Quandt D (2013). Land plant evolutionary timeline: gene effects are secondary to fossil constraints in relaxed clock estimation of age and substitution rates. Am. J. Bot..

[132] Zhang T (2015). Sequencing of allotetraploid cotton (*Gossypium hirsutum* L. acc. TM-1) provides a resource for fiber improvement. Nat. Biotechnol..

[133] Kielbasa SM, Wan R, Sato K, Horton P, Frith MC (2011). Adaptive seeds tame genomic sequence comparison. Genome Res.

[134] Zhuang W (2019). The genome of cultivated peanut provides insight into legume karyotypes, polyploid evolution and crop domestication. Nat. Genet..

[135] Qin L (2021). Insights into angiosperm evolution, floral development and chemical biosynthesis from the *Aristolochia fimbriata* genome. Nat. Plants.

[136] Zdobnov EM, Apweiler R (2001). InterProScan-an integration platform for the signature-recognition methods in InterPro. Bioinformatics.

[137] Letunic I, Doerks T, Bork P (2015). SMART: recent updates, new developments and status in 2015. Nucleic Acids Res..

[138] Oliver T, Schmidt B, Nathan D, Clemens R, Maskell D (2005). Using reconfigurable hardware to accelerate multiple sequence alignment with ClustalW. Bioinformatics.

[139] Tamura K (2011). MEGA5: molecular evolutionary genetics analysis using maximum likelihood, evolutionary distance, and maximum parsimony methods. Mol. Biol. Evol..

[140] Haas BJ (2013). De novo transcript sequence reconstruction from RNA-seq using the Trinity platform for reference generation and analysis. Nat. Protoc..

