# Supplementary material for: Diploid and tetraploid genomes of Acorus and the evolution of monocots
Source: Nat Commun. 2023 Jun 20;14:3661. doi: 10.1038/s41467-023-38829-3 (PMC10282084; doi:10.1038/s41467-023-38829-3)
Supplement: Supplementary file 1 — Supplementary Information [file 41467_2023_38829_MOESM1_ESM.pdf]

Diploid and tetraploid genomes of *Acorus* and the evolution of monocots

Ma *et al.*

Supplementary Note 1. *K*-mer, heterozygous, homozygous, homoeologous and polyploid model analysis

K-mer analysis for *Ac. gramineus* and *Ac. calamus* genomes were performed using Smudgeplot and GenomeScope2[1], which showed that *Ac. gramineus* had AB type *K*-mer pair with a proportion up to 60%, suggesting that *Ac. gramineus* was a diploid; *Ac. calamus* had AABB type *K*-mer pair with the proportion of AABB type *K*-mer pairs was higher (43%) than that of AABB type (23%), suggesting that *Ac. calamus* genome was an allotetraploid. We used *K*-mer analysis to estimate the genome characteristics[1]. Briefly, *K*-mer of all reads of an unknown genome is obtained by the next-generation sequencing, together with the counted frequency of each *K*-mer to obtain the *K*-mer profile of the sequencing data. The *K*-mer frequency distribution reflected the characteristics of the genome (such as genome size, heterozygosity, and duplication).

K-mer is a sequence with a length of k bases selected iteratively from the sequence, if the read length is L and the *K*-mer length is k, then a read will yield L-k+1 *K*-mer. *K*-mer spectrum is the *K*-mer frequency distribution of sequencing data, and the shape of the *K*-mer spectrum reflects the complexity of the genome. For an ideal genome sequencing data, where the genome is free of heterozygosity and duplication and the sequencing is free of errors and biases, the *K*-mer spectrum obeys the Poisson distribution with a mean *K*-mer coverage depth. Therefore, ideally, the average depth of coverage of C_{kmer} can be obtained from the *K*-mer spectrum to estimate the genome size as following:

$$G = \frac{N_{kmer}}{C_{kmer}} = \frac{N_{read} \times (L - k + 1)}{C_{kmer}} \qquad (1)$$

N_{kmer} and N_{read} are the total number of *K*-mer and read, respectively, C_{kmer} is the average coverage depth of *K*-mer, and L and k are read length and *K*-mer length, respectively.

For actual sequencing data, it tends to be overly discrete due to the presence of PCR duplications, sequencing errors and coverage bias. Therefore, the use of a negative binomial distribution model is more representative of the actual sequencing data than the Poisson distribution. And there are also heterozygosity and duplication in the genomes actually sequenced. Assuming that a genome has no repeats and only heterozygosity, there are two peaks in its *K*-mer

spectrum with the centers of the peaks located at $C_{kmer}/2$ and C_{kmer}, respectively, because the genomic heterozygosity site generates two different *K*-mer, and its sequencing depth is half that of the *K*-mer generated at the pure site. If the genome is a diploid heterozygous repeat genome, the *K*-mer number is doubled due to the genome duplication, then four peaks appear in the *K*-mer spectrum with the centers located in $C_{kmer}/2$, C_{kmer}, $3C_{kmer}/2$ and $2C_{kmer}$. In summary, the *genomeScope1.0*[2] software therefore uses a hybrid model consisting of four negative binomial distributions to describe the *K*-mer spectrum from diploid genome. For the polyploid genome, the GenomeScope 2.0 software upgrade the model to fit 2 × p negative binomial distributions, where p is the ploidy. As follow:

$$f(x) = G \sum_{i=1}^{2p} a_i \, NB\left(x; i\lambda, \frac{i\lambda}{\rho}\right) \qquad (2)$$

The model is fitted with the *K*-mer profile of genome sequencing data to estimate the characteristics of genome (genome size, heterozygosity and repeatability) by least square method.

Supplementary Note 2. The conserved single-copy gene families were selected

The single copy genes of 19 species were selected based on:

1. We need to clarify not only the phylogenetic relationship among *Ac. calamus* A, B and *Ac. gramineus*, but also the phylogenetic position of *Ac. calamus* in monocots and the divergence time within monocots. Therefore, several monocots including Poales, Zingiberales, Arecales, Asparagales and Alismatales that constitute the main clades of monocots were selected.

2. Considering *Ac. calamus* is the earliest diverged monocots, to precisely evaluate the phylogenetic position and formation time of monocots, we also selected several representative dicots, as well as the basal angiosperm groups, *N. colorata* and *A. trichopoda* as outgroups.

3. Because single copy gene family is conserved in species, therefore we used them to construct phylogenetic trees, which is the common method for phylogenetic analysis.

After WGD, genomes usually experienced a large number of gene loss and divergence, although WGD could increase the copy number of some single copy genes, making them no longer single-

copy. However, we have identified 379 single copy genes in 19 species, which may be sufficient for reflecting their phylogenetic relationship.

Supplementary Note 3. Subgenome dominance

Subgenome dominance occurs when one of the subgenomes has more genes with higher expression, experiencing stronger purifying selection or maintaining lower DNA methylation level than those of the other subgenomes[3,4,5]. In *Ac. calamus*, we assigned 21,743 genes in 318.86 Mb sequences to the ten chromosomes of subgenome A and 24,322 genes in 360.79 Mb sequences to the 12 chromosomes of subgenome B. The gene content of subgenome B was higher than that of subgenome A, and has more gene loss in subgenome A than that in subgenome B (Supplementary Table 26). However, gene structure and the distribution of repetitive elements did not show significant differences between the two subgenomes (Supplementary Figs. 41, 42). We also compared the expression profiles of homologs of subgenomes A and B in seven tissues (the flower, leaf, stem, root, bract, peduncle and inflorescence base). Gene family clustering results showed that there were 13,754 homoeologous gene pairs between subgenomes A and B, 31.91%–38.79% of which showed expression bias in seven tissues (see Methods; Fig. 4f; Supplementary Figs. 31, 32; Supplementary Table 21). In addition, the number of gene pairs (2,085–2,270) that expressed bias towards subgenome B was significantly higher than the number of gene pairs that expressed bias towards subgenome A (1,498–1,667). By further investigating whether the homoeologous gene pairs maintain the same expression bias in all tissues, we found that the gene pairs (470) of subgenome B showing bias in all tissues was higher than the gene pairs (338) of subgenome A (Supplementary Table 22, Supplementary Data 14), indicating the bias in gene expression of *Ac. calamus* B (Supplementary Table 22).

The OrthoMCL clustering results indicated that if a gene family is single copy in both the *Ac. calamus* B subgenome and the *Ac. gramineus* genome but is absent in the *Ac. calamus* A subgenome, the gene family has been lost in subgenome A. Similarly, single-gene families lost in subgenome B can be single copy in subgenome A and the *Ac. gramineus* genome. We found that 1,546 and 1,051 genes were lost in subgenome A and subgenome B, respectively (Supplementary Data 15). A GO enrichment analysis showed that the genes lost in subgenome A were mainly

enriched in the ‘glucocorticoid receptor activity’ (*P* value<0.01), while the genes lost in subgenome B were primarily enriched in the ‘proton-transporting ATP synthase complex assembly’, ‘pectinesterase activity’ and ‘rRNA modification’ (*P* value<0.01) (Supplementary Tables 27, 28).

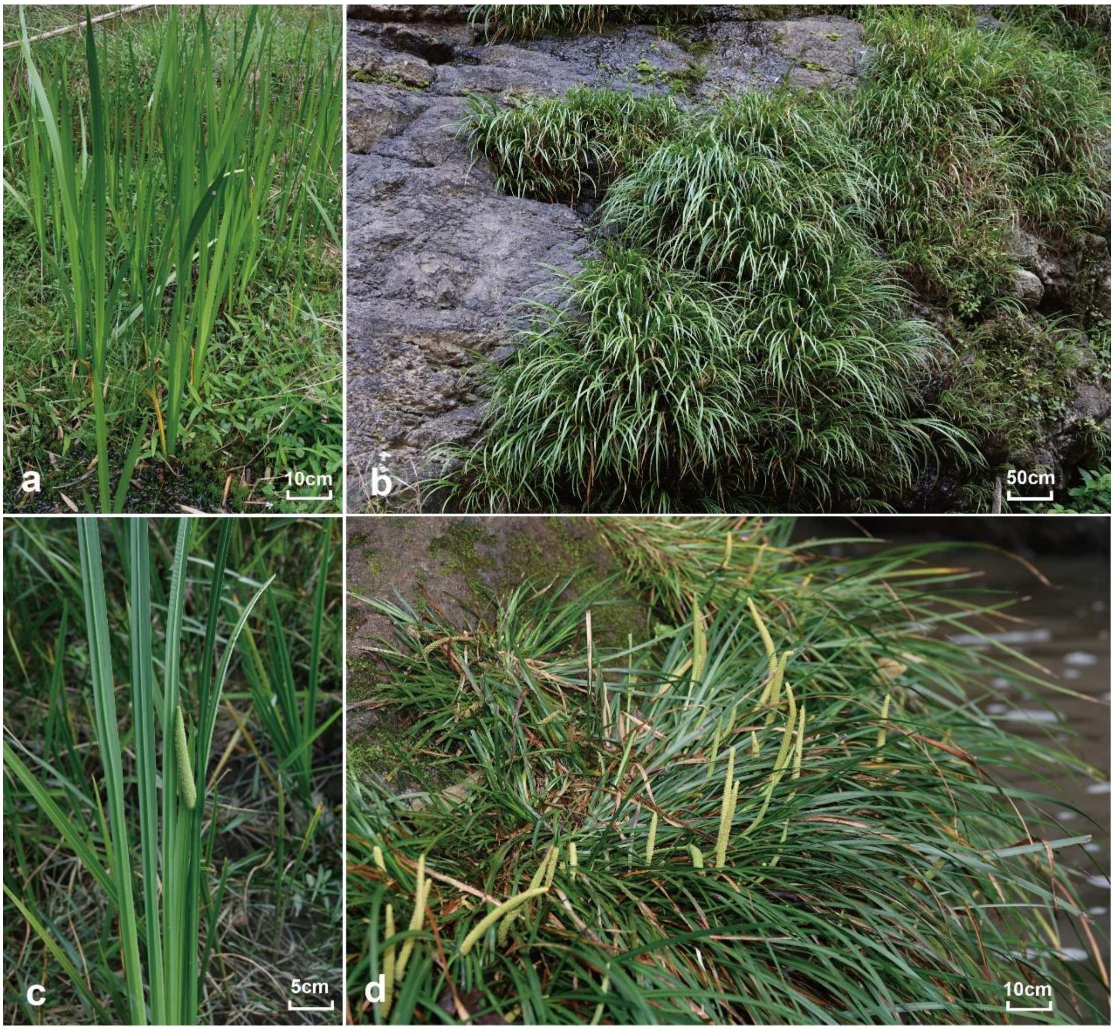

Supplementary Figure 1. The nature habitat and appearance of *Ac. calamus* and *Ac. gramineus*. **a** *Ac. calamus* is a terrestrial plant growing in wet ground. **b** *Ac. gramineus* is epiphytic plant growing on the rock. **c** The flowering plant of *Ac. calamus*. **d** The flowering plant of *Ac. gramineus.*

a

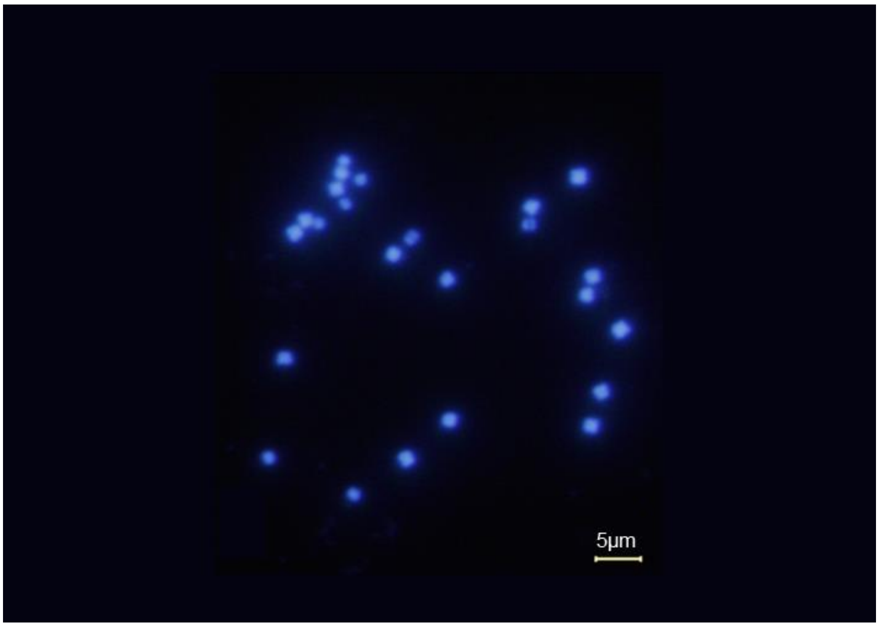

b

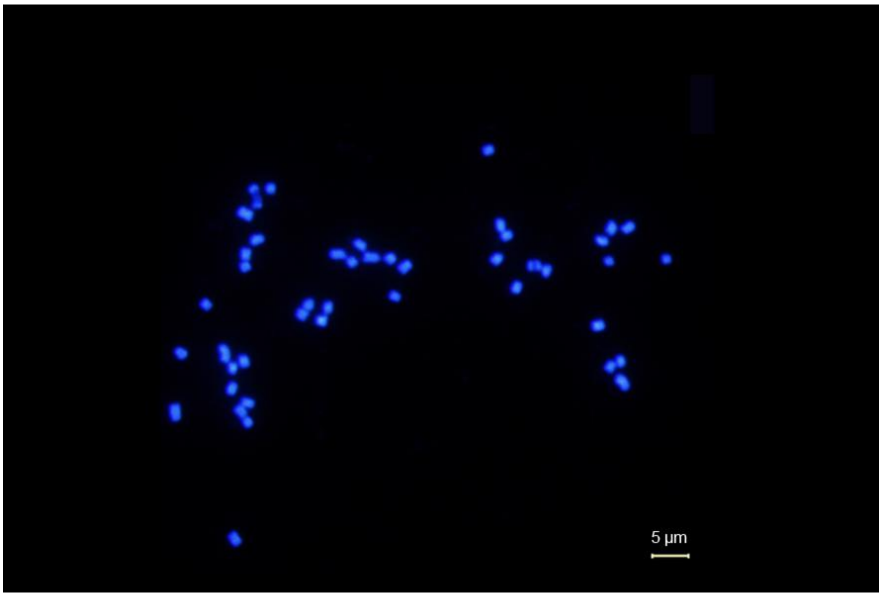

Supplementary Figure 2. Fluorescent dye (DAPI)-stained chromosomal complements with three replicates. a. *Ac. gramineus* presents 24 mitotic metaphase chromosomes in a root cell. **b.** *Ac. calamus* presents 44 mitotic metaphase chromosomes in a root cell.

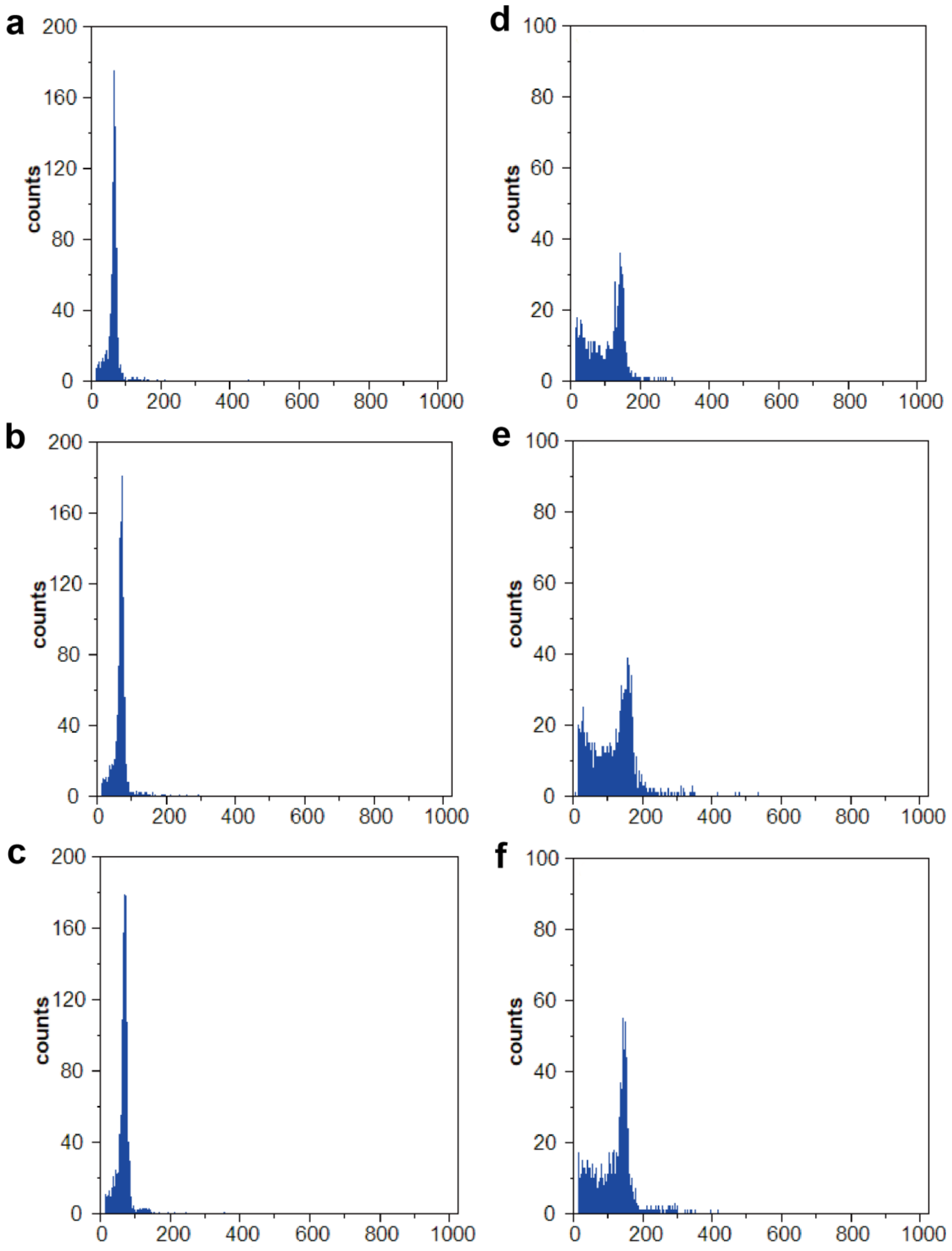

Supplementary Figure 3. The genome size and chromosome ploidy were estimated in *Ac. gramineus* and *Ac. calamus* by flow cytometry with three replicates. The results show that *Ac. gramineus* (a, b, c) is diploid with genome size 362.01 Mb, and *Ac. calamus* (d, e, f) is tetraploid with genome size 747.46 Mb.

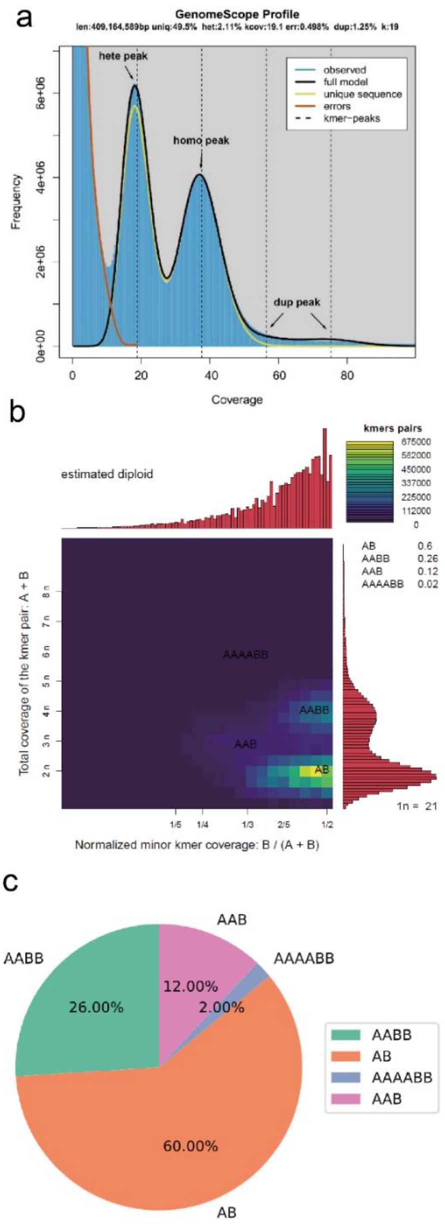

Supplementary Figure 4. *K*-mer distribution of sequencing reads of *Ac. gramineus*. a Diploid GenomeScope plot. The genome size of *Ac. gramineus* was estimated as 409 Mb. Hete peak, the heterozygous peak refers to the peak value of heterozygous *k*-mer profile located at the heterozygous region of genome; homo peak, the homozygous peak refers to the peak value of homozygous *k*-mer profile located at the homozygous region of genome; dup peak, the duplicated peak shows the peak value of duplicated *k*-mer profile located at the duplicated region of genome. AABB type *K*-mer pair was identified in *Ac. gramineus* genome using Smudgeplot. **b** Smudgeplot for the diploid root-knot nematode (Y-axis is total coverage of homologous k-mer pair (CovA+CovB) from allelic or duplicated locus of *Ac. gramineus* genome, X-axis is relative minor k-mer coverage (CovB/CovA+CovB)). The colorbar indicates number of k-mer pair from distinct genomic structure (AB, AAB, AABB or AAAB). **c** The proportion of AABB type *K*-mer pairs. The proportion of AB type *K*-mer pair is up to 60% and *Ac. gramineus* is a diploid. Source data are provided as a Source Data file.

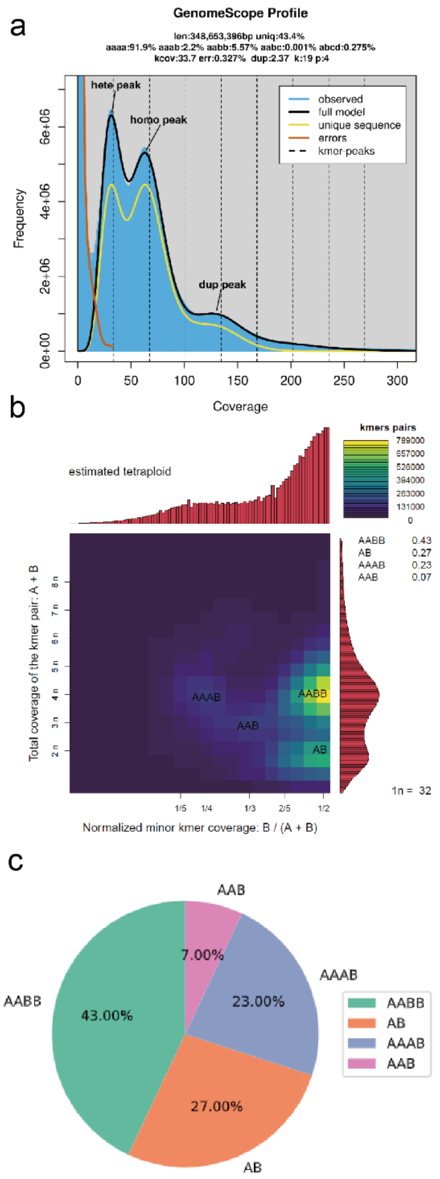

Supplementary Figure 5. *K*-mer distribution of sequencing reads of *Ac. calamus*. a Allotetraploid GenomeScope plot. The average size of two subgenomes of *Ac. calamus* was estimated as 348 Mb. Hete peak, the heterozygous peak refers to the peak value of heterozygous *k*-mer profile located at the heterozygous region of genome; homo peak, the homozygous peak refers to the peak value of homozygous *k*-mer profile located at the homozygous region of genome; dup peak, the duplicated peak shows the peak value of duplicated *k*-mer profile located at the duplicated region of genome. **b** Smudgeplot for the allotetraploid root-knot nematode (Y-axis is total coverage of homologous k-mer pair (CovA+CovB) from allelic or duplicated locus of *Ac. calamus* genome, X-axis is relative minor k-mer coverage (CovB/CovA+CovB)). The colorbar indicates number of k-mer pair from distinct genomic structure (AB, AAB, AABB or AAAB). **c** The proportion of AABB type *K*-mer pair. AABB type *K*-mer pair was identified in *Ac. calamus* genome using Smudgeplot, showing that the proportion of AABB type *K*-mer pair was significantly higher (43%) than AAAB type *K*-mer pair (23%), which further confirmed that *Ac. calamus* genome was an allotetraploid. Source data are provided as a Source Data file.

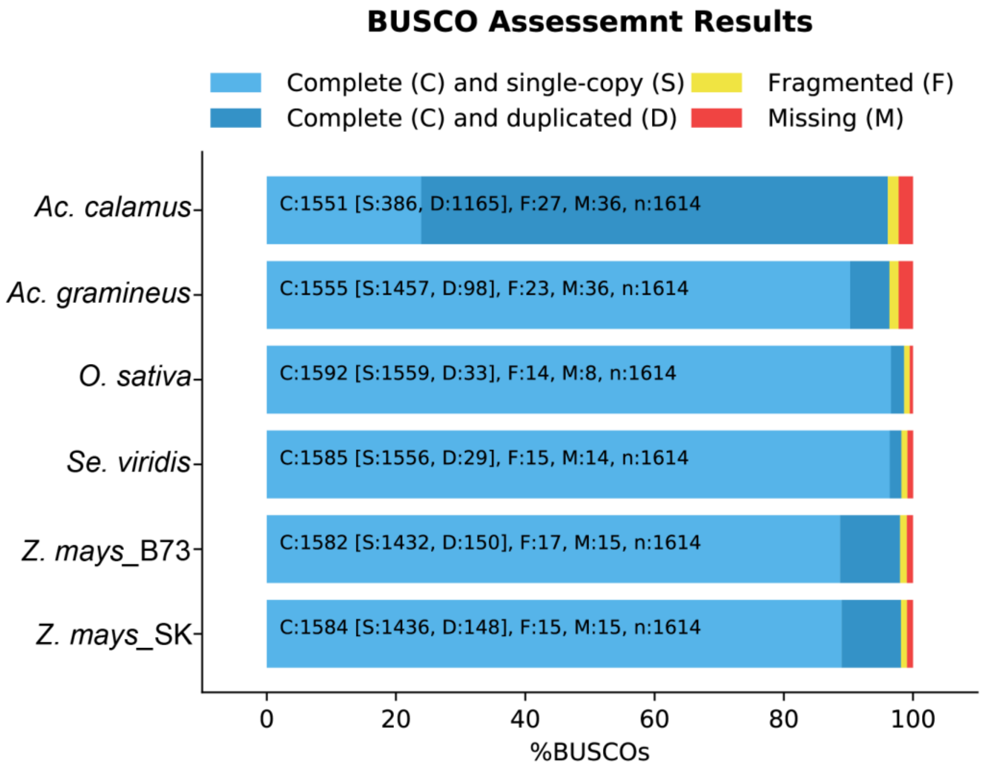

Supplementary Figure 6. Genomic BUSCO assessment of *Ac. calamus* and other monocot species. Source data are provided as a Source Data file.

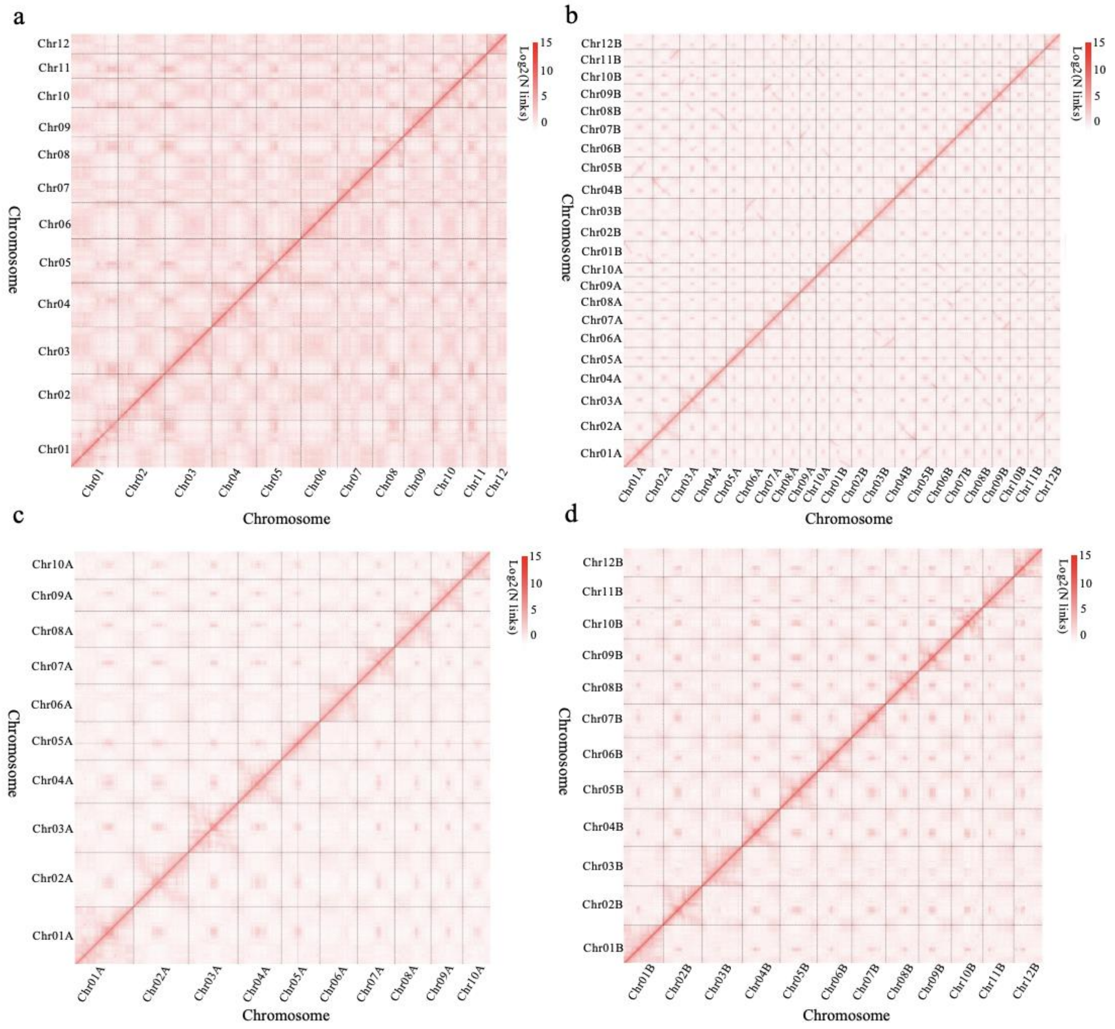

Supplementary Figure 7. The Hi-C interaction matrices for chromosomes in *Acorus*. a *Ac. gramineus*. **b** *Ac. calamus*. **c** Subgenome A of *Ac. calamus***. d** Subgenome B of *Ac. calamus.* The X-axis and Y-axis is chromosome of *Acorus* genome, the colorbar represents log2 normalized number of Hi-C valid reads, indicating the contact intensity of different genomic sites.

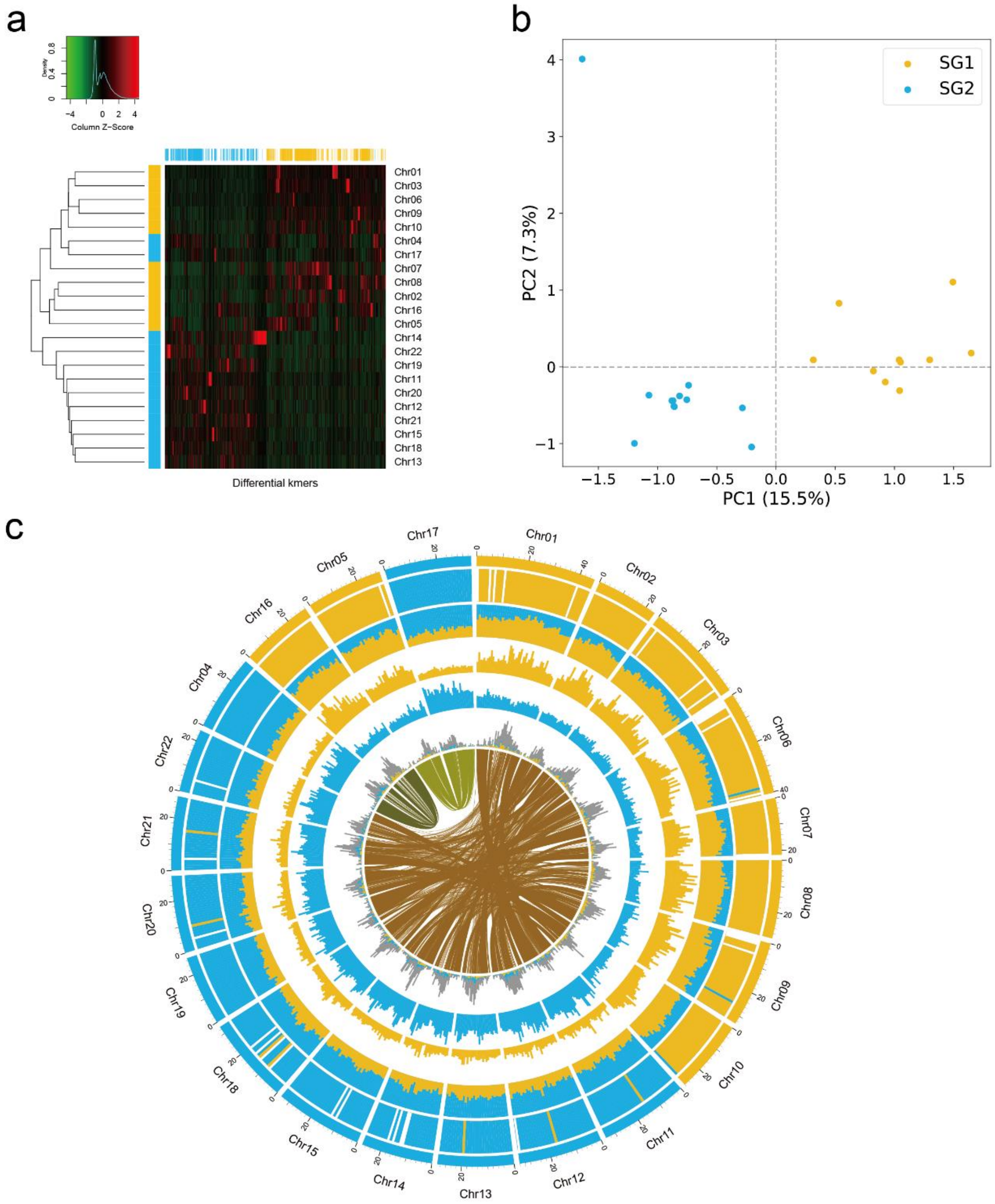

Supplementary Figure 8. Subgenome construction by SubPhaser. a Unsupervised hierarchical clustering (the horizontal color bar at the top of the axis indicates to which subgenome the *k*-mer is specific; the vertical color bar on the left of the axis indicates the subgenome to which the chromosome is assigned with blue for subgenome A and yellow for subgenome B (same colors for subgenomes for the figure panel). The heat map indicates the Z-scaled relative abundance of *k*-mers. The larger the Z score, the higher the relative abundance of a *k*-mer). **b** Principal component analysis (PCA) of differential *k*-mers validates that the genome is successfully phased into two subgenomes based on clearly distinct patterns of both differential *k*-mers and homoeologous chromosomes. **c** Chromosomal characteristics. From outer to inner circles (1–8): (1) subgenome assignments based on *k*-means algorithm; (2) significant enrichment of subgenome-specific *k*-

mers – the same color as the subgenome indicates significant enrichment for those subgenome-specific *k*-mers; white areas are not significantly enriched; (3) normalized proportion (relative) of subgenome-specific *k*-mers; (4–6) count (absolute) of each subgenome- specific *k*-mer set ; (7) density of long terminal repeat retrotransposons (LTR-RTs) – if the color is consistent with the subgenome, it indicates that LTR-RTs are significantly enriched to those subgenome-specific *k*-mers; gray indicates nonspecific LTR-RTs (Blue color: subgenome A, Yellow color: subgenome B); (8) homoeologous blocks. All statistics (2–7) are computed in sliding windows of 1 Mb. All homoeologous exchanges (see circles) were inferred (Light green, dark green and brown colors indicated different homoeologous blocks). Source data are provided as a Source Data file.

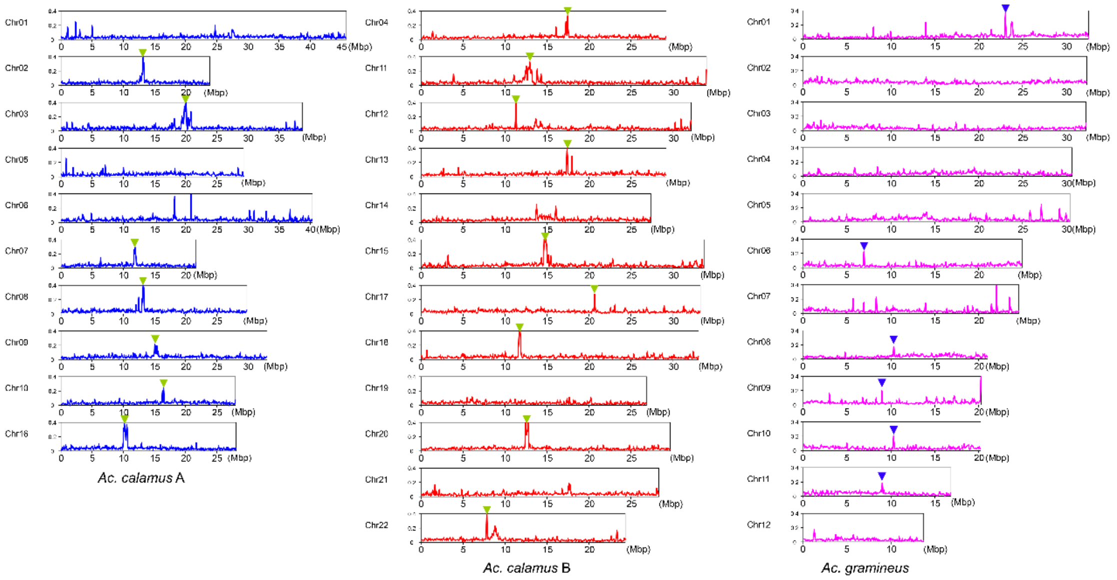

Supplementary Figure 9. Comparison of tandem repeat distribution between *Ac. calamus* A and B, and *Ac. gramineus*. The 100kb windows was used to calculate the ratio of tandem repeat, the regions enrichment of tandem repeat might represent the centromere/telomeric. In *Ac. calamus*, 16 putative centromeres may be detected (chr2,3,7,8,9,10,16,4,11,12,13,15,17,18,20,22), in *Ac. gramineus*, 6 putative centromeres may be detected (chr1,6,8,9,10,11). Source data are provided as a Source Data file.

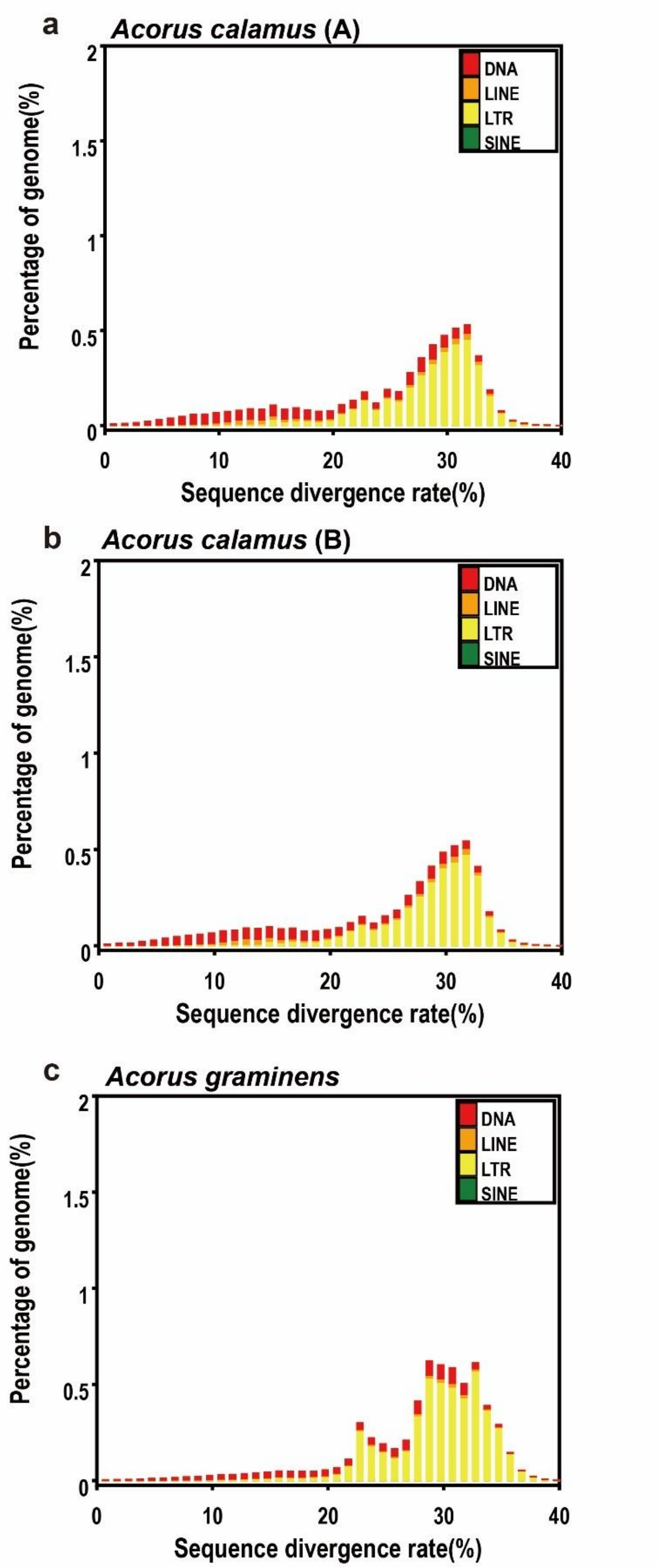

Supplementary Figure 10. The sequence divergence rate of four different TEs using RepeatMasker annotation. a *Ac. gramineus* genome. **b** *Ac. calamu*s subgenome A**. c** *Ac. calamus* subgenome B.

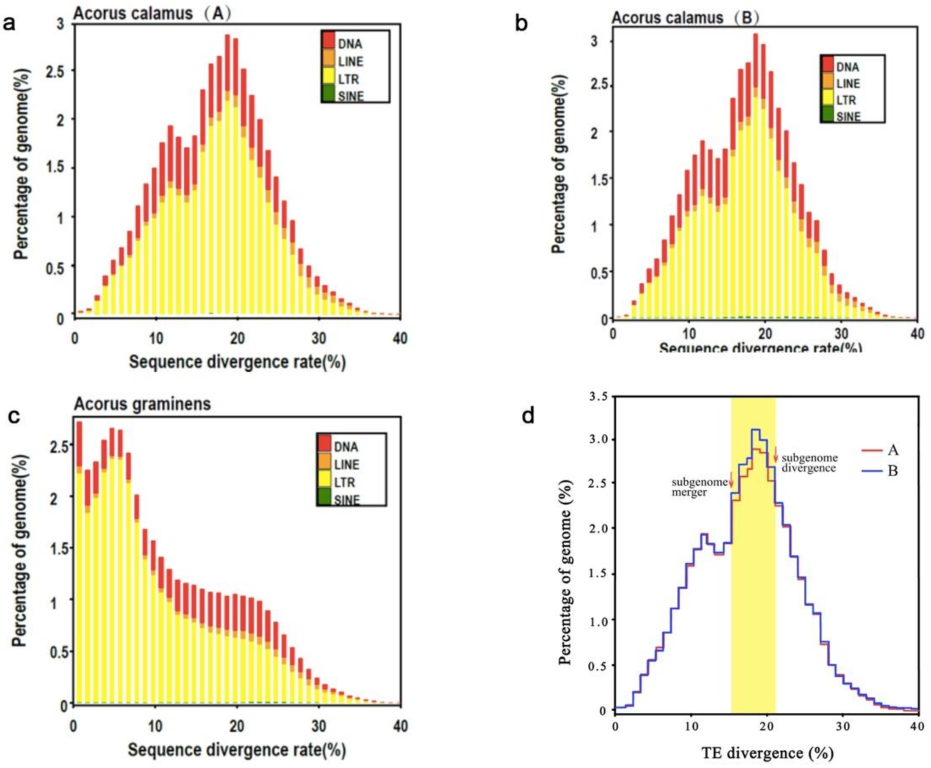

Supplementary Figure 11. The sequence divergence rate of four different TEs using *de novo* annotation. a *Ac. gramineus* genome. **b** *Ac. calamu*s subgenome A. **c** *Ac. calamus* subgenome B. **d** The distribution of sequence divergence rates of transposable elements (TEs) as percentages of subgenome sizes of *Ac. calamus* A and *Ac. calamus* B.

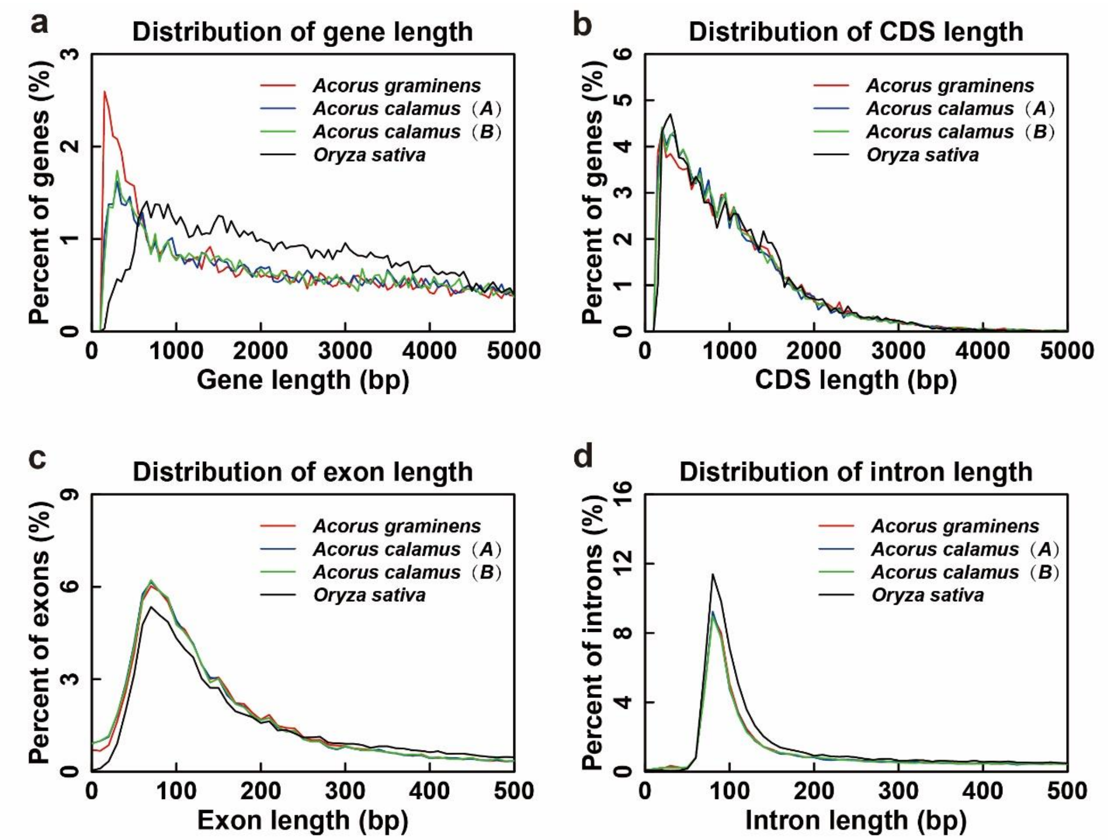

Supplementary Figure 12. Statistics of gene structure prediction. a Gene length. **b** CDS length. **c** Exon length. **d** Intron length. *Ac. gramineus, Ac. calamus* A and *Ac. calamus* B compared with genetic elements of *O. sativa*. Window refers to the length represented by each point on the horizonal coordinate. Source data are provided as a Source Data file.

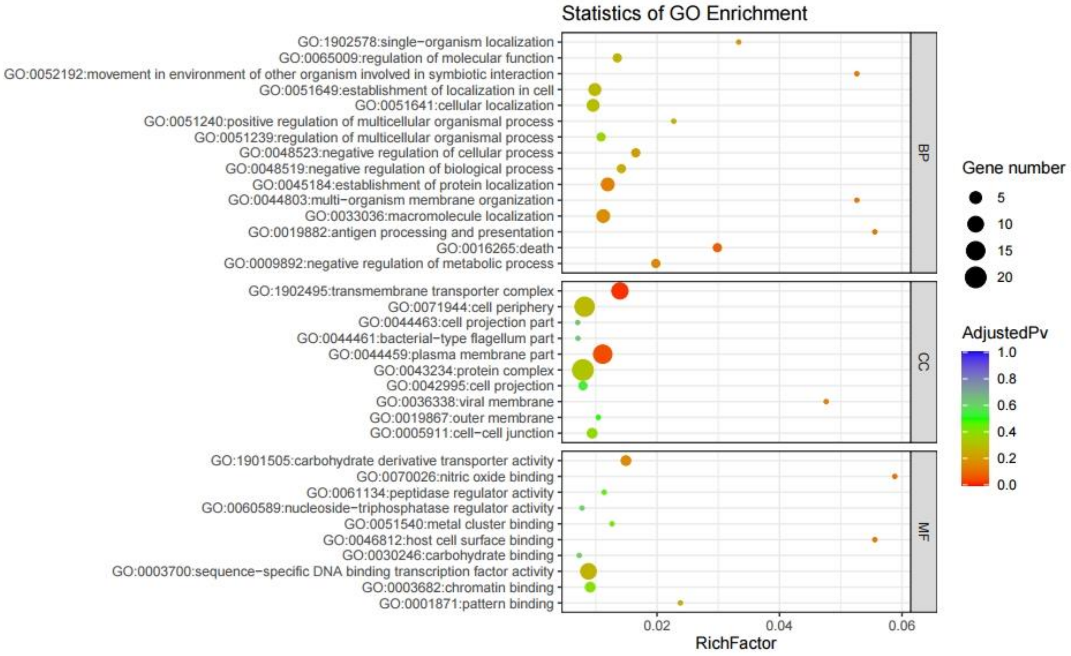

Supplementary Figure 13. GO enrichment terms for microRNA target genes of *Ac. gramineus.* BP, MF, and CC represent Biological Process, Molecular Function, and Cellular Component groups of GO, respectively. The size of the circle represents the number of genes, and the heatmap color bar represents the adjust *p*-value of enrichment.

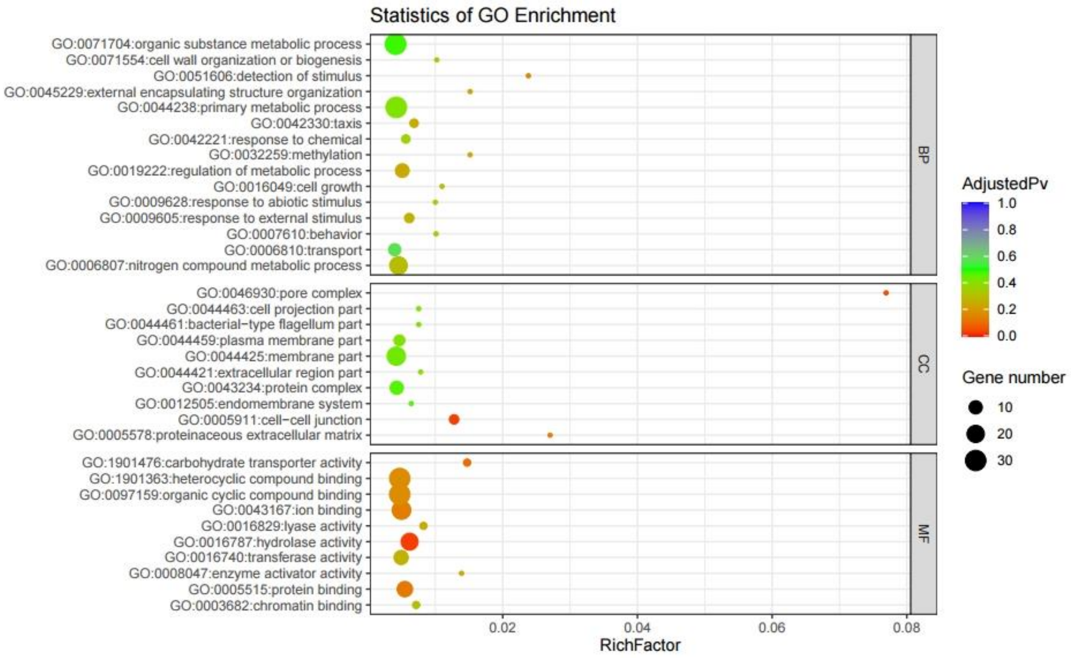

Supplementary Figure 14. GO enrichment terms for microRNA target genes of *Ac. calamus* A. BP, MF, and CC represent Biological Process, Molecular Function, and Cellular Component groups of GO, respectively. The size of the circle represents the number of genes, and the heatmap color bar represents the adjust *p*-value of enrichment.

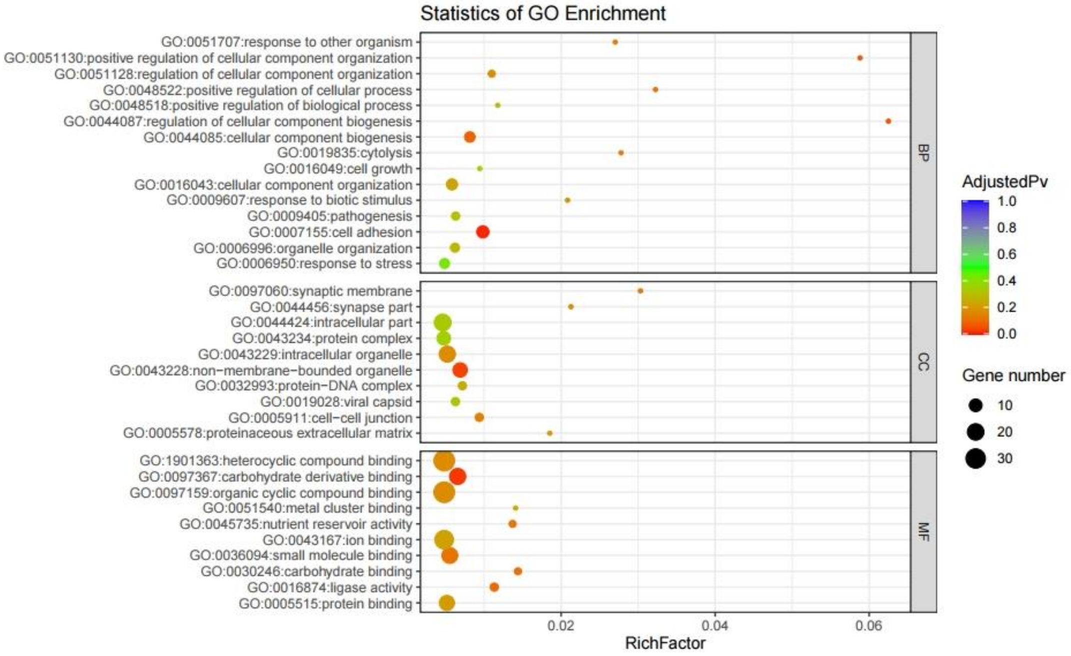

Supplementary Figure 15. GO enrichment terms for microRNA target genes of *Ac. calamus* B. BP, MF, and CC represent Biological Process, Molecular Function, and Cellular Component groups of GO, respectively. The size of the circle represents the number of genes, and the heatmap color bar represents the adjust *p*-value of enrichment.

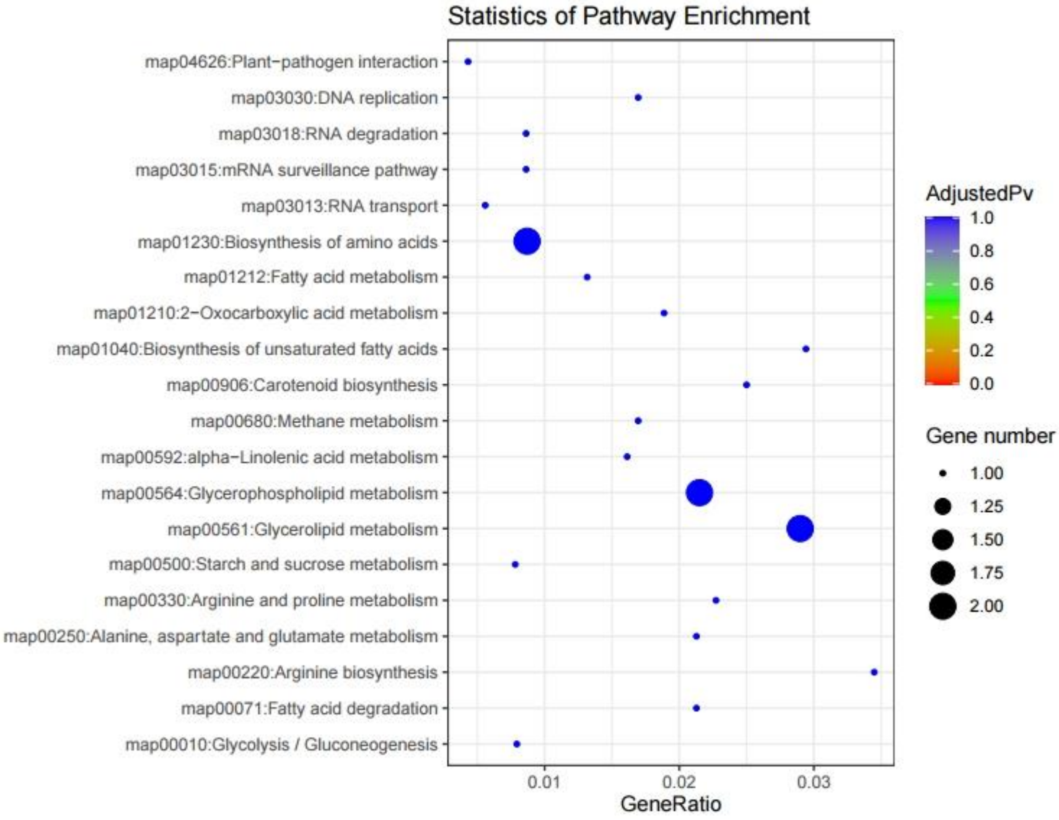

Supplementary Figure 16. KEGG enrichment pathways for microRNA target genes of *Ac. gramineus*. The size of the circle represents the number of genes, and the heatmap color bar represents the adjust *p*-value of enrichment.

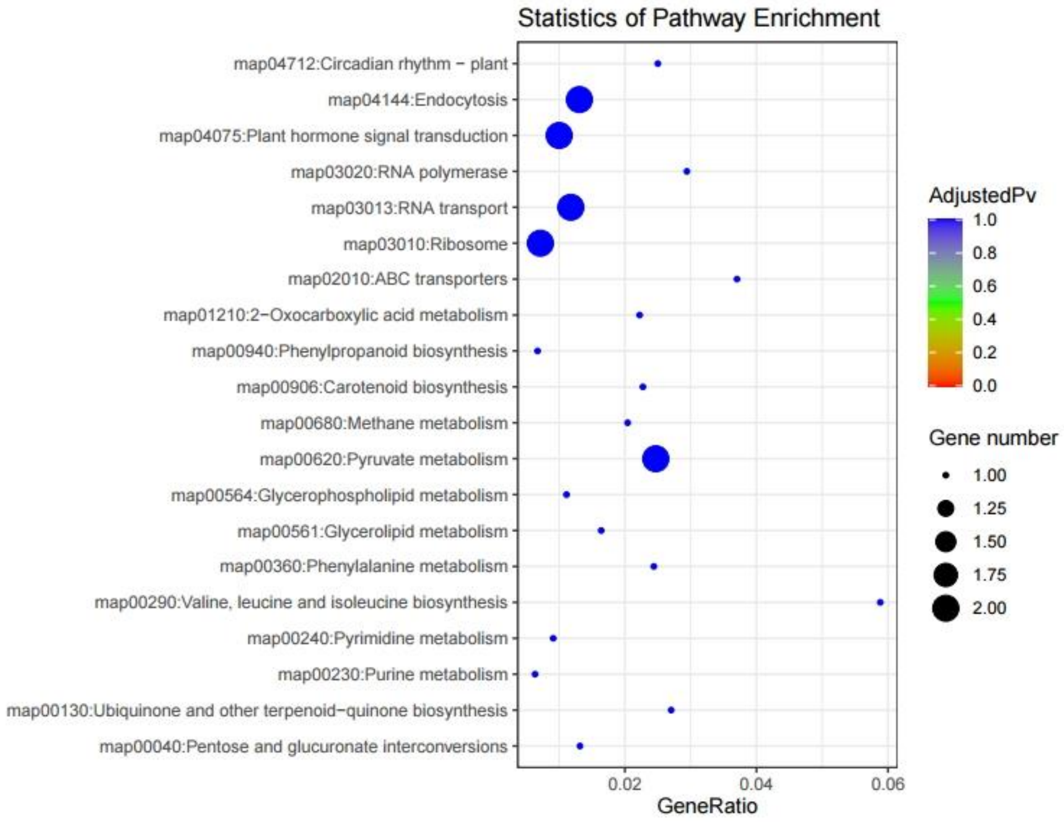

Supplementary Figure 17. KEGG enrichment pathways for microRNA target genes of *Ac. calamus* A. The size of the circle represents the number of genes, and the heatmap color bar represents the adjust *p*-value of enrichment.

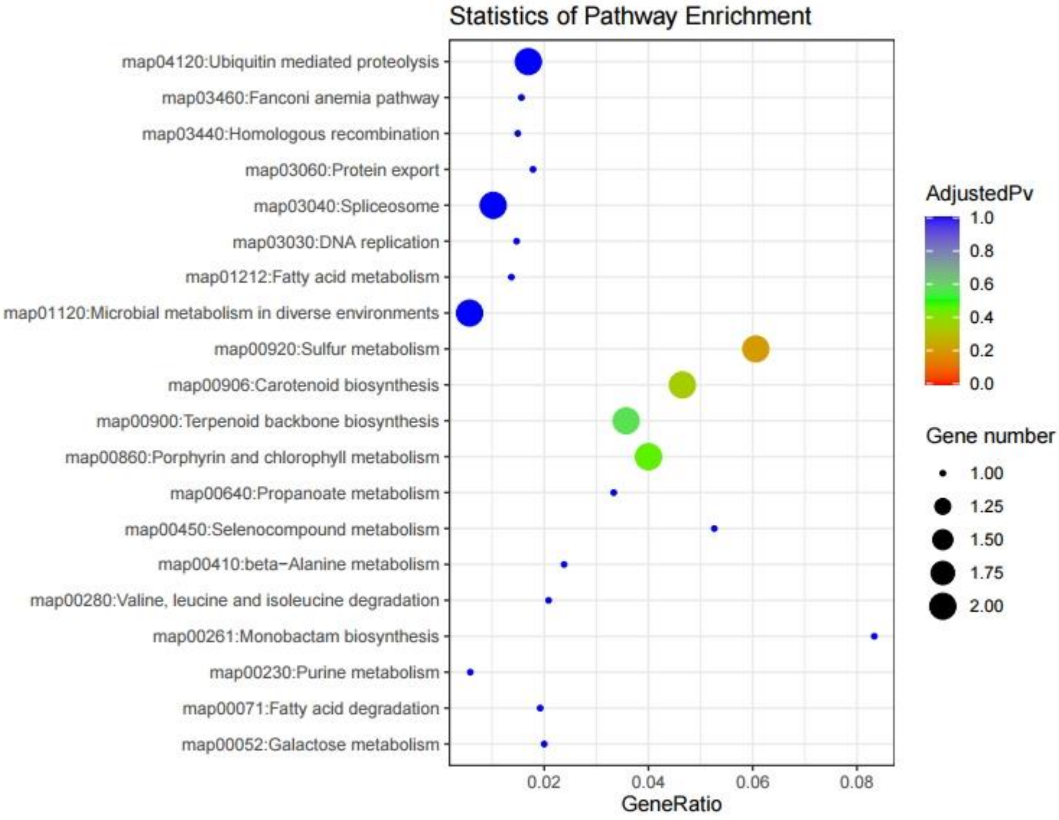

Supplementary Figure 18. KEGG enrichment pathways for microRNA target genes of *Ac. calamus* B. The size of the circle represents the number of genes, and the heatmap color bar represents the adjust *p*-value of enrichment.

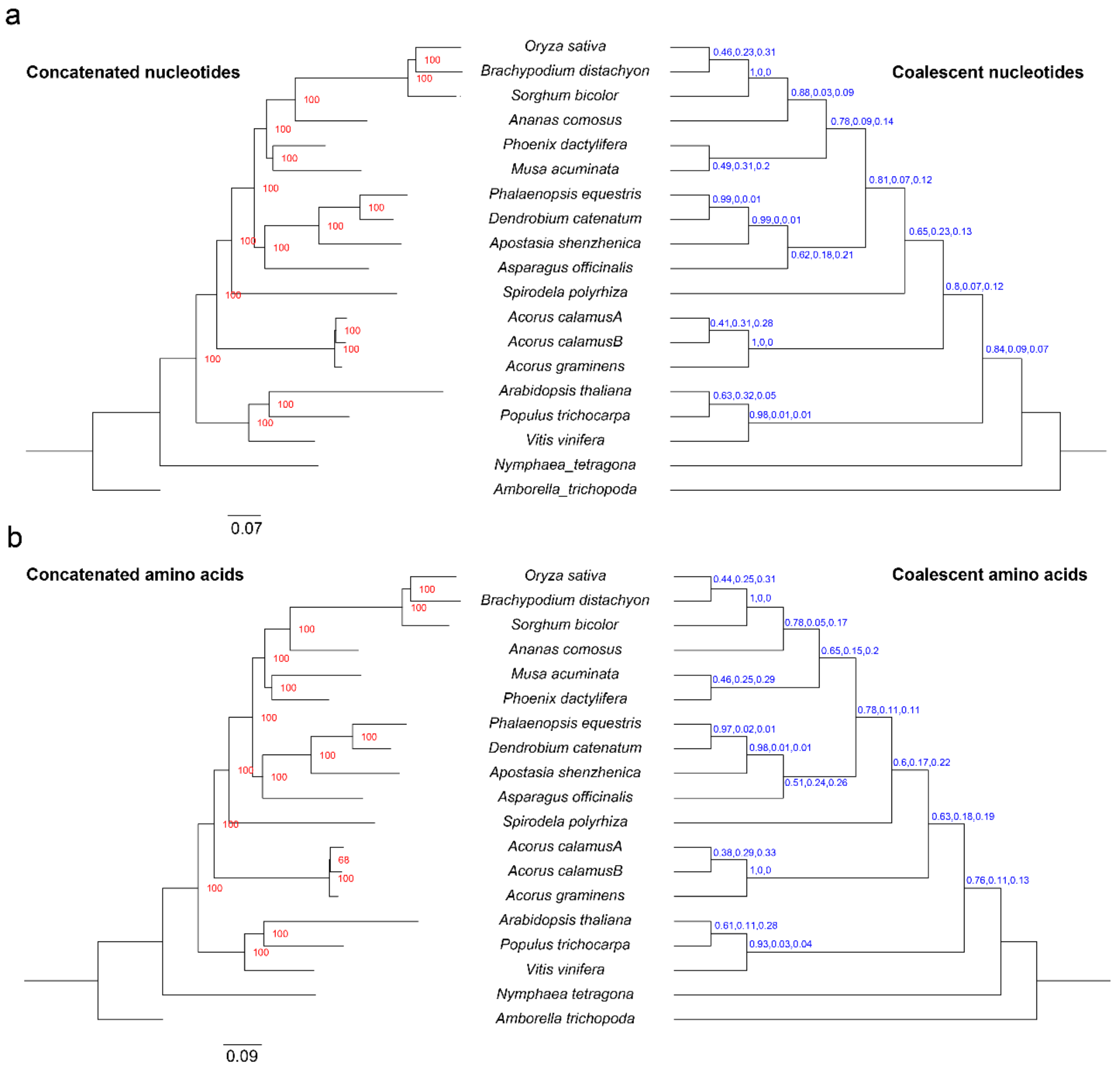

Supplementary Figure 19. Concatenated and multi-species coalescent (MSC) phylogenetic tree of *Ac. calamus*, *Ac. gramineus* and other 16 species. a The phylogenetic tree based on nucleotides. **b** The phylogenetic tree based on amino acids. The branch length of concatenated tree indicates the numbers of substitutions per site, and the red numbers indicates the bootstrap. The blue numbers on MSC tree indicates the q1, q2, and q3 refer to the quartet support for the main topology, the first alternative, and the second alternative.

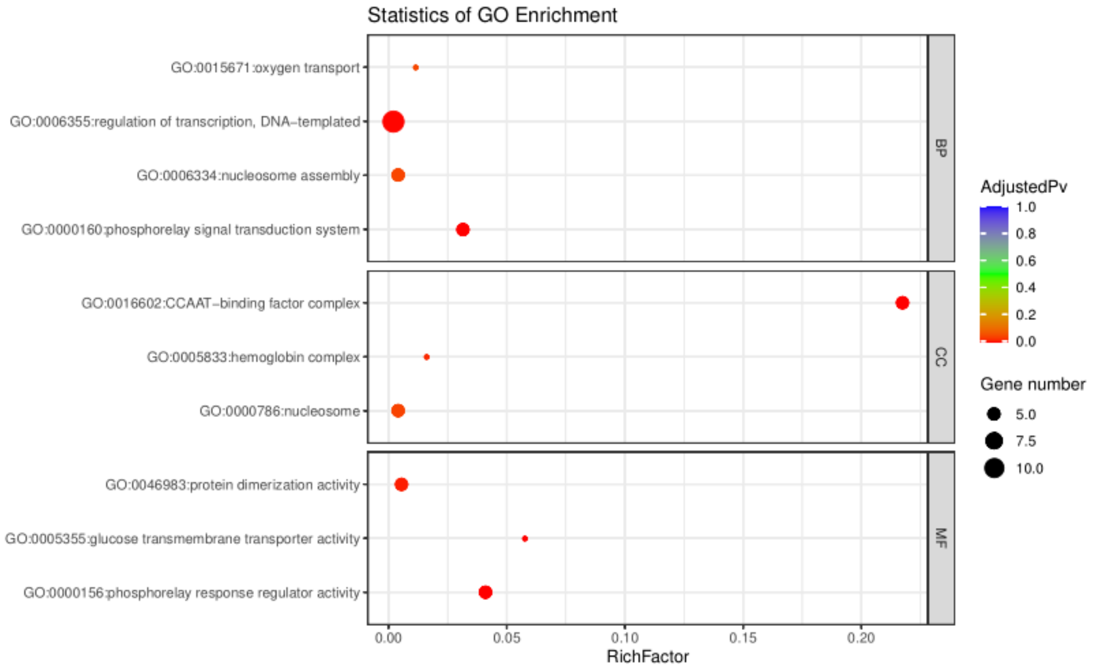

Supplementary Figure 20. GO enrichment terms for unique gene families of monocots. BP, MF, and CC represent Biological Process, Molecular Function, and Cellular Component groups of GO, respectively. The size of the circle represents the number of genes, and the heatmap color bar represents the adjust *p*-value of enrichment.

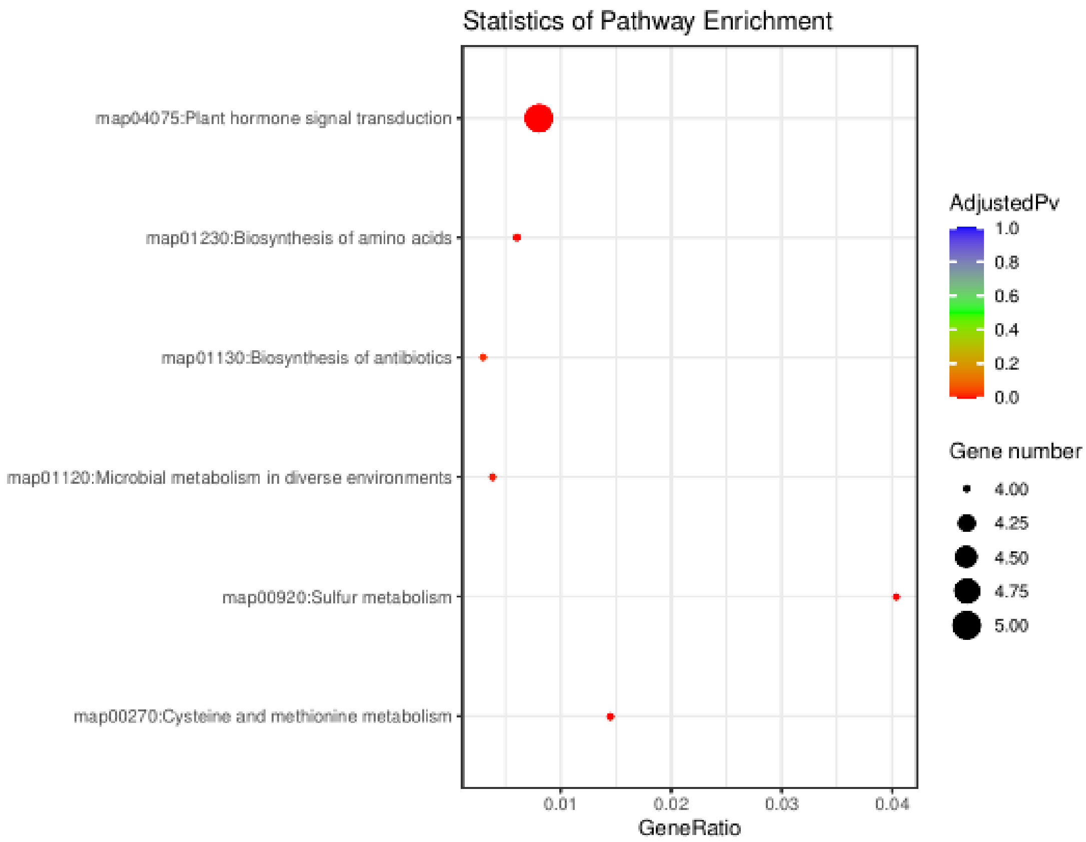

Supplementary Figure 21. KEGG enrichment pathways for unique gene families of monocots. The size of the circle represents the number of genes, and the heatmap color bar represents the adjust *p*-value of enrichment.

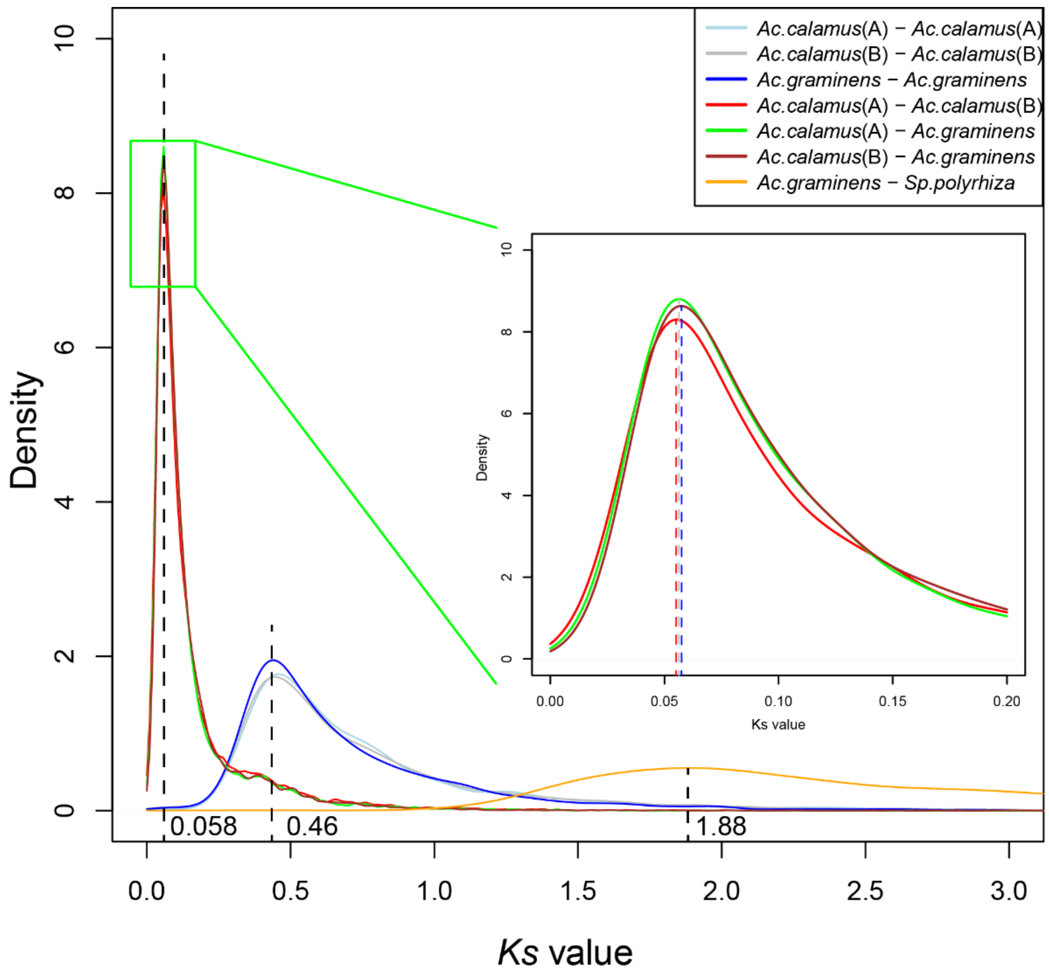

Supplementary Figure 22. ***Ks* distribution among *Ac. calamus* A, *Ac. calamus* B, *Ac. gramineus* and *Sp. polyrhiza*.** The enlarged part was the *K*s distribution of *Ac. calamus* A, *Ac. calamus* B and *Ac. gramineus*. The dashed lines showed the peaks of *K*s distribution.

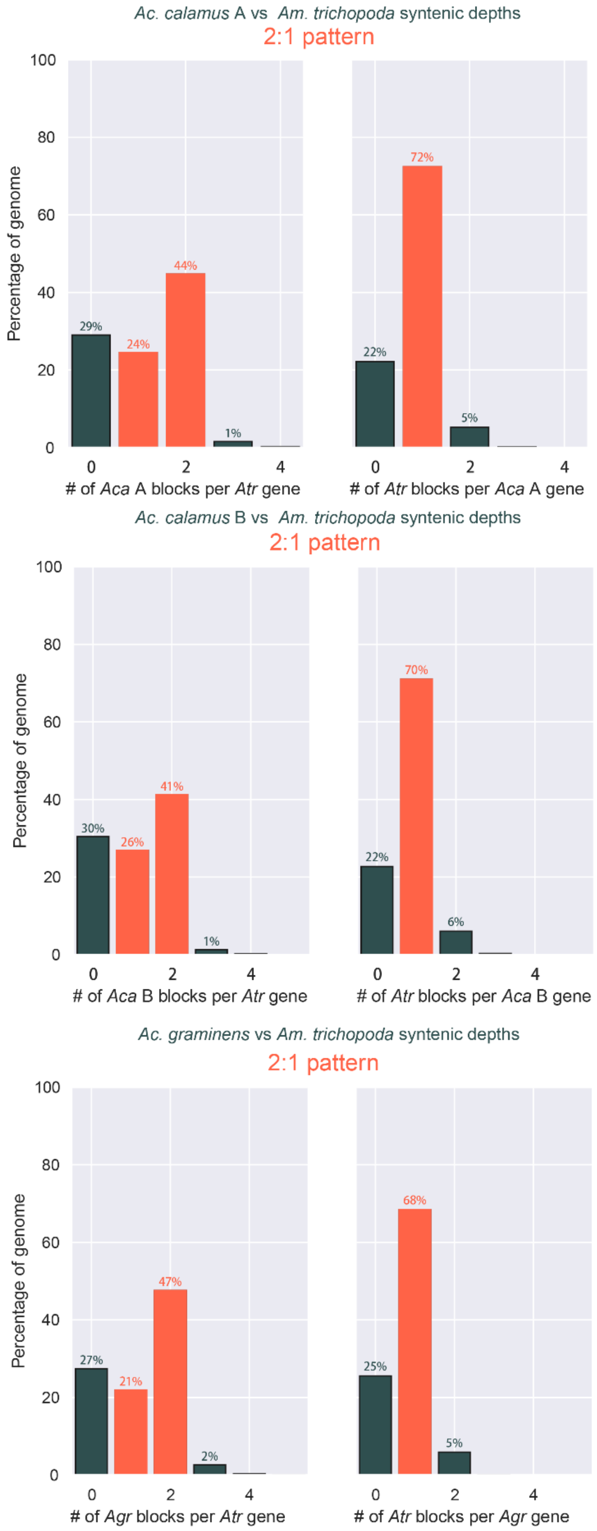

Supplementary Figure 23. The distribution of collinear block between *Ac. calamus* (A and B), *Ac. gramineus* and *A. trichopoda* syntenic depths. The red histogram shows the main pattern of collinear gene ratio between the two genomes. The black histogram shows the no-collinear region and the not-main pattern of collinear gene ratio. JCVI with default parameters was used to make the graph.

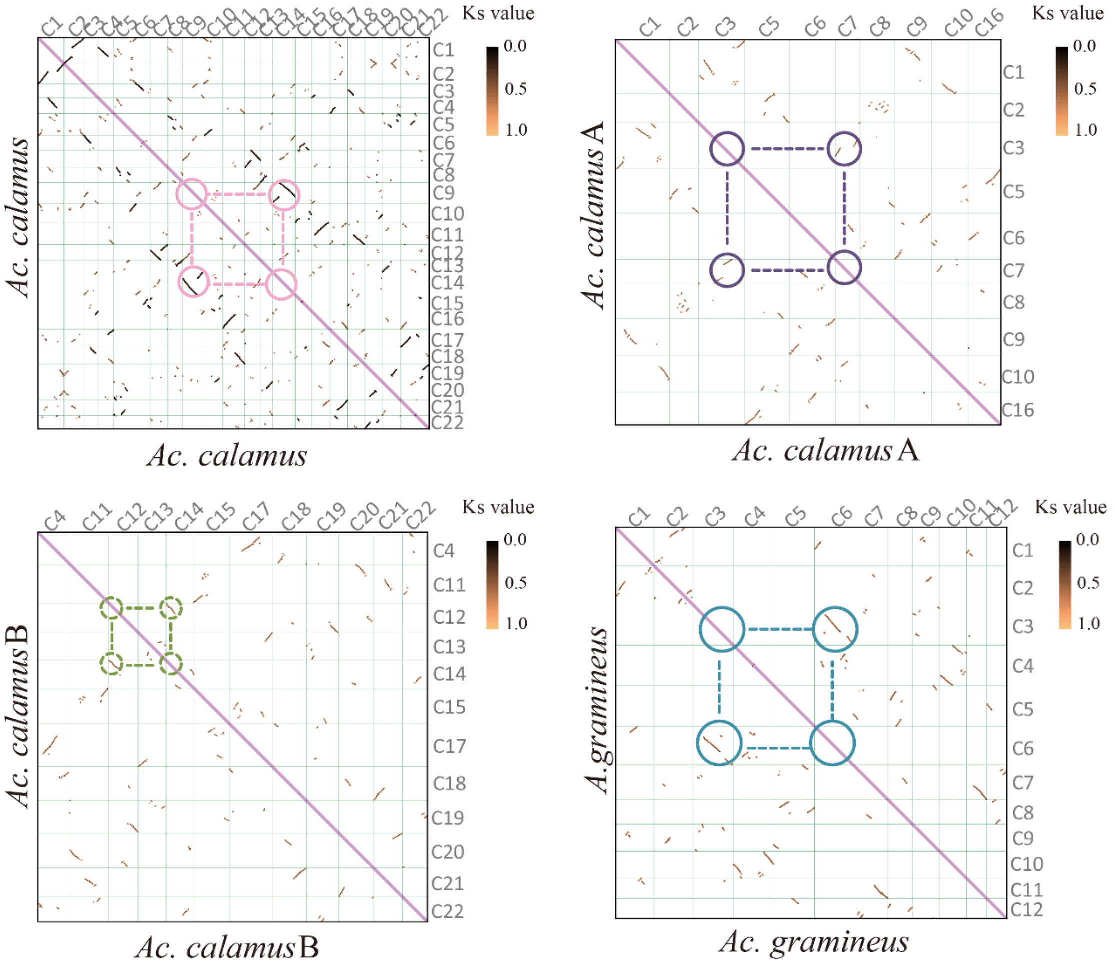

Supplementary Figure 24. Dot plots of paralogues with *K*s coloration in the *Ac. calamus* A (3-7 chromosomal relationships in purple circles), *Ac. Calamus* B (12-14 chromosomal relationships in green circles) and *Ac. gramineus* (3-6 chromosomal relationships in blue circles) genomes illustrating the shared WGD and paralogues in the *Ac. calamus* genome (9-14 chromosomal relationships in pink circles) clarifying the independent WGD event of *Ac. calamus*. The syntenic gene pairs are colored by the synonymous mutation values. Source data are provided as a Source Data file.

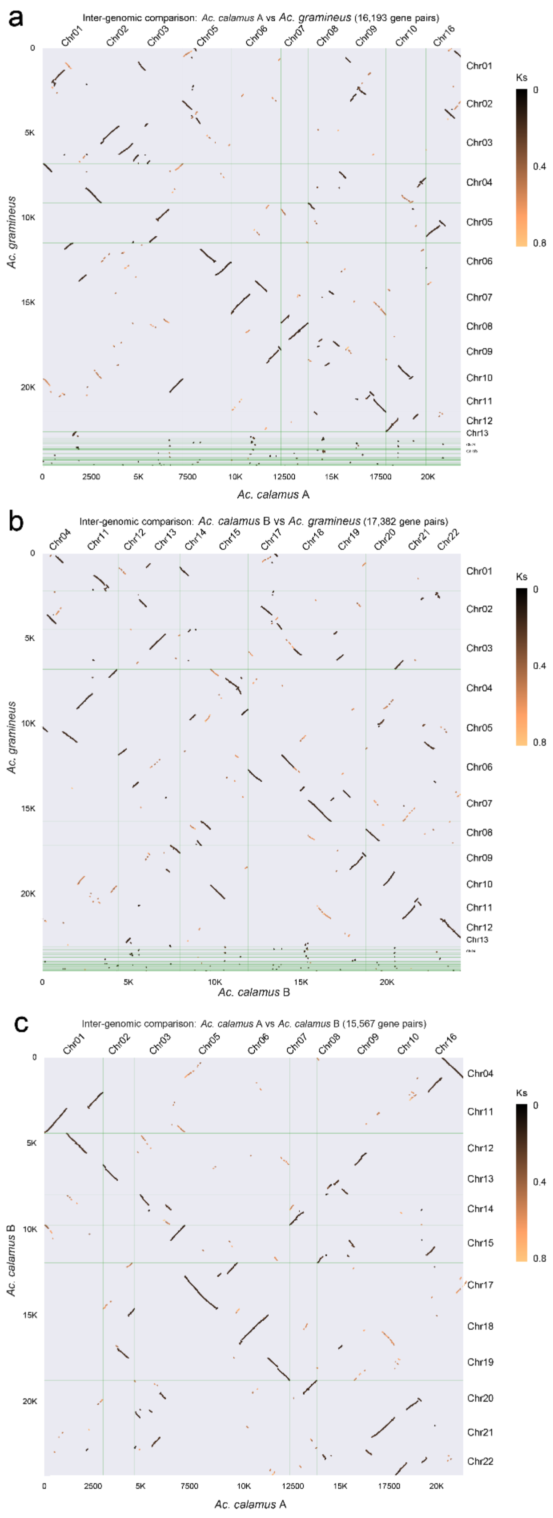

Supplementary Figure 25. Dot plots of orthologues. a *Ac. gramineus–Ac. calamus* A**. b** *Ac. gramineus–Ac. calamus* B**. c** *Ac. calamus* A–*Ac. calamus* B. The mean *K*s of each synteny block was calculated, the color-bar shows the color of mean *K*s. Source data are provided as a Source Data file.

a

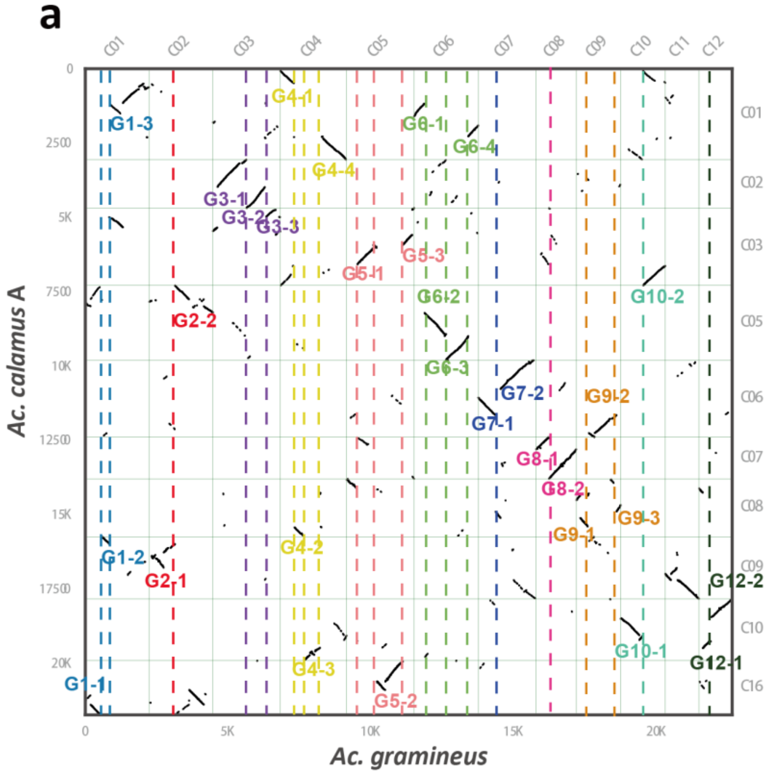

b

A1 = G4-1 + G1-3(6-1) + G6-4 + G4-4

A2 = G3-1 + G3-2

A3 = G3-3 + G1-3 + G5-3 + G5-1 + G10-2

A5=G2-2 + G6-2 + G6-3

A6=G7-2 + G7-1 + G9-2

A7=G8-1 + G8-2

A8=G9-3 + G9-1 + G4-2

A9=G1-2 + G2-1 + G11

A10=G12-2 + G10-1 + G12-1 + G4-3

A16=G5-2 + G1-1

Supplementary Figure 26. Alignment of *Ac. calamus* A chromosomes with *Ac. gramineus* chromosomes. **a** Genome synteny visualization by dot plots. Different colors represent different chromosomes. The labels on the plot represent different chromosome segments. **b** The collinearity between *Ac. calamus* A and *Ac. gramineus* chromosomes. The colors are the same as in (a). Source data are provided as a Source Data file.

a

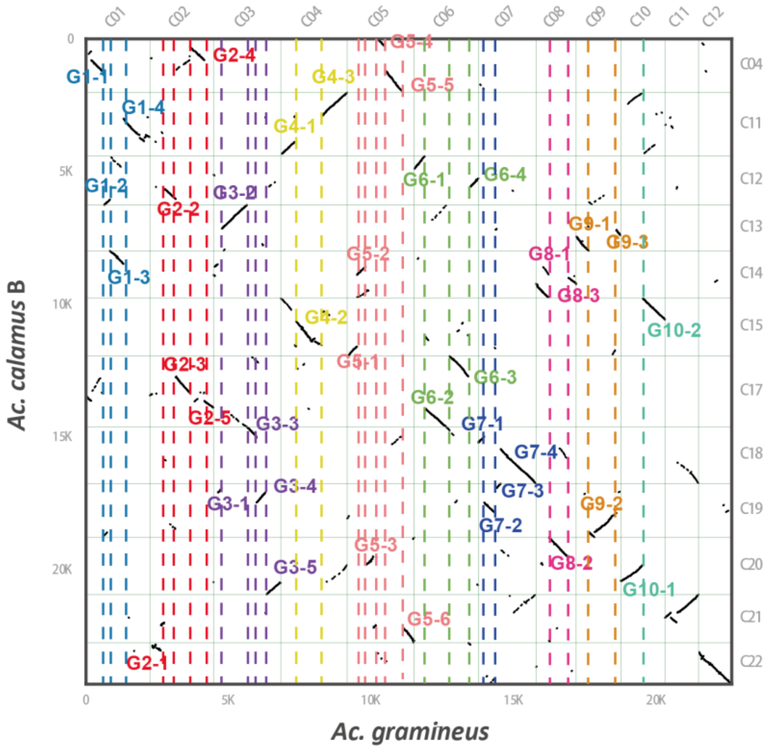

b

B4 = G5-4 + G2-4 + G1-1 + G5-5
B11 = G4-3 + G1-4 + G4-1
B12 = G6-1 + G6-4 + G2-2 + G1-2
B13=G3-2 + G9-3 + G9-1
B14=G1-3 + G8-1 +G8-3
B15=G10-2 + G4-2 + G5-1
B17=G6-3 + G2-3 + G2-5 + G6-2
B18=G3-3 + G7-1 +G7-4
B19=G7-3 + G3-1 + G3-4 + G7-2 + G9-2
B20=G8-2 + G5-3 + G10-1 + G3-5
B21=G11 + G5-6
B22=G2-1 + G12

Supplementary Figure 27. Alignment of *Ac. calamus* B chromosomes with *Ac. gramineus* chromosomes. a Genome synteny visualization by dot plots. Different colors represent different chromosomes. The labels on the plot represent different chromosome segments. **b** The collinearity between *Ac. calamus* B and *Ac. gramineus* chromosomes. The colors are the same as in (a). Source data are provided as a Source Data file.

a

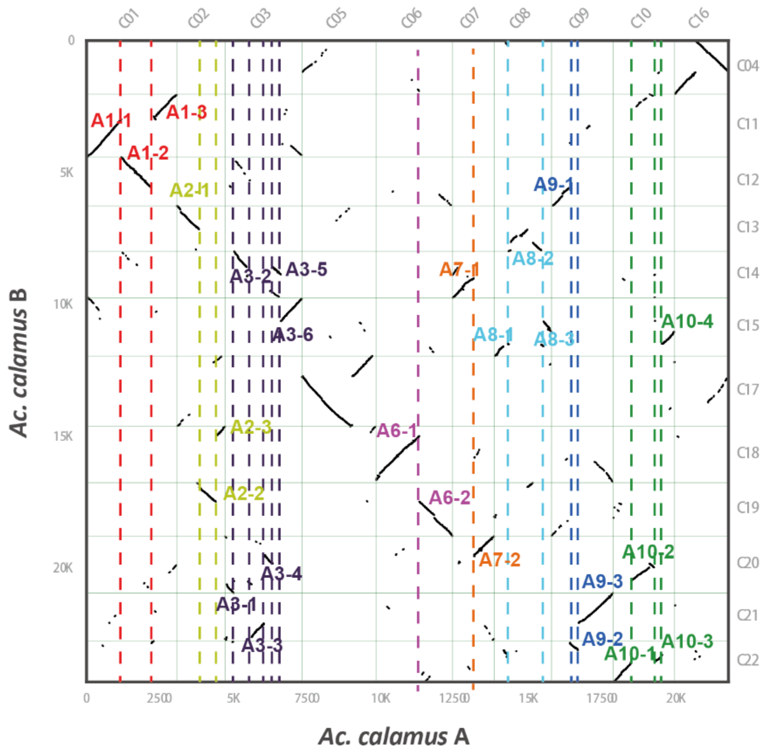

b

B4 = A16
B11 = A1-3 + A1-1
B12 = A1-2 + A9-1
B13=A2-1 + A8-2
B14=A3-2 + A3-5 + A7-1
B15=A3-6 + A8-3 + A10-4 + A8-1
B17=A5
B18=A2-3 + A6-1
B19=A2-2 + A6-2
B20=A7-2 + A3-4 + A10-2 + A3-1
B21=A9-3 + A3-3
B22=A9-2 + A10-3 + A10-1

Supplementary Figure 28. Alignment of *Ac. calamus* A chromosomes with *Ac. calamus* B chromosomes. a Genome synteny visualization by dot plots. Different colors represent different chromosomes. The labels on the plot represent different chromosome segments. **b** The collinearity between *Ac. calamus* A and *Ac. calamus* B chromosomes. The colors are the same as in (a). Source data are provided as a Source Data file.

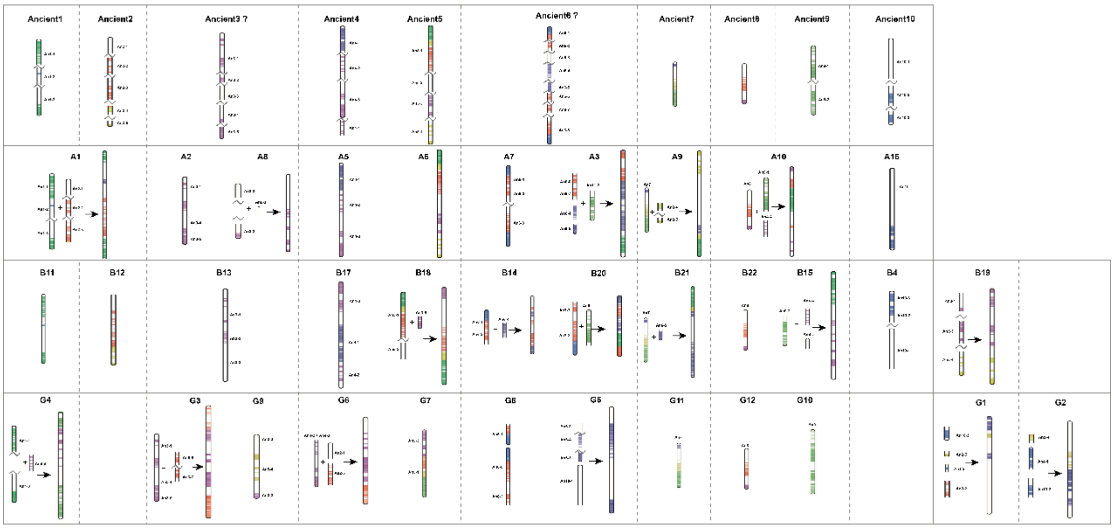

Supplementary Figure 29. The ancestral chromosomes reconstruction of most recent common ancestor (MRCA) for Acorales. The first line represents the presumed ancestral karyotype pattern of Acorales. Lines 2 to 4 represent the chromosome karyotype of *Ac. calamus* A, *Ac.* calamus B and *Ac. gramineus*, respectively, with each small panel representing the chromosome formation process. The composition of ancestral chromosomes in modern plant genomes is shown below, with different colors representing different ancestral chromosomes.

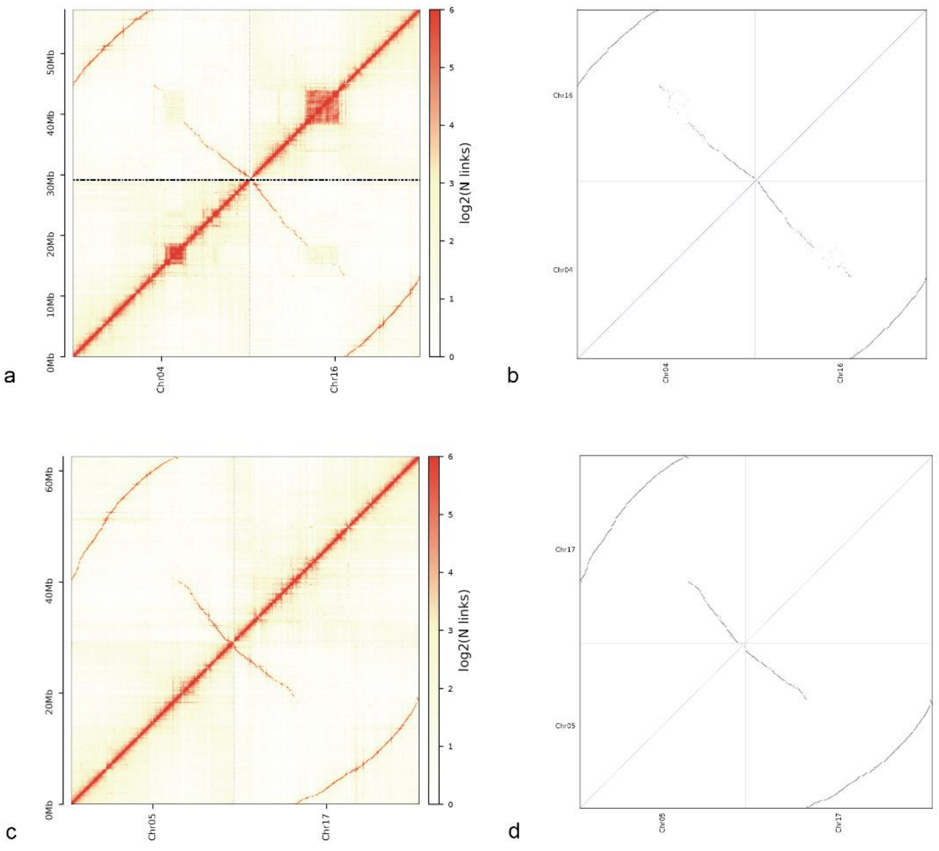

Supplementary Figure 30. Hi-C signal of *Ac. calamus* homologous chromosomes. a Hi-C heatmap of Chr04 and Chr16. **b** Collinearity map of Chr04 and Chr16. **c** Hi-C heatmap of Chr05 and Chr17. **d** Collinearity map of Chr05 and Chr17. The X-axis and Y-axis in the figure (a, b, c, d) are a pair of homologous chromosomes (Chr05, Chr16) from the subgenome A and B, the colorbar represents log2 normalized number of Hi-C valid reads, indicating the contact intensity of different genomic sites.

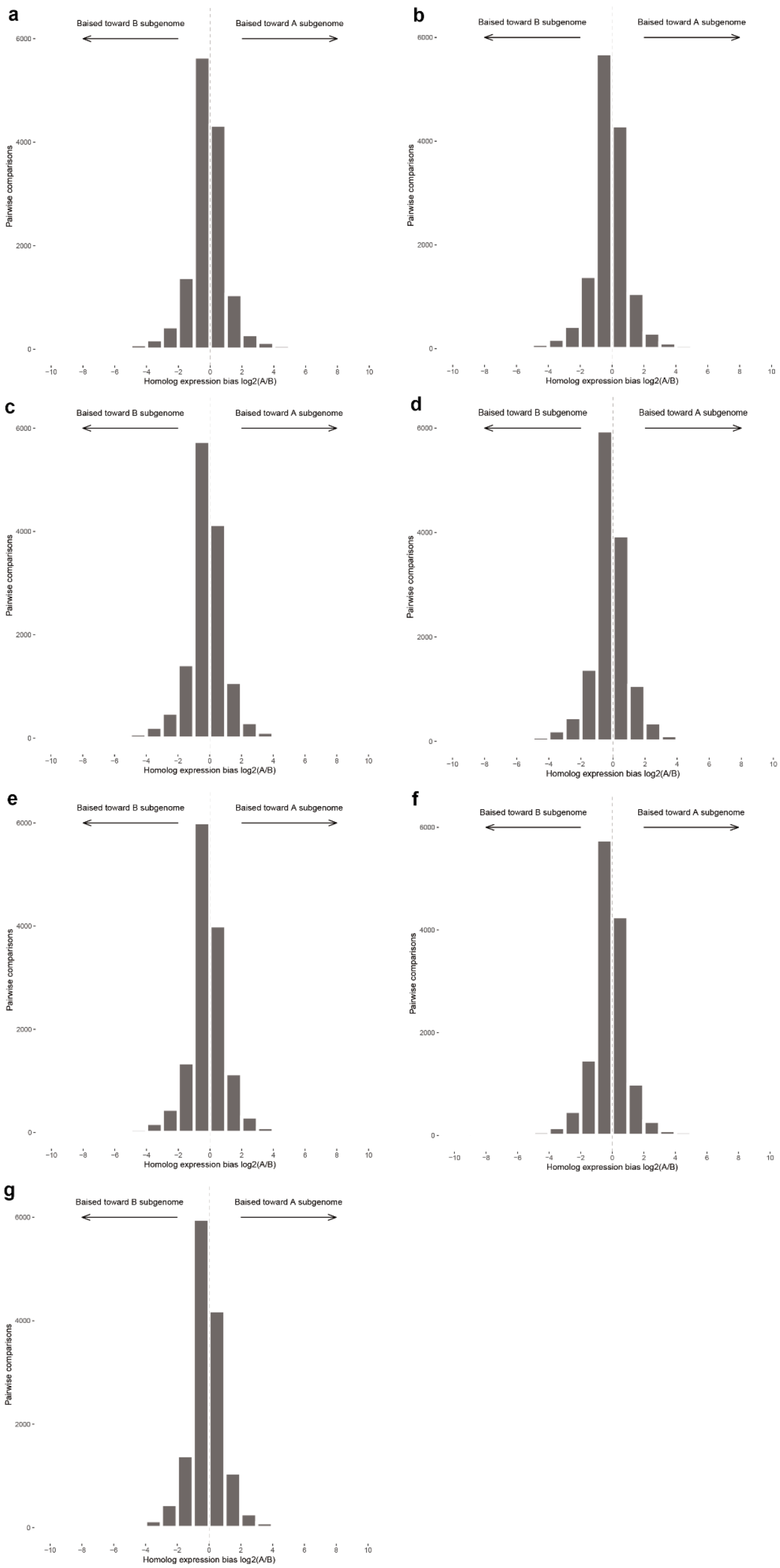
a
b
c
d
e
f
g
Baised toward B subgenome
Baised toward A subgenome
Pairwise comparisons
Homolog expression bias log2(A/B)
0
2000
4000
6000
−10
−8
−6
−4
−2
0
2
4
6
8
10

Supplementary Figure 31. The distribution of homolog expression bias (HEB) of homologous gene pairs in all tissues. HEB> 0 indicates a bias toward the subgenome A, and HEB <0 indicates a bias toward the subgenome B. **a** Flower. **b** Bract. **c** Inflorescence. **d** Peduncle. **e** Leaf. **f** Root. **g** Stem. X axis indicated the log2 normalized number of homolog expression bias, Y axis indicated the number of comparisons pairwise genes. Source data are provided as a Source Data file.

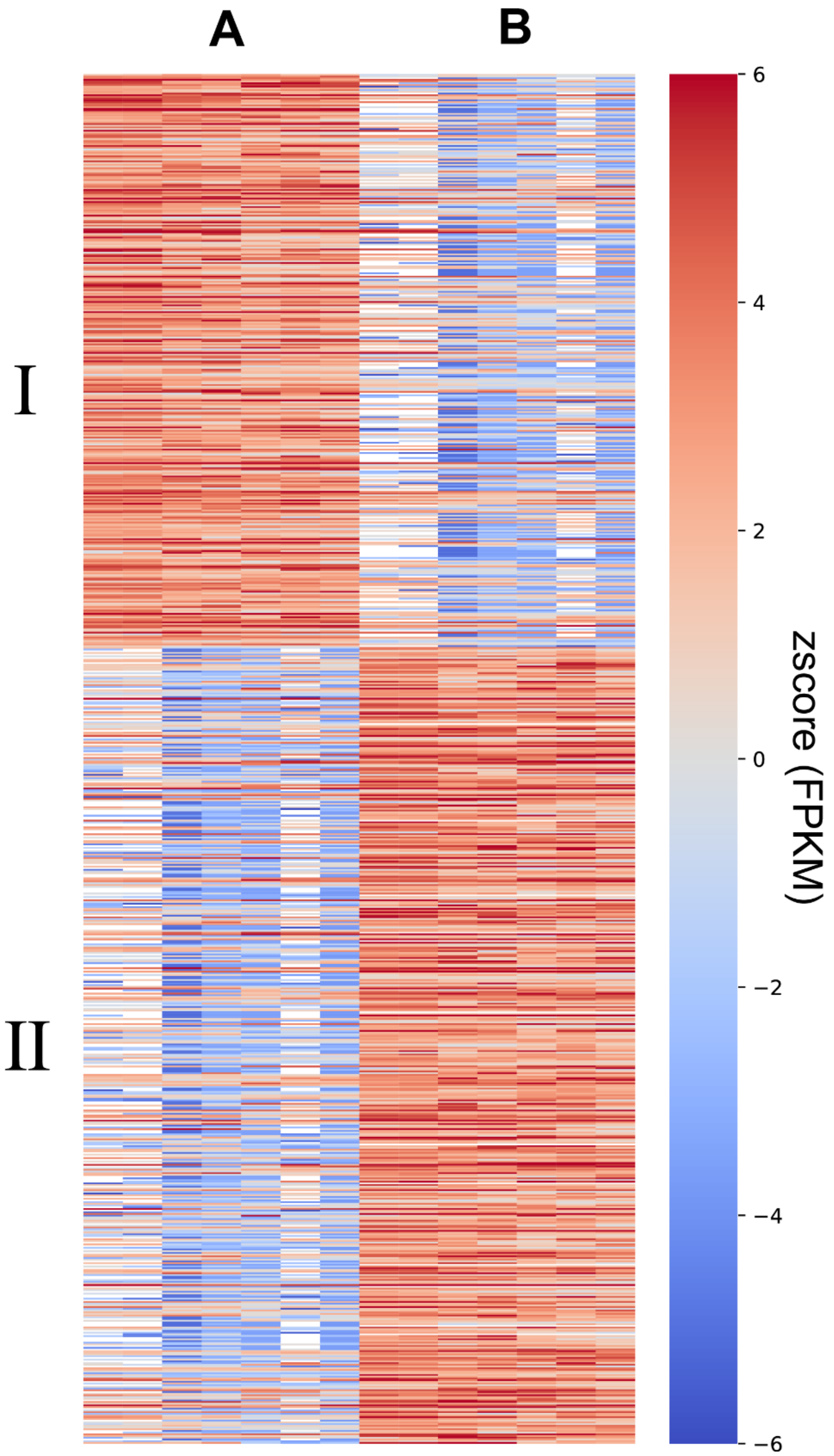

Supplementary Figure 32. Heatmaps of two extremely divergent co-expression clusters, transcripts per million (TPM) is used to measure gene expression levels, of which one of two homoeologous genes in subgenome A or B was extensively transcribed while the other copies suppressed in seven tissues. Cluster I, present A bias genes; Cluster II, present B bias genes. (FPKM, fragments per kilobase of exon per million fragments mapped for each predicted transcript). Source data are provided as a Source Data file.

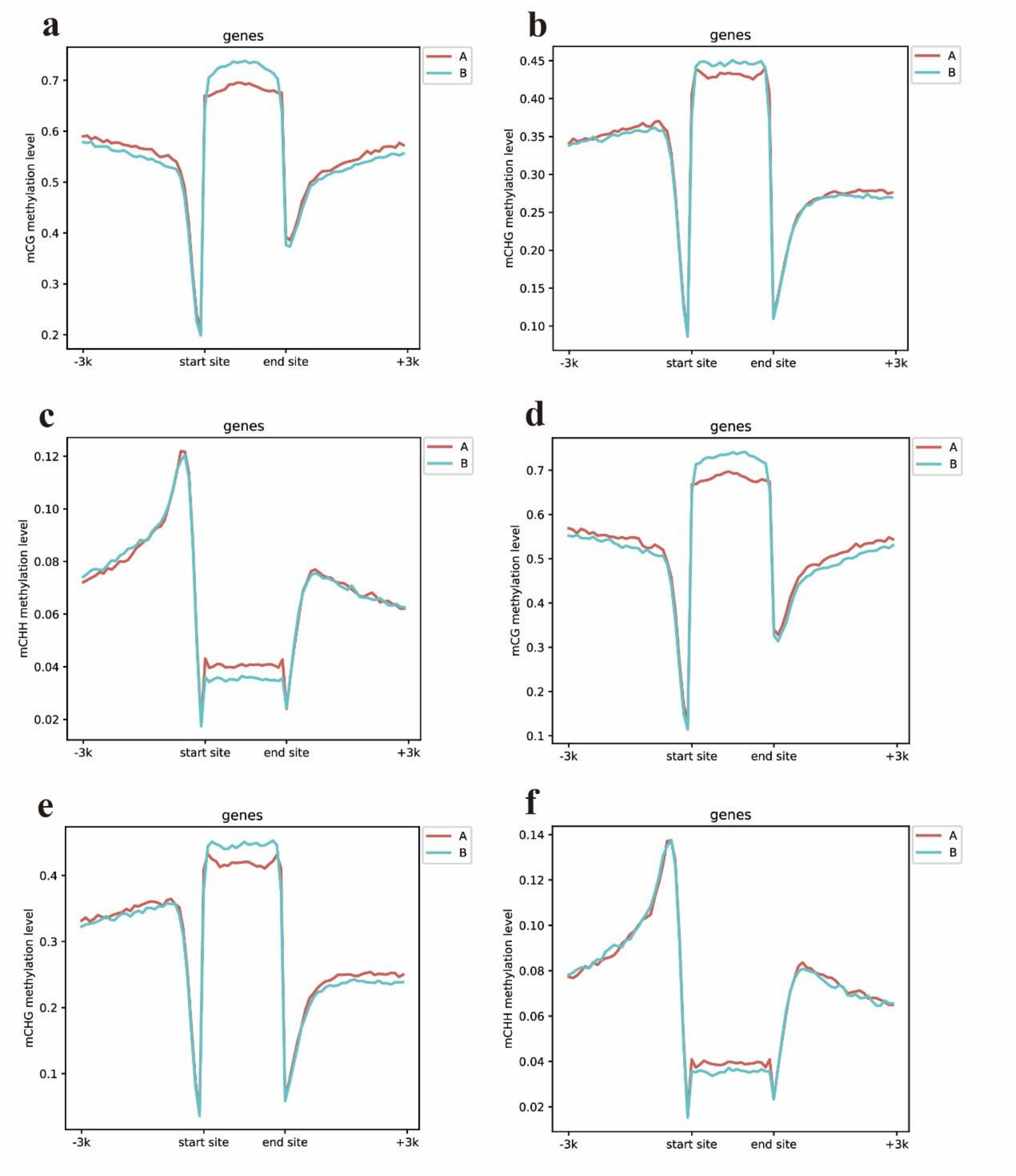

Supplementary Figure 33. The distribution of methylation level in the gene of upstream and downstream regions. a All genes CG methylation. **b** All genes CHG methylation. **c** All genes CHH methylation. **d** The homoeolog genes of subgenome A and B CG methylation. **e** The homoeolog genes of subgenome A and B CHC methylation. **f** The homoeolog genes of subgenome A and B CHH methylation. Each gene was divided into 20 intervals, 3 Kb upstream and downstream region of each gene were divided into 100 intervals. The red line (A) and blue line (B) represent the distribution of methylation level of subgenome A and B, respectively. Source data are provided as a Source Data file.

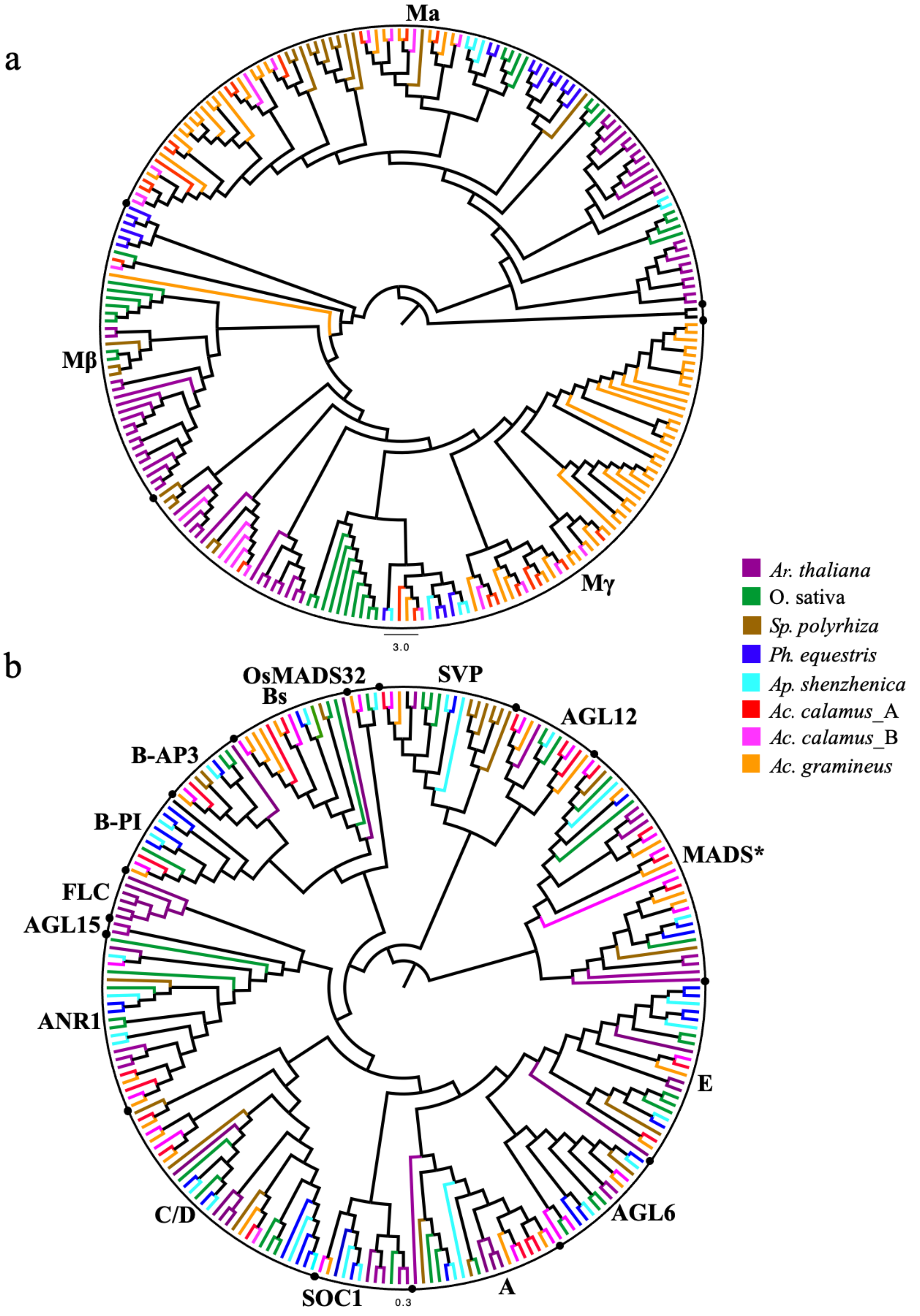

Supplementary Figure 34. Phylogenetic relationship of MADS-box genes in *A. thaliana*, *O. sativa*, *A. shenzhenica, P. equestris, S. polyrhiza, Ac. gramineus, Ac. calamus* A, and *Ac. calamus* B. a Phylogenetic tree of Type I MADS-box genes. **b** Phylogenetic tree of Type II MADS-box genes.

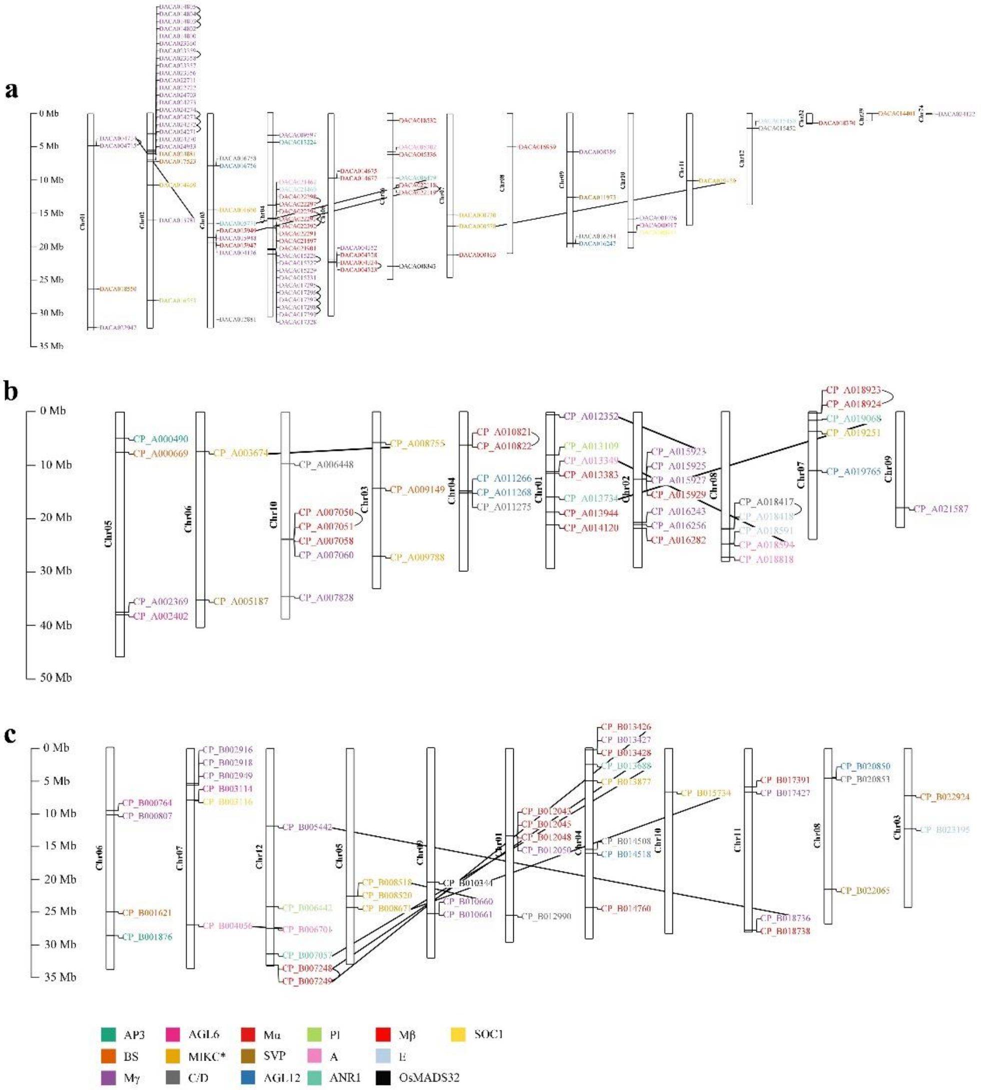

Supplementary Figure 35. Tandem duplication of MADS-box gene in *Acorus*. a Tandem duplication of MADS-box gene in *Ac. gramineus*. **b** Tandem duplication of MADS-box gene in *Ac. calamus* A. **c** Tandem duplication of MADS-box gene in *Ac. calamus* B. Lines of the same color represent the duplication of genes of the same subfamily between different chromosomes. The black curve indicates gene duplication. The different color represents different subclade of MADS-box gene.

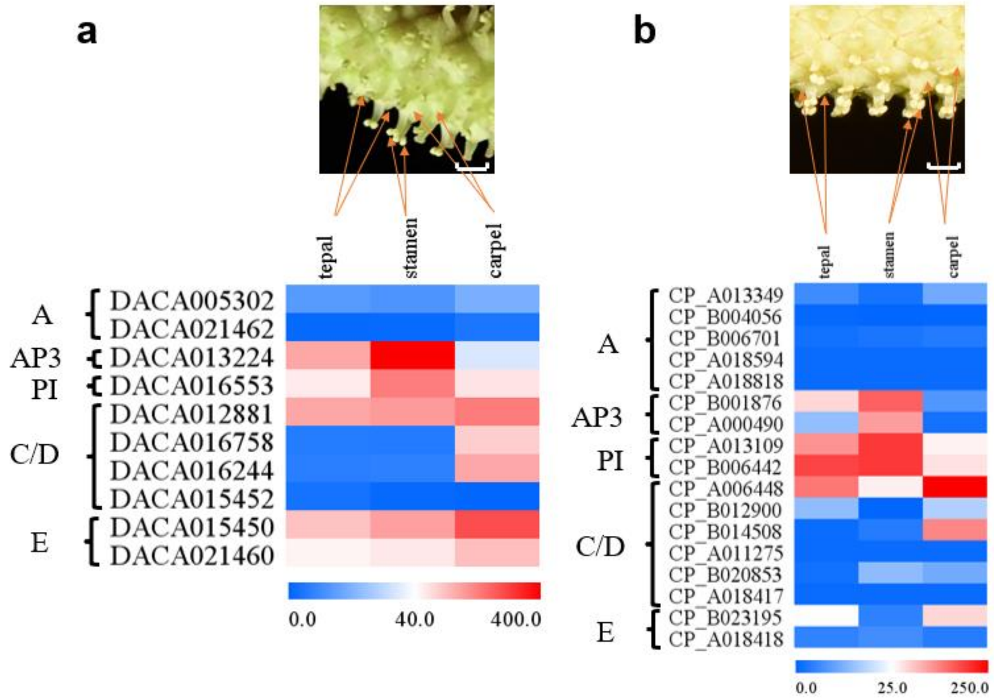

Supplementary Figure 36. Expression profiles of ABCDE MADS-box genes in *Acorus* floral organs. a Expression profiles of ABCDE MADS-box genes in *Ac. gramineus* floral organs. **b** Expression profiles of ABCDE MADS-box genes in *Ac. calamus* floral organs. The gene expression in both *Acorus* transcriptomes is represented by a color gradient from red to green. A heat map was generated to describe the expression level by FPKM of the genes. FPKM, fragments per kilobase of exon per million fragments mapped for each predicted transcript. Bar in the photos was 3 mm. Source data are provided as a Source Data file.

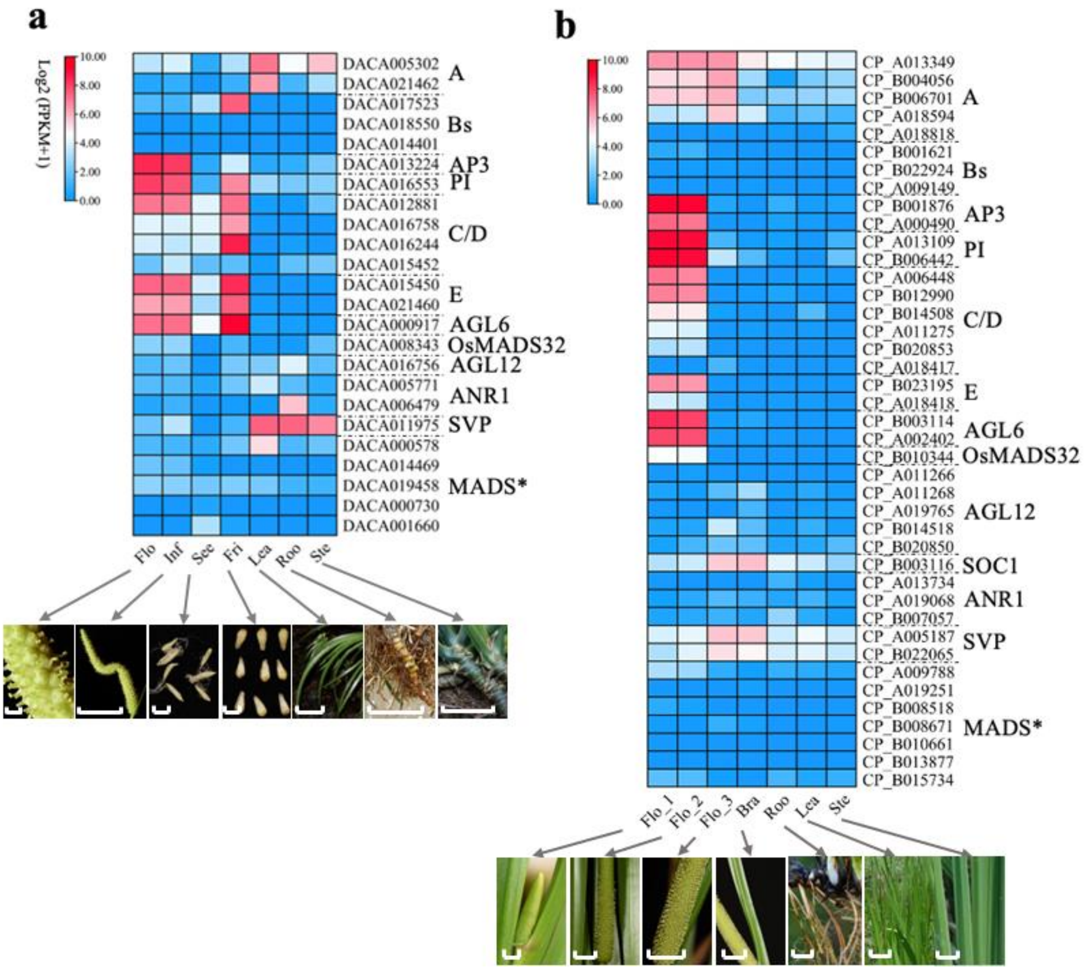

Supplementary Figure 37. Expression profile of MADS-box genes in reproductive organs and vegetative organs. a Expression profile of *Ac. gramineus* MADS Type II genes (Flo, flower; Inf, inflorescence; See, seed; Fri, fruit; Lea, leaf; Roo, root; Ste, stem). Bar, 2mm: flower, seed and fruit; 20 cm: inflorescence, leaf, root and stem. **b** Expression profile of *Ac. calamus* MADS Type II genes (Flo-1, flowering 1 stage (bud, diameter 1.5 mm); Flo-2, flowering 2 stage (flower is opening, diameter 2.5 mm); Flo-3, flowering 3 stage (flower full opened, diameter 4 mm); Bra, bract; Roo, root; Lea, leaf; Ste, stem). Bar, 1cm: flowering 1-3 stages, bract, root, stem; 10cm: leaf. Source data are provided as a Source Data file.

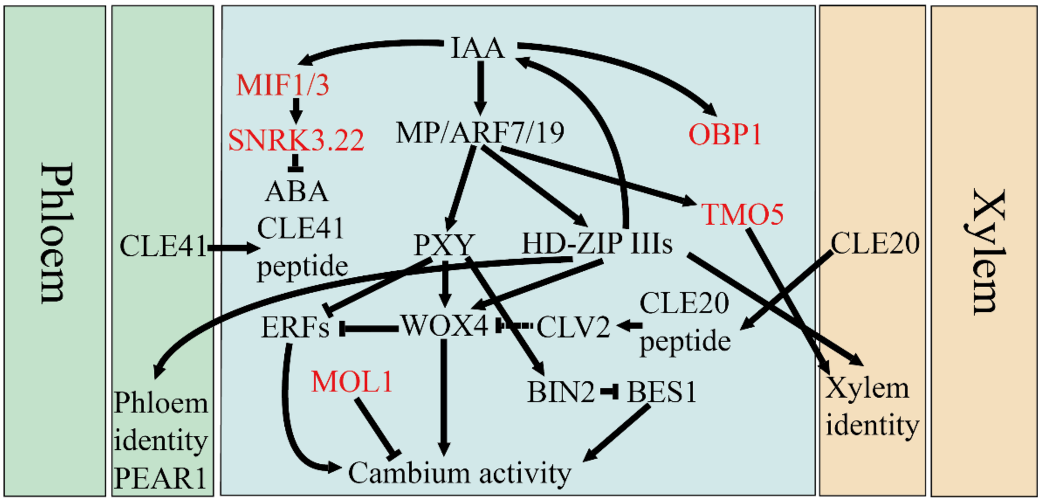

Supplementary Figure 38. Genes and regulation network of vascular cambial and secondary cell wall formation in angiosperms. Xylem and secondary cell wall development regulation network and molecular regulation mechanisms of phloem, cambium and xylem (reference to poplar) development[61,64,65]. Red color represents genes absent in monocots. The full name of genes please see Supplementary Data 9. Source data are provided as a Source Data file.

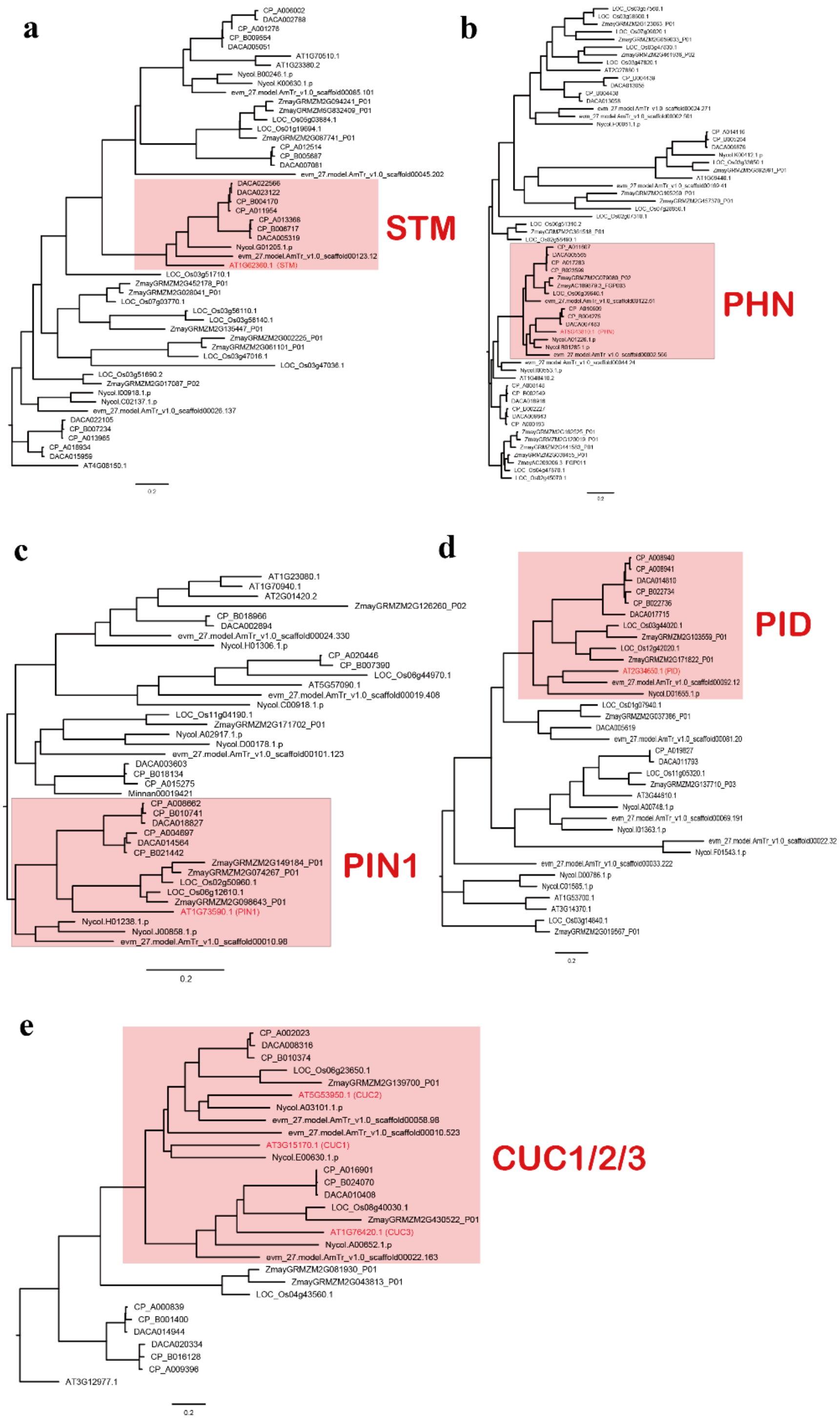

Supplementary Figure 39. Phylogenetic trees of cotyledon development related genes. a Phylogenetic tree based on STM. **b** Phylogenetic tree based on PHN. **c** Phylogenetic tree based on PIN1. **d** Phylogenetic tree based on PID. **e** Phylogenetic tree based on CUC1/2/3. CP_A, *Ac. calamus* A; CP_B, *Ac. calamus* B; DACA, *Ac. gramineus*; At, *Arabidopsis thaliana*; LOC, *Oryza sativa*; Zmay, *Zea mays*; Nycol, *Nymphaea colorata*; evm, *Amborella trichopoda.* Source data are provided as a Source Data file.

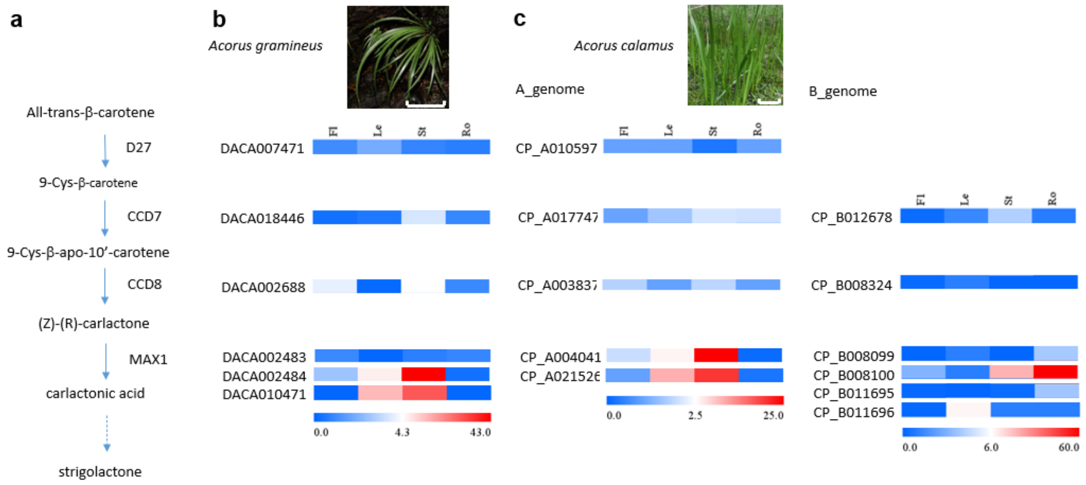

Supplementary Figure 40. Canonical biosynthetical pathway of strigolactone and the expression patterns of orthologous genes involved in each biosynthetic step of strigolactone in both *Acorus* species. The gene expression in both *Acorus* transcriptomes is represented by a color gradient from red to green. A heat map was generated to describe the expression level by FPKM of the genes among various tissues. **a** Canonical biosynthetical pathway of strigolactone and the genes involved in each biosynthetic step of strigolactone. D27: DWARF27, -carotene isomerase. CCD7: Carotenoid Cleavage Dioxygenases 7. CCD8: Carotenoid Cleavage Dioxygenases 8. MAX1: More Axillary Growth1, CYP711A. **b** The orthologous gene IDs with the expression level by FPKM in various tissues of Ac. gramineus. **c** The orthologous gene IDs with the expression level by FPKM in various tissues in A and B genomes of *Ac. calamus*. Fl: flowers; Le: leaves; St: stems; Ro: roots. Bar in the photos of *Ac. gramineus* and *Ac. calamus* are 20 cm. Source data are provided as a Source Data file.

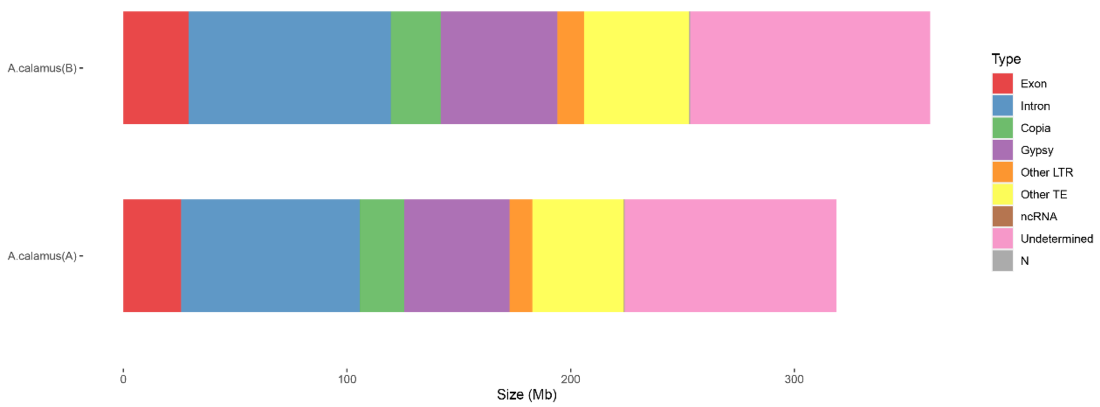

Supplementary Figure 41. Genome content of *Ac. calamus* A and *Ac. calamus* B. Source data are provided as a Source Data file.

a

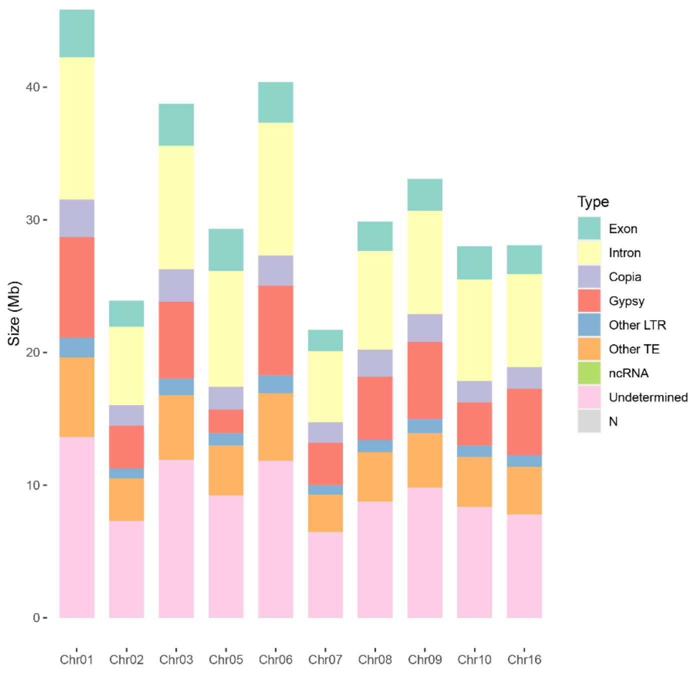

b

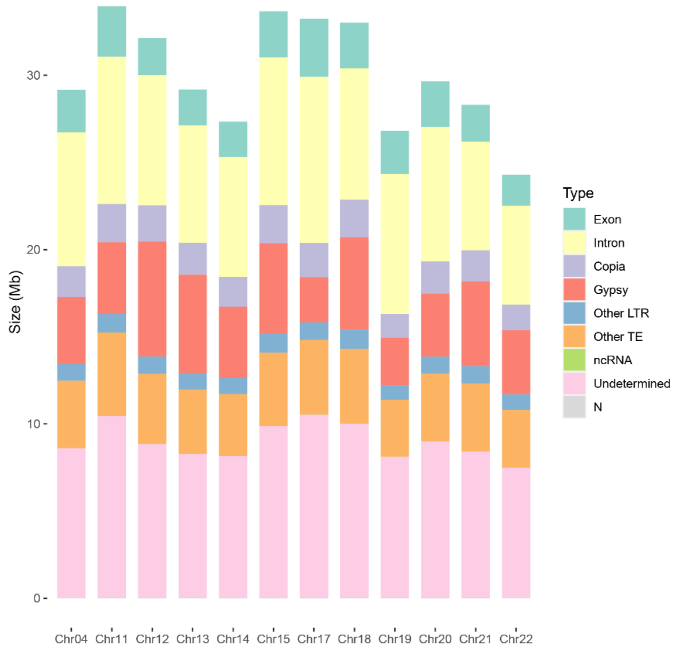

Supplementary Figure 42. Genic elements of each chromosome in *Ac. calamus* A and *Ac. calamus* B. a *Ac. calamus* A. **b** *Ac. calamus* B. Source data are provided as a Source Data file.

Supplementary Table 1. Statistics of PacBio sequencing data in *Acorus* genomes.

	**Sample**	**Average length (Kb)**	**N50 length (Kb)**	**Total bases (Gb)**
Ac. gramineus	D-Agr-1	13.475	18.735	12.37
	D-Agr-1	11.265	16.724	13.21
	D-Agr-1	12.651	17.930	13.71
	D-Agr-1	14.744	19.797	17.83
	Total	13.034	18.297	57.12
Ac. calamus	R-Aca-1	9.421	15.212	8.46
	R-Aca-1	10.689	16.528	10.44
	R-Aca-1	13.665	19.124	13.16
	R-Aca-1	14.378	19.582	18.21
	R-Aca-1	15.106	19.400	17.65
	R-Aca-1	14.943	19.291	18.53
	Total	13.274	18.189	86.45

Supplementary Table 2. Assembly results of *Acorus* genomes.

		Contig size (bp)	Contig number
Ac. gramineus	N50	1,740,860	61
	N60	1,324,149	87
	N70	971,479	121
	N80	642,575	170
	N90	307,780	256
	Longest	7,876,884	-
	Total length	391,633,588	-
	Total	-	670
Ac. calamus	N50	873,187	237
	N60	714,686	325
	N70	531,390	440
	N80	378,724	595
	N90	195,863	845
	Longest	4,519,085	-
	Total length	700,938,827	-
	Total	-	1680

Supplementary Table 3. BUSCO assessment of *Acorus* genome assemblies.

	Type	Number	Percentage
Ac. gramineus	Complete BUSCOs	1,555	96.34%
	Complete and single-copy BUSCOs	1,457	90.27%
	Complete and duplicated BUSCOs	98	6.07%
	Fragmented BUSCOs	23	1.43%
	Missing BUSCOs	36	2.23%
	Total BUSCO groups searched	1,614	100%
Ac. calamus	Complete BUSCOs	1,551	96.10%
	Complete and single-copy BUSCOs	386	23.92%
	Complete and duplicated BUSCOs	1,165	72.18%
	Fragmented BUSCOs	27	1.67%
	Missing BUSCOs	36	2.23%
	Total BUSCO groups searched	1,614	100%

Supplementary Table 4. Genomic BUSCO assessment of *Acorus calamus* and other monocotyledonous species.

Species	Complete BUSCOs (C)	Complete and single-copy BUSCOs (S)	Complete and duplicated BUSCOs (D)	Fragmented BUSCOs (F)	Missing BUSCOs (M)	Total BUSCO groups searched
Ac. calamus	1551/96.10%	386/23.92%	1165/72.18%	27/1.67%	36/2.23%	1614/-
Ac. gramineus	1555/96.34%	1457/90.27%	98/6.07%	23/1.43%	36/2.23%	1614/-
O. sativa	1592/98.64%	1559/96.59%	33/2.04%	14/0.87%	8/0.50%	1614/-
S. viridis	1585/98.20%	1556/96.41%	29/1.80%	15/0.93%	14/0.87%	1614/-
*Z. mays*_B73	1582/98.02%	1432/88.72%	150/9.29%	17/1.05%	15/0.93%	1614/-
*Z. mays*_SK	1584/98.14%	1436/88.97%	148/9.17%	15/0.93%	15/0.93%	1614/-

Supplementary Table 5. Statistics of Hi-C data in *Acorus* genome.

	**Raw reads**	**Raw base (Gb)**	**Clean reads**	**Raw base (Gb)**
Ac. gramineus	336,231,826	50.43	228,731,478	34.31
Ac. calamus	442,011,284	66.30	319,342,656	47.90

Supplementary Table 6. Hi-C assembly result of *Ac. gramineus* and *Ac. calamus* subgenomes A and B.

		Contig Length (bp)	Contig number	Scaffold Length (bp)	Scaffold number
Ac. gramineus	N50	1,580,934	65	24,594,884	7
	N90	279,634	276	279,634	30
	Total	392,811,865		393,100,365	
Ac. calamus A	N50	743,083	131	29,860,984	5
	N90	163,349	481	24,887,880	9
	Total	323,327,160		323,787,663	
Ac. calamus B	N50	704,000	149	29,836,090	6
	N90	163,636	560	26,806,862	11
	Total	360,994,039		360,994,039	

Supplementary Table 7. Chromosome length of *Ac. gramineus.*

Chromosome ID	Length (bp)
Chr01	32,551,433
Chr02	32,347,091
Chr03	32,255,118
Chr04	30,642,109
Chr05	30,404,444
Chr06	24,942,200
Chr07	24,594,884
Chr08	21,040,368
Chr09	20,271,002
Chr10	20,241,715
Chr11	16,839,742
Chr12	13,733,618
Total	299,863,724

Supplementary Table 8. Chromosome length of *Ac. calamus* subgenomes A and B.

	Chromosome ID	Length (bp)
Ac. calamus A	Chr01	29,313,663
	Chr02	29,171,504
	Chr03	33,077,416
	Chr05	45,827,965
	Chr06	40,371,490
	Chr07	23,895,916
	Chr08	28,012,494
	Chr09	21,694,031
	Chr10	38,726,745
	Chr16	33,963,148
Ac. calamus B	Chr04	29,863,984
	Chr11	29,640,090
	Chr12	27,335,756
	Chr13	24,301,520
	Chr14	29,184,708
	Chr15	33,017,361
	Chr17	33,667,404
	Chr18	26,815,862
	Chr19	32,138,992
	Chr20	28,306,594
	Chr21	28,078,984
	Chr22	33,242,725

Supplementary Table 9. Statistics results of repeat sequencing in *Ac. gramineus*, *Ac. calamus* A and *Ac. calamus* B genomes.

**Type**	***Ac. gramineus***		***Ac. calamus* A**		***Ac. calamus* B**	
	**Repeat size**	**% of genome**	**Repeat size**	**% of genome**	**Repeat size**	**% of genome**
Trf	15,917,272	4.05	12,583,564	3.93	14,153,072	3.93
Repeatmasker	23,593,017	6.01	16,277,674	5.09	18,066,720	5.02
Proteinmask	23,144,034	5.89	12,148,847	3.8	13,575,410	3.77
De novo	190,351,012	48.46	138,687,718	43.35	158,686,152	44.12
Total	198,593,951	50.56	145,662,372	45.53	166,524,711	46.3

Supplementary Table 10. Classification of repeat sequence in *Ac. gramineus*, *Ac. calamus* A and *Ac. calamus* B genomes.

Species	Type	Repbase TEs		TE proteins		De novo		Combined TEs	
		Length (bp)	% in genome	Length (bp)	% in genome	Length (bp)	% in genome	Length (bp)	% in genome
Ac. gramineus	DNA	3,576,687	0.91	699,998	0.18	35,956,098	9.15	38,053,714	9.69
	LINE	705,245	0.18	1,009,596	0.26	7,805,464	1.99	8,799,421	2.24
	SINE	7,192	0.00	0	0.00	770,700	0.2	777,113	0.20
	LTR	19,166,842	4.88	21,434,664	5.46	138,169,094	35.17	139,883,638	35.61
	Other	716	0.00	0	0.00	0	0	716	0.00
	Unknown	51,144	0.01	0	0.00	12,900,864	3.28	12,951,860	3.30
	Total	23,593,017	6.01	23,144,034	5.89	185,135,452	47.13	188,809,789	48.07
Ac. calamus A	DNA	3,833,810	1.20	74,376	0.02	32,637,257	10.2	34,457,902	10.77
	LINE	1,202,453	0.38	0	0.00	8,029,383	2.51	8,787,712	2.75
	SINE	5,192	0.00	0	0.00	711,018	0.22	715,714	0.22
	LTR	11,301,780	3.53	12,074,471	3.77	88,315,339	27.6	89,376,286	27.93
	Other	1,679	0.00	0	0.00	0	0	1,679	0.00
	Unknown	36,014	0.01	0	0.00	17,211,239	5.38	17,247,143	5.39
	Total	16,277,674	5.09	12,148,847	3.80	134,964,003	42.18	137,316,929	42.92
Ac. calamus B	DNA	4,130,193	1.15	120616	0.03	36,958,799	10.28	39,040,596	10.85
	LINE	1,347,305	0.37	0	0.00	8,946,364	2.49	9,855,530	2.74
	SINE	5,642	0.00	0	0.00	884,497	0.25	889,811	0.25
	LTR	12,626,549	3.51	13,454,813	3.74	101,322,185	28.17	102,494,656	28.50
	Other	1,848	0.00	0	0.00	0	0	1,848	0.00
	Unknown	39,249	0.01	0	0.00	19,945,833	5.55	19,984,831	5.56
	Total	18,066,720	5.02	13,575,410	3.77	154,537,344	42.96	157,140,527	43.69

Supplementary Table 11. The Statistical results of *Ac. gramineus* TE annotation by EDTA.

Class	Count	bp Masked	%masked
LTR	--	--	--
Copia	19293	15362758	3.91%
Gypsy	78841	62574173	15.93%
unknown	84656	40164111	10.22%
TIR	--	--	--
CACTA	23584	6670710	1.70%
Mutator	79571	27476827	6.99%
PIF_Harbinger	29337	8772373	2.23%
Tc1_Mariner	1889	385725	0.10%
hAT	25568	8646413	2.20%
nonLTR	--	--	--
LINE_element	1200	657254	0.17%
unknown	139	28216	0.01%
nonTIR	--	--	--
helitron	39488	10109779	2.57%
unknown	50341	14546261	3.70%
Total	433907	195394600	49.74%

Supplementary Table 12. The Statistical results of *Ac. calamus* A TE annotation by EDTA.

Class		Count	bp Masked	%masked
LTR		--	--	--
	Copia	14857	11422848	3.59%
	Gypsy	32078	31580400	9.92%
	unknown	44674	29615668	9.30%
TIR		--	--	--
	CACTA	24836	6950743	2.18%
	Mutator	75303	24047548	7.55%
	PIF_Harbinger	34097	9324012	2.93%
	Tc1_Mariner	2966	792894	0.25%
	hAT	24082	7917970	2.49%
nonLTR		--	--	--
	LINE_element	1537	773417	0.24%
nonTIR		--	--	--
	helitron	41808	11739185	3.69%
	unknown	39120	10226059	3.21%
Total		335358	144390744	45.35%

Supplementary Table 13. The Statistical results of *Ac. calamus* B TE annotation by EDTA.

Class	Count	bp Masked	%masked
LTR	--	--	--
Copia	21781	12867960	3.57%
Gypsy	37785	40606478	11.27%
unknown	47075	29573705	8.21%
TIR	--	--	--
CACTA	25109	7267381	2.02%
Mutator	92652	28806553	8.00%
PIF_Harbinger	29864	9012123	2.50%
Tc1_Mariner	1686	317376	0.09%
hAT	32799	9867159	2.74%
nonLTR	--	--	--
LINE_element	1744	862515	0.24%
nonTIR	--	--	--
helitron	45770	12788293	3.55%
unknown	48228	12280890	3.41%
Total	384493	164250433	45.60%

Supplementary Table 14. The Statistical results of *Ac. calamus* TE annotation by EDTA.

Class	Count	bp Masked	%masked
LTR	--	--	--
Copia	36047	24937036	3.67%
Gypsy	76613	63667950	9.38%
unknown	126616	71124435	10.48%
TIR	--	--	--
CACTA	60554	17049590	2.51%
Mutator	180561	58059475	8.56%
PIF_Harbinger	67925	18984436	2.80%
Tc1_Mariner	8749	2105059	0.31%
hAT	58989	18273810	2.69%
nonLTR	--	--	--
LINE_element	2268	1114508	0.16%
nonTIR	--	--	--
helitron	100687	28487144	4.20%
unknown	90895	27071448	3.99%
Total	809904	330874891	48.76%

Supplementary Table 15. Prediction of gene structures of the *Ac. gramineus*, *Ac. calamus* A and *Ac. calamus* B genomes.

	Gene set		Number	Average gene length (bp)	Average CDS length (bp)	Average exon per gene	Average exon length (bp)	Average intron length (bp)
Ac. gramineus	De novo	AUGUSTUS	16,025	4,802.65	1,327.28	5.56	238.72	762.13
		GlimmerHMM	45,161	5,346.88	715.32	3.90	183.29	1,595.61
		Genscan	17,912	6,114.26	592.19	2.74	216.17	3,174.71
	Homolog	*A. shenzhenica*	25,611	3,424.25	876.79	3.98	220.21	854.39
		S. polyrhiza	25,577	3,443.01	862.50	4.02	214.45	853.94
		A. trichopoda	26,277	3,412.88	879.59	4.10	214.47	816.88
		Z. marina	24,161	3,523.82	870.06	4.17	208.43	836.02
		O. sativa	26,594	2,986.02	787.53	3.63	216.82	835.25
	RNA-seq		19,905	3,950.20	1,134.09	4.89	231.93	723.97
	CEGMA		422	5,958.62	1,203.15	8.30	145.02	651.77
	MAKER		24,803	5,424.41	1,085.00	5.21	259.96	965.36
	Final set		25,090	4,489.47	1,090.00	4.87	223.78	807.17
Ac. calamus A	De novo	AUGUSTUS	14,359	4,762.73	1,271.86	5.51	230.90	774.34
		GlimmerHMM	42,900	4,774.95	669.08	3.77	177.36	1,480.95
		Genscan	16,678	5,593.96	567.39	2.76	205.85	2,861.88
	Homolog	*A. shenzhenica*	22,311	3,710.87	859.89	3.96	217.03	962.49
		S. polyrhiza	22,150	3,590.69	846.56	4.02	210.85	910.15
		A. trichopoda	22,511	3,619.63	864.19	4.10	211.01	890.17
		Z. marina	20,884	3,903.69	857.63	4.16	206.00	962.97
		O. sativa	22,965	3,451.17	774.85	3.65	212.11	1,008.74
	RNA-seq		19,705	5,712.18	1,226.97	5.93	206.84	909.41
	CEGMA		410	6,270.36	1,193.80	8.28	144.21	697.52
	MAKER		22,450	5,642.51	991.08	5.24	241.64	1,031.02
	Final set		21,743	4,866.68	1,035.78	4.96	208.64	883.78
Ac. calamus B	De novo	AUGUSTUS	15,965	4,704.78	1,269.97	5.40	235.25	780.93
		GlimmerHMM	46,895	4,881.02	675.90	3.78	178.65	1,510.77
		Genscan	186,46	5,587.67	575.53	2.75	209.08	2,859.65
	Homolog	*A. shenzhenica*	24,429	3,851.02	865.00	3.90	221.72	1,029.18
		S. polyrhiza	24,630	3,877.72	844.85	3.93	215.07	1,035.71
		A. trichopoda	24,901	3,797.74	866.27	4.02	215.36	969.92
		Z. marina	23,200	3,815.26	850.29	4.06	209.25	967.84
		O. sativa	25,497	3,379.96	773.49	3.56	217.36	1,018.75
	RNA-seq		21,188	6,227.22	1,235.15	5.95	207.69	1,009.10
	CEGMA		422	6,111.20	1,176.04	8.23	142.86	682.38
	MAKER		24,707	5,713.09	1,005.90	5.19	244.39	1,060.45
	Final set		24,322	4,915.65	1,048.72	4.94	212.13	897.91

Supplementary Table 16. BUSCO assessment of genome annotation of *Ac. gramineus*, *Ac. calamus* A and *Ac. calamus* B genomes.

Genome	Class	Assembly	Percentage	Annotation	Percentage
Ac. gramineus	Complete BUSCOs	1,533	94.98%	1,406	87.11%
	Complete Single-Copy BUSCOs	1,450	89.84%	1,294	80.17%
	Complete Duplicated BUSCOs	83	5.14%	112	6.94%
	Fragmented BUSCOs	22	1.36%	108	6.69%
	Missing BUSCOs	59	3.65%	100	6.20%
	Total BUSCO groups searched	1,614	100%	1,614	100%
Ac. calamus	Complete BUSCOs	1525	94.48%	1450	89.84%
	Complete Single-Copy BUSCOs	552	34.20%	636	39.41%
	Complete Duplicated BUSCOs	973	60.29%	814	50.43%
	Fragmented BUSCOs	15	0.93%	95	5.89%
	Missing BUSCOs	74	4.58%	69	4.28%
	Total BUSCO groups searched	1614	100%	1614	100%
Ac. calamus A	Complete BUSCOs	1320	81.78%	1150	71.25%
	Complete Single-Copy BUSCOs	1279	79.24%	1087	67.35%
	Complete Duplicated BUSCOs	41	2.54%	63	3.90%
	Fragmented BUSCOs	52	3.22%	173	10.72%
	Missing BUSCOs	242	14.99%	291	18.03%
	Total BUSCO groups searched	1614	100%	1614	100%
Ac. calamus B	Complete BUSCOs	1417	87.79%	1245	77.14%
	Complete Single-Copy BUSCOs	1367	84.70%	1157	71.68%
	Complete Duplicated BUSCOs	50	3.10%	88	5.45%
	Fragmented BUSCOs	46	2.85%	172	10.66%
	Missing BUSCOs	151	9.36%	197	12.21%
	Total BUSCO groups searched	1614	100%	1614	100%

Supplementary Table 17. Non-coding RNA annotation results of *Ac. gramineus*, *Ac. calamus* A and *Ac. calamus* B genomes.

Species	Type		Copy(w)	Average length(bp)	Total length(bp)	% of genome
Ac. gramineus	miRNA		57	113.51	6,470	0.00
	tRNA		596	74.60	44,463	0.01
	rRNA	rRNA	159	526.40	83,697	0.02
		18S	42	1,567.17	65,821	0.02
		28S	36	196.61	7,078	0.00
		5.8S	39	157.85	6,156	0.00
		5S	42	110.52	4,642	0.00
		8S	0	0.00	0	0.00
	snRNA	snRNA	200	125.19	25,037	0.01
		CD-box	48	114.58	5,500	0.00
		HACA-box	12	130.17	1,562	0.00
		splicing	140	128.39	17,975	0.00
		scaRNA	0	0.00	0	0.00
Ac. calamus A	miRNA		44	122.39	5,385	0.00
	tRNA		481	74.92	36,038	0.01
	rRNA	rRNA	96	542.29	52,060	0.02
		18S	29	1,436.72	41,665	0.01
		28S	25	196.52	4,913	0.00
		5.8S	22	157.82	3,472	0.00
		5S	20	100.50	2,010	0.00
		8S	0	0.00	0	0.00
	snRNA	snRNA	170	124.57	21,177	0.01
		CD-box	45	124.71	5,612	0.00
		HACA-box	7	140.71	985	0.00
		splicing	118	123.56	14,580	0.00
		scaRNA	0	0.00	0	0.00
Ac. calamus B	miRNA		55	116.80	6,424	0.00
	tRNA		477	74.65	35,608	0.01
	rRNA	rRNA	38	127.87	4,859	0.00
		18S	1	598.00	598	0.00
		28S	1	186.00	186	0.00
		5.8S	0	0.00	0	0.00
		5S	36	113.19	4,075	0.00
		8S	0	0.00	0	0.00
	snRNA	snRNA	184	121.72	22,396	0.01
		CD-box	43	108.09	4,648	0.00
		HACA-box	8	141.25	1,130	0.00
		splicing	133	124.95	16,618	0.00
		scaRNA	0	0.00	0	0.00

Supplementary Table 18. Gene family clustering results of *Ac. gramineus*, *Ac. calamus* A, *Ac. calamus* B and other 16 species.

Species	Genes	Unclustered genes	Clustered genes	Families	Unique families	Unique families genes	Common families	Common families genes	Single copy	Average genes per family
Ac. calamus A	21,743	3,896	17,847	13,121	163	362	4,569	7,382	379	1.36
A. comosus	21,977	2,184	19,793	12,399	284	858	4,569	8,362	379	1.596
A. officinalis	26,005	2,936	23,069	11,821	531	3,051	4,569	8,406	379	1.952
A. shenzhenica	20,560	4,099	16,461	11,688	350	1,377	4,569	6,946	379	1.408
Ac. gramineus	25,090	3,807	21,283	14,243	261	686	4,569	8,220	379	1.494
A. thaliana	27,416	3,968	23,448	12,595	765	2,902	4,569	9,197	379	1.862
A. trichopoda	26,846	7,573	19,273	12,454	1,032	4,416	4,569	6,418	379	1.548
Ac. calamus B	24,322	4,600	19,722	13,802	208	463	4,569	7,878	379	1.429
B. distachyon	25,455	2,625	22,830	14,677	357	1,065	4,569	8,706	379	1.555
D. catenatum	26,791	5,586	21,205	13,362	721	3,217	4,569	7,573	379	1.587
M. acuminata	36,528	11,109	25,419	12,545	519	1,289	4,569	11,936	379	2.026
N. tetragona	31,589	7,724	23,865	12,018	994	4,835	4,569	7,357	379	1.986
O. sativa	27,694	6,993	20,701	13,927	556	1,396	4,569	7,934	379	1.486
P. dactylifera	25,377	2,385	22,992	12,565	224	577	4,569	10,302	379	1.83
P. equestris	26,471	6,985	19,486	13,174	675	2,281	4,569	7,304	379	1.479
P. trichocarpa	41,335	7,721	33,614	14,056	1175	3,685	4,569	12,686	379	2.391
S. bicolor	28,121	3,100	25,021	14,983	457	1,607	4,569	9,054	379	1.67
S. polyrhiza	19,623	3,429	16,194	11,395	360	1,378	4,569	6,903	379	1.421
V. vinifera	26,346	6,728	19,618	12,529	640	1,880	4,569	8,013	379	1.566

Supplementary Table 19. Clustered results of *Ac. gramineus*, *Ac. calamus* A, *Ac. calamus* B genes.

Species	Genes	Unclustered genes	Clustered genes	Families	Unique families	Unique families genes	Common families	Common families genes	Single copy	Average genes per family
Ac. gramineus	25,090	4,559	20,531	14,668	356	1,037	11,024	15,617	7,324	1.4
Ac. calamus A	21,743	4,450	17,293	13,527	196	439	11,024	14,310	7,324	1.278
Ac. calamus B	24,322	5,205	19,117	14,275	250	560	11,024	15,169	7,324	1.339

Supplementary Table 20. Summary of genome collinearity of *Ac. gramineus*, *Ac. calamus* A, *Ac. calamus* B.

Species1	Species2	Total genes	Synteny genes	Synteny genes ratio	Synteny genes pairs	Synteny blocks	Genes per Block
Ac. calamus A	*Ac. calamus* A	21,743	5,220	24.01	2,633	125	21.06
Ac. calamus A	*Ac. calamus* B	46,065	29,480	64	15,815	253	62.51
Ac. calamus A	*Ac. gramineus*	46,833	30,485	65.09	16,510	369	44.74
Ac. calamus B	*Ac. calamus* B	24,322	5,614	23.08	2,853	135	21.13
Ac. calamus B	*Ac. gramineus*	49,412	32,471	65.71	17,658	350	50.45
Ac. gramineus	*Ac. gramineus*	25,090	6,238	24.86	3,168	121	26.18

Supplementary Table 21. Homolog-specific bias in seven tissues.

Tissue	Total expressed gene pair	Total biased-expressed gene pair	A>B	A<B	Bias/Total (%)
Flower	11,713	3,785	1,608	2,177	32.31
Inflorescence	11,820	3,772	1,606	2,166	31.91
Peduncle	10,666	3,875	1,605	2,270	36.33
Bract	9,988	3,874	1,667	2,207	38.79
Leaf	10,049	3,747	1,638	2,109	37.29
Root	11,075	3,735	1,498	2,237	33.72
Stem	10,416	3,602	1,517	2,085	34.58

Note: A>B indicated biased toward subgenome A; B>A indicated biased toward subgenome B.

Supplementary Table 22. GO enrichment of expression biased gene in the subgenome A and B.

	GO	GO term	*p*-value
subgenome A	GO:0004143	diacylglycerol kinase activity	0.0006
	GO:0007205	protein kinase C-activating G-protein coupled receptor signaling pathway	0.0006
	GO:0008157	protein phosphatase 1 binding	0.0006
	GO:0008599	protein phosphatase type 1 regulator activity	0.0006
	GO:0046873	metal ion transmembrane transporter activity	0.0010
	GO:0006850	mitochondrial pyruvate transport	0.0018
	GO:0016992	lipoate synthase activity	0.0044
	GO:0006397	mRNA processing	0.0056
	GO:0051225	spindle assembly	0.0073
	GO:0070652	HAUS complex	0.0073
subgenome B	GO:0016866	intramolecular transferase activity	9.9101 e-05
	GO:0008152	metabolic process	0.0001
	GO:0009231	riboflavin biosynthetic process	0.0015
	GO:0008173	RNA methyltransferase activity	0.0031
	GO:0005840	ribosome	0.0065

Supplementary Table 23. The significant differences in the methylation levels of subgenome A and B of *Ac. calamus* in gene region and upstream and downstream 2kb region (Wilcoxon Rank Sum)**.**

	**Upstream region difference *p*-value**	**Gene region difference *p*-value**	**Downstream region difference *p*-value**
mCG	0.018737	0.000484	0.018737
mCHG	0.110329	0.04831	0.110329
mCHH	0.847587	0.704909	0.847587

Supplementary Table 24. Expression patter of genes related to vascular cambia and secondary cell wall formation in *Acorus.*

Gene	Gene ID	Root	Leaf	Stem
Ac. gramineus				
ARF5	DACA001086	1.37	0.06	2.46
WOX4	DACA006382	0	3.11	9.19
WOX10/14/13	DACA001393	0	1.77	1.27
	DACA009780	3.79	4.75	18.51
	DACA018367	0.57	0	0.25
	DACA018985	0	13.98	0
ARF7/19	DACA013540	22.42	1.93	10.68
PXY	DACA012469	0.99	0.24	30.44
ANT	DACA015996	1.55	0	10.52
	DACA022086	2.55	0.96	21.35
KNAT1	DACA015959	22.43	0	121.63
	DACA022105	0	0.27	72.14
PXL1/PXL2	DACA002255	14.64	0	3.25
Ac. calamus				
ARF5	CP_A007805	0.32	0.3	0.1
	CP_B002938	0.58	6.05	0.48
WOX4	CP_A013608	0	0	0.14
	CP_B006956	0.58	0	0.26
WOX10/14/13	CP_A000248	7.3	5.69	4.14
	CP_A008080	0.31	0	0.94
	CP_A011008	2.78	3.09	1.86
	CP_B002159	11.98	5.12	4
	CP_B014622	1.61	2.31	1.58
	CP_B002617	0	0	0.47
ARF7/19	CP_A005601	10.04	45.08	14.97
	CP_A008314	19.52	39.62	34.23
	CP_B011230	19.82	30.7	53.02
	CP_B022490	6.5	35.01	11.62
PXY	CP_B000326	7.9	1.12	12.31
	CP_A013984	0.18	0	0.7
	CP_A018971	0.18	0	0.15
	CP_B007217	0.48	0	1.39
ANT	CP_B013474	0.15	0.05	3.89
KNAT1	CP_A013965	0	0	95.94
	CP_A018934	29.04	0.12	38.85
	CP_B007234	0.07	0	25.97
	CP_B013439	41.6	0.29	42.4
PXL1/PXL2	CP_A010167	0.93	0.44	54.05
	CP_B015339	1.64	0.34	10.76

Supplementary Table 25. Gene number related to cotyledon development in angiosperms.

Gene	*A. thaliana*	*Ac. calamus* A	*Ac. calamus* B	*Ac. gramineus*	*O. sativa*	*Z. mays*	*N. colorata*	*A. trichopoda*
STM	1	2	2	3	0	0	1	1
PNH	1	3	2	2	1	2	2	2
PIN1	1	2	2	2	2	3	2	1
PID	1	2	2	2	2	2	1	1
CUC (1/2/3)	3(1/1/1)	2 (0/1/1)	2 (0/1/1)	2 (0/1/1)	2 (0/1/1)	2 (0/1/1)	3 (0/2/1)	3 (0/2/1)

Supplementary Table 26. Genome content of *Ac. calamus* A and *Ac. calamus* B.

	***Ac. calamus* A**		***Ac. calamus* B**	
	**Size (Mb)**	**Percent (%)**	**Size (Mb)**	**Percent (%)**
Exon	25.83	8.1	29.13	8.07
Intron	79.99	25.09	90.43	25.07
Copia	19.72	6.18	22.35	6.2
Gypsy	47.16	14.79	52.22	14.47
Other LTR	10.2	3.2	11.79	3.27
Other TE	40.89	12.82	47.12	13.06
ncRNA	0.07	0.02	0.03	0.01
N	0.36	0.11	0.41	0.11
Undetermined	94.65	29.69	107.31	29.74
Total	318.86	-	360.79	-

Supplementary Table 27. Lost genes GO enrichment in subgenome A of *Ac. calamus*.

	**GO**	**GO term**	***p*-value**
subgenome A	GO:0004883	glucocorticoid receptor activity	0.0081
	GO:0042921	glucocorticoid receptor signaling pathway	0.0081
	GO:0043402	glucocorticoid mediated signaling pathway	0.0081

Supplementary Table 28. Lost genes GO enrichment in subgenome B of *Ac. calamus*.

	GO	GO term	*p*-value
subgenome B	GO:0043461	proton-transporting ATP synthase complex assembly	0.0030
	GO:0030599	pectinesterase activity	0.0044
	GO:0000154	rRNA modification	0.0086
	GO:0000179	rRNA (adenine-N6, N6-)-dimethyltransferase activity	0.0086

Supplementary references

1. Ranallo-Benavidez, T.R., Jaron, K.S. & Schatz, M.C. GenomeScope 2.0 and Smudgeplot for reference-free profiling of polyploid genomes. *Nat. Commun.* **11**, 1432 (2020).
2. Vurture, G. W. *et al.* GenomeScope: fast reference-free genome profiling from short reads. *Bioinformatics* **33**, 2202–2204 (2017).
3. Jiao, Y., Paterson, A. H. Polyploidy-associated genome modifications during land plant evolution. *Philos. Trans. R. Soc. Lond., B, Biol. Sci.* **369**, 20130355 (2014).
4. Edger, P. P., McKain, M. R., Bird, K. A. & VanBuren, R. Subgenome assignment in allopolyploids: challenges and future directions. *Curr. Opin. Plant Biol.* **42**, 76–80 (2018).
5. Cheng, F. *et al*. Gene retention, fractionation and subgenome differences in polyploid plants. *Nat. Plants* **4**, 258–268 (2018).
