# Supplementary material for: Diploid and tetraploid genomes of Acorus and the evolution of monocots
Source: Nat Commun. 2023 Jun 20;14:3661. doi: 10.1038/s41467-023-38829-3 (PMC10282084; doi:10.1038/s41467-023-38829-3)
Supplement: Supplementary file 3 — Description of Additional Supplementary Files [file 41467_2023_38829_MOESM3_ESM.pdf]

Description of Additional Supplementary Files

Supplementary Data 1. GO enrichment for significantly contracted gene families.

Supplementary Data 2. GO enrichment for significantly expanded gene families.

Supplementary Data 3. KEGG pathways for significantly contracted gene families.

Supplementary Data 4. KEGG pathways for significantly expanded gene families.

Supplementary Data 5. Unique gene families of monocots.

Supplementary Data 6. Selection pressure on the 808 homologs showing an extreme divergent expression in two subgenomes.

Supplementary Data 7. List of MADS-box genes identified in *Ac. gramineus*.

Supplementary Data 8. List of MADS-box genes identified in *Ac. calamus*.

Supplementary Data 9. Genes and their functions related to vascular cambia and secondary xylem development in *Arabidopsis*.

Supplementary Data 10. Gene IDs for genes related to vascular cambia and secondary xylem development identified in angiosperms.

Supplementary Data 11. Gene number related to vascular cambia and secondary cell wall formation in angiosperms.

Supplementary Data 12. List of genes related to cotyledon development identified in eight species.

Supplementary Data 13. Presence/absence analysis of known plant immunity components in angiosperms.

Supplementary Data 14. Statistics of expression bias patterns among seven tissues of *Ac. calamus*.

Supplementary Data 15. Lost genes ID in subgenome A or B of *Ac. calamus*.
