# Supplementary material for: Diploid and tetraploid genomes of Acorus and the evolution of monocots
Source: Nat Commun. 2023 Jun 20;14:3661. doi: 10.1038/s41467-023-38829-3 (PMC10282084; doi:10.1038/s41467-023-38829-3)
Supplement: Supplementary file 19 — Reporting Summary [file 41467_2023_38829_MOESM19_ESM.pdf]

nature portfolio

Corresponding author(s): Zhong-Jian Liu

Last updated by author(s): Apr 17, 2023

Reporting Summary

Nature Portfolio wishes to improve the reproducibility of the work that we publish. This form provides structure for consistency and transparency in reporting. For further information on Nature Portfolio policies, see our Editorial Policies and the Editorial Policy Checklist.

Statistics

For all statistical analyses, confirm that the following items are present in the figure legend, table legend, main text, or Methods section.

n/a	Confirmed	
☐	☒	The exact sample size (*n*) for each experimental group/condition, given as a discrete number and unit of measurement
☐	☒	A statement on whether measurements were taken from distinct samples or whether the same sample was measured repeatedly
☐	☒	The statistical test(s) used AND whether they are one- or two-sided<br>*Only common tests should be described solely by name; describe more complex techniques in the Methods section.*
☒	☐	A description of all covariates tested
☒	☐	A description of any assumptions or corrections, such as tests of normality and adjustment for multiple comparisons
☐	☒	A full description of the statistical parameters including central tendency (e.g. means) or other basic estimates (e.g. regression coefficient) AND variation (e.g. standard deviation) or associated estimates of uncertainty (e.g. confidence intervals)
☐	☒	For null hypothesis testing, the test statistic (e.g. *F*, *t*, *r*) with confidence intervals, effect sizes, degrees of freedom and *P* value noted<br>*Give P values as exact values whenever suitable.*
☐	☒	For Bayesian analysis, information on the choice of priors and Markov chain Monte Carlo settings
☒	☐	For hierarchical and complex designs, identification of the appropriate level for tests and full reporting of outcomes
☒	☐	Estimates of effect sizes (e.g. Cohen's *d*, Pearson's *r*), indicating how they were calculated

Our web collection on statistics for biologists contains articles on many of the points above.

Software and code

Policy information about availability of computer code

Data collection

PacBio CCS (Circular Consensus Sequencing) reads of genome data were sequenced by a 20 kb single-molecule real-time (SMRT) DNA library on the PacBio Sequel platform, Illumina data of genome data were accquired from the Illumina HiSeq 4000 platform. Hi-C reads ware sequenced on the Illumina HiSeq X platform. Raw reads of transcriptome were generated by the Illumina platform.

Data analysis

The software used in this study have been described in the methods. The softwares are listed as follows: Jellyfish v2.1.4, GenomeScope2, Falcon, Smartdenovo v1.0, Pilon v1.22, HiC-Pro v.2.8.0, SOAPnuke v1.5.3, 3d-DNA (v 180922), Juicerbox (v1.11.08), GeneWise v.2.4.1, Augustus v.2.7, GlimmerHMM v.3.02, SNAP (version 2006-07-28), TopHat v2.1.1, Cufflinks v2.1.1, MAKER v.1.0, InterProScan v.4.8, tRNAscan-SE, INFERNAL, RepeatProteinMask v.4.1.0, LTR_FINDER v.1.0.2, PILER v.1.3.4, RepeatModeler v.1.0.3, TRF (v4.09), cluster v3.0, SubPhaser, OrthoMCL v2.0.9, i-ADHoRe (v3.0), PAML v4.9, MUSCL v3.8.31, Gblocks, MrBayes, LAST, MCSCANX, ClustalW, MEGA5, Trinity v.2.4.8.

For manuscripts utilizing custom algorithms or software that are central to the research but not yet described in published literature, software must be made available to editors and reviewers. We strongly encourage code deposition in a community repository (e.g. GitHub). See the Nature Portfolio guidelines for submitting code & software for further information.

Data

Policy information about availability of data

All manuscripts must include a data availability statement. This statement should provide the following information, where applicable:

- Accession codes, unique identifiers, or web links for publicly available datasets
- A description of any restrictions on data availability
- For clinical datasets or third party data, please ensure that the statement adheres to our policy

Genome sequences and whole-genome assemblies have been submitted to the National Centre for Biotechnology Information (NCBI) database under PRJNA782402.

Field-specific reporting

Please select the one below that is the best fit for your research. If you are not sure, read the appropriate sections before making your selection.

☒ Life sciences ☐ Behavioural & social sciences ☐ Ecological, evolutionary & environmental sciences

For a reference copy of the document with all sections, see nature.com/documents/nr-reporting-summary-flat.pdf

Life sciences study design

All studies must disclose on these points even when the disclosure is negative.

Sample size	Sample of Ac. gramineus and Ac. calamus were selected from wild accessions to investigate meaningful results.
Data exclusions	No data exclusions in this manuscript.
Replication	Three replicates were performed for each sample RNA sequencing. Three replicates in the flow cytometry and fluorescence in situ hybridization analysis with similar results.
Randomization	No randomization in this manuscript as samples were not allocated into experimental groups.
Blinding	No blinding in this manuscript as the data were not allocated into groups

Reporting for specific materials, systems and methods

We require information from authors about some types of materials, experimental systems and methods used in many studies. Here, indicate whether each material, system or method listed is relevant to your study. If you are not sure if a list item applies to your research, read the appropriate section before selecting a response.

Materials & experimental systems

n/a	Involved in the study	
☒	☐	Antibodies
☒	☐	Eukaryotic cell lines
☒	☐	Palaeontology and archaeology
☒	☐	Animals and other organisms
☒	☐	Human research participants
☒	☐	Clinical data
☒	☐	Dual use research of concern

Methods

n/a	Involved in the study	
☒	☐	ChIP-seq
☐	☒	Flow cytometry
☒	☐	MRI-based neuroimaging

Flow Cytometry

Plots

Confirm that:

☒ The axis labels state the marker and fluorochrome used (e.g. CD4-FITC).

☒ The axis scales are clearly visible. Include numbers along axes only for bottom left plot of group (a 'group' is an analysis of identical markers).

☒ All plots are contour plots with outliers or pseudocolor plots.

☒ A numerical value for number of cells or percentage (with statistics) is provided.

Methodology

Sample preparation	The flesh leaves of Ac. gramineus and Ac. calamus were collected for analysis.
Instrument	Firstly, the cell nucleus is dissociated, and then the bases on the chromosomes of the cell nucleus are stained with a fluorescent dye solution, and then the fluorescence intensity emitted by the stained bases in the nucleus is detected by an instrument. The abscissa of the result is the fluorescence intensity, and the ordinate is the number of cells. The fluorescence intensity is directly proportional to the DNA content, the genome size of the sample can be calculated from the genome size of the control sample.
Software	The flow cytometry was analysis by the Sysmex Partec CyFlow Cube6.
Cell population abundance	N/A
Gating strategy	N/A

☐ Tick this box to confirm that a figure exemplifying the gating strategy is provided in the Supplementary Information.
