# Supplementary material for: Diploid and tetraploid genomes of Acorus and the evolution of monocots
Source: Nat Commun. 2023 Jun 20;14:3661. doi: 10.1038/s41467-023-38829-3 (PMC10282084; doi:10.1038/s41467-023-38829-3)
Supplement: Supplementary file 2 — Peer Review File [file 41467_2023_38829_MOESM2_ESM.pdf]

nature portfolio

Peer Review File

Diploid and tetraploid genomes of Acorus and the evolution of monocots

Open Access This file is licensed under a Creative Commons Attribution 4.0 International License, which permits use, sharing, adaptation, distribution and reproduction in any medium or format, as long as you give appropriate credit to the original author(s) and the source, provide a link to the Creative Commons license, and indicate if changes were made. In the cases where the authors are anonymous, such as is the case for the reports of anonymous peer reviewers, author attribution should be to 'Anonymous Referee' followed by a clear attribution to the source work. The images or other third party material in this file are included in the article's Creative Commons license, unless indicated otherwise in a credit line to the material. If material is not included in the article's Creative Commons license and your intended use is not permitted by statutory regulation or exceeds the permitted use, you will need to obtain permission directly from the copyright holder. To view a copy of this license, visit http://creativecommons.org/licenses/by/4.0/.

Editorial Note: This manuscript has been previously reviewed at another journal that is not operating a transparent peer review scheme. The manuscript was considered suitable for publication without further review at Nature Communications.

Reviewers' Comments:

Reviewer #2:
Remarks to the Author:
This is the manuscript I have reviewed for Nature Plants a while ago. The authors reported two genomes of Acorus species (A. gramineus and A. calamus) which are the only two species from the Acoraceae family. This lineage belongs to the earliest branching species among monocots and is phylogenetically critical for understanding the evolutionary history of monocots. The authors found two Acorus species shared an ancient whole genome duplication event, but they didn't share the tau WGD. Given the A. calamus is a tetraploid, they separated the two subgenomes and investigated the divergence timing of the formation of the tetraploid, and the subgenome dominance pattern. They also reconstructed an ancestral monocot karyotype and proposed the karyotype evolution following the diversification of monocots. Several genomic changes were proposed to be likely associated with the specific traits.

I have raised several concerns and questions previously. After reading this new version again, I think the authors have addressed these comments carefully, and they also have added many details regarding the genome assembly and QC. Therefore, I actually have no further concerns now.

After reading the comments from other reviewers, I feel the authors have done a good job in response and revising the manuscript, and I think it is suitable for publication. I can only find two requests that still need more attention:

1)In the response to comment 1 of reviewer #1, the authors claimed that they have provided the in-house scripts for other analyses, such as get_methylation_gene.py, cal_methylation_distribution_in_genic_region.py, and allelic_gene_expression_compare.py. However, they might have forgotten to include these scripts in the Supplementary Code.

2)In the response to comment 15 of reviewer #4, the authors have explained how they run the data, but didn't answer the reviewer's concern about "How are the potential problem reads mappings removed?". I would suggest the authors to rerun the Hi-C Mapping using the reviewers' suggestion("specifically the -5SP options; see https://phasegenomics.github.io/2019/09/19/hic-alignment-and-qc.html") to see any difference.

Reviewer #3:
Remarks to the Author:
I reviewed the previous versions of this manuscript twice elsewhere. Because this is a new submission to a different journal, I went through the main text as I did a newly assigned manuscript. To repeat my comment in the first-round review, I think their genomic data are of great value because of the unique phylogenetic position of Acorus being sister to the rest of the extant monocots. And I thank the authors for addressing some of my earlier concerns. However, I regret that the previous rounds of revisions may not have improved the manuscript as efficiently as one might expect. I agree with Reviewer 1 that the authors tended to edit particular points rather than throughout the manuscript, even with a clear note that we only provided a few examples among many similar problems, and the

problem seems to persist in several points still. If all the problems already identified have been resolved, it should greatly improve the readability of the manuscript. I encourage the authors to go over the past reviewer comments, as well as comments in this round of review, and revise the manuscript further to fix the remaining problems. This time, I tried to list as many similar problems as possible, but I admit that the lists could not be completed in my limited time.

One good example of the above problem is the points I raised on the syntenic dot plot. My comments in the first- and second-round reviews were:

>Fig. 3b: The resolution of dot plots is not enough to judge it, but in A. calamus, it looks to me that the top right and bottom left circles mark 2 distinct synteny blocks spanning 2 chromosomes with different Ks values, and if so, this is inappropriate as an example for a recent duplication. Also, arrowheads may be better removed if the arrow orientation has no associated information. This panel will be easier to understand if chromosome numbers are labeled.

>Comment 22. Thank you for your response. I was surprised that, contrary to your statement that the revision is done, nothing seems to have been changed except for the slight change of the resolution.

These points, except the resolution issue (enlarged images are now included in Supp Fig. 20), have not been addressed even after repeated revisions. I find that, in their response letter, the authors often state that they have addressed the problem, but it actually is not enough in many cases. If there is a justification, the authors are of course entitled not to revise, but the reason should be clarified.

Similar problems seem prevalent in their responses to other reviewers as well. For example, in response to Reviewer 1's concern about the availability of reproducible code, they stated that they included their in-house codes, such as get_methylation_gene.py, cal_methylation_distribution_in_genic_region.py, and allelic_gene_expression_compare.py. However, I could not locate any of those codes in the submitted files and in figshare. There are 5 files identified as Supplementary Codes, but none of them match to the above-mentioned codes. Looking into it, they seem to be small codes they used in the k-mer analysis, and I am afraid that those codes do not grant the reproducibility of their analyses presented in this complex paper.

In this version, the authors updated or added many data. Modified or newly added texts are often incorrect, or very difficult to parse, in terms of sentence structure and punctuation. Examples include: "Color bar, Z-score (frequency), the count (Z-score) of subgenome-specific 13-mer." in the legend of Fig. 1, "the homologous based repeats" in L171, and the sentence structure of L306-309.

Also, the newly presented data tended to be not fully explained and tended to repeat the mistakes that have already been pointed out elsewhere in the previous rounds of reviews. I should mention that insufficient labeling was among the points I have raised since the first revision.

Many figures have short, insufficient legends and very brief mentions in the main text, making it extremely difficult to understand the validity of data. Examples include, but may not be limited to: Extended Data Figure 2, Extended Data Figure 3, Extended Data Figure 5, Extended Data Figure 6, Supplementary Figure 19. For example (just as an example. It applies to the others too, and I expect the others to be addressed as well), the authors referenced Extended Data Figure 2 in the main text with the claim that putative centromeres may be detected in 16 and 6 chromosomes in A. calamus and A. gramineus, without indicating which of the illustrated peaks they think corresponds to the centromeres. The authors should clearly indicate what data they are discussing, rather than letting readers chase the authors' logic without sufficient navigation. I should point out that I raised the same concern on another figure (k-mer freq distribution) in earlier rounds of reviews, and I hope that this

specific example of Extended Data Figure 2, as well as similar problems in the other figures, which may be listed above, will be fixed in the next version.

Labels of many figures are not readable because of low image resolution. Examples include, but may not be limited to: Fig. 4d, Fig. 5, Extended Data Figure 2, Extended Data Figure 5, Supp Fig. 6, Extended Data Figure 2.

All Figs should be self-contained, but labels and legends are missing in many figures. Examples include, but may not be limited to: X tick labels in Fig. 4c, colorbar in Fig. 4d, colors in Extended Data Figure 6, Y axis label of Extended Data Figure 7, colorbars in Extended Data Figure 8, colorbars in Extended Data Figure 10, support values and bars in Extended Data Figure 3, notations in Extended Data Figure 5d-f, dashed lines in Extended Data Figure 4, colorbar in Supplementary Figure 22, colorbar in Supplementary Figure 24.

Abbreviations are not defined in many figures. Examples include, but may not be limited to: X tick labels of Extended Data Figure 10.

Fig. 2. Green and red in small pie charts are not explained. I assume they represent expanded/contracted gene families, but figure legends should be self-contained without ambiguity. I noticed that the authors correctly explained it in the response letter to Reviewer 2, but did not revise the legend so.

Extended Data Figure 3: Some species have dashed lines, but others do not. Please explain what it indicates. Also, please define the unit of scale bars. Please define the values around branches. Is there any reason to show branch lengths in coalescence-based trees but not in concatenation-based trees?

Extended Data Figure 7: What does the dashed line indicate?

Extended Data Figure 7: I failed to understand what this figure shows. Legends say the data are from pairwise comparisons, but the labels in the figure indicate only one species, not pairs. Could you clarify more?

Supplementary Figure 1: Please add a scale bar if available.

L73 "mosaic genomic features, important for...": could be "likely important for", as provided evidence is only gene expression, which may or may not correlate with phenotypes.

L386 "most genes having evidence for purifying selection were located on subgenome B ...(Figure 4c).": From what I can see in Fig. 4c, there is very little difference between subgenomes A and B, and most genes in both subgenomes seem to be under purifying selection, given the Ka/Ks distribution being mostly below 1.0. The authors should elaborate on the logic behind their claim, preferably with appropriate statistical testing.

L752 "the file of main result was k13_q50_f2.bin.enrich; The file with concrete results is k13_q50_f2.bin.enrich.": Are you providing two sets of results for different analyses? I was confused by how "main result" and "concrete results" could be different, and further by that the same file name was referred to in both cases.

L767 "the most poorly aligned sequences": Could you provide a quantitative threshold?

L782 "T=Ks / 2r.": r should be defined.

L793 "GTR + gamma model": The gamma model should be defined in terms of the number of site-wise rate categories.

L821 P < 0.01 and FDR < 0.05": I failed to understand why significance thresholds are set for both unadjusted P and FDR. Could you clarify the logic behind the design of the analysis?

L823: dot-"p"lot?

The authors added RNA-seq data for floral organs in the two Acorus species without sample information (e.g., developmental stages). Appropriate descriptions, as well as photographs of flower anatomy (to show how the authors dissected samples), should be provided. This seems to be the case in other samples as well. For example, How Flo-1 to Flo-3 are staged does not seem to be explained at all.

To be clear, many of the above points are explained with a few examples among many similar problems. I expect that all similar cases for individual concerns will be identified throughout the manuscript by the authors, and corrections will be made even though some of them are not individually mentioned in the examples. It is important for the authors to thoroughly address all of these issues in order to improve the readability and clarity of the manuscript.

Response to Reviewer Comments

Reviewer #2 (Remarks to the Author):

This is the manuscript I have reviewed for Nature Plants a while ago. The authors reported two genomes of Acorus species (A. gramineus and A. calamus) which are the only two species from the Acoraceae family. This lineage belongs to the earliest branching species among monocots and is phylogenetically critical for understanding the evolutionary history of monocots. The authors found two Acorus species shared an ancient whole genome duplication event, but they didn't share the tau WGD. Given the A. calamus is a tetraploid, they separated the two subgenomes and investigated the divergence timing of the formation of the tetraploid, and the subgenome dominance pattern. They also reconstructed an ancestral monocot karyotype and proposed the karyotype evolution following the diversification of monocots. Several genomic changes were proposed to be likely associated with the specific traits.

I have raised several concerns and questions previously. After reading this new version again, I think the authors have addressed these comments carefully, and they also have added many details regarding the genome assembly and QC. Therefore, I actually have no further concerns now.

After reading the comments from other reviewers, I feel the authors have done a good job in response and revising the manuscript, and I think it is suitable for publication. I can only find two requests that still need more attention:

Comment 1.

1)In the response to comment 1 of reviewer #1, the authors claimed that they have provided the in-house scripts for other analyses, such as get_methylation_gene.py, cal_methylation_distribution_in_genic_region.py, and allelic_gene_expression_compare.py. However, they might have forgotten to include these scripts in the Supplementary Code.

Response: Thanks for your advices. We have added the codes, allelic_gene_expression_compare.py (**Supplementary Code 6**), cal_methylation_distribution_in_genic_region.py (**Supplementary Code 7**), get_methylation_gene.py (**Supplementary Code 8**) into the **Supplementary Codes** (please see **Supplementary Codes 6-8**).

Comment 2.

2)In the response to comment 15 of reviewer #4, the authors have explained how they run the data, but didn't answer the reviewer's concern about "How are the potential problem reads mappings removed?". I would suggest the authors to rerun the Hi-C Mapping using the reviewers' suggestion ("specifically the -5SP options; see https://phasegenomics.github.io/2019/09/19/hic-alignment-and-qc.html") to see any difference.

Response: According to the suggested parameters, we realigned the Hi-C data to the *Acorus* assembly to detect assembly errors. First, we used the command "bwa index -a bwtsw [genome.fasta]" to build the genome index. Then, we used the command "bwa mem -5SP genome.fasta [fwd_hic.fastq] [rev_hic.fastq] | samtools view -S -h -b -F 2316 > [aligned.bam]" to align the Hi-C data and filter the alignment results (https://phasegenomics.github.io/2019/09/19/hic-alignment-and-qc.html). Next, we used the Juicer software to generate contact signal matrices and manually checked the assembly results using the visualization software Juicebox. We found no problems with the previous assembly results. Finally, we used the imshow function in the matplotlib package to draw the Hi-C heatmap, as shown below, which are consistent with previous results.

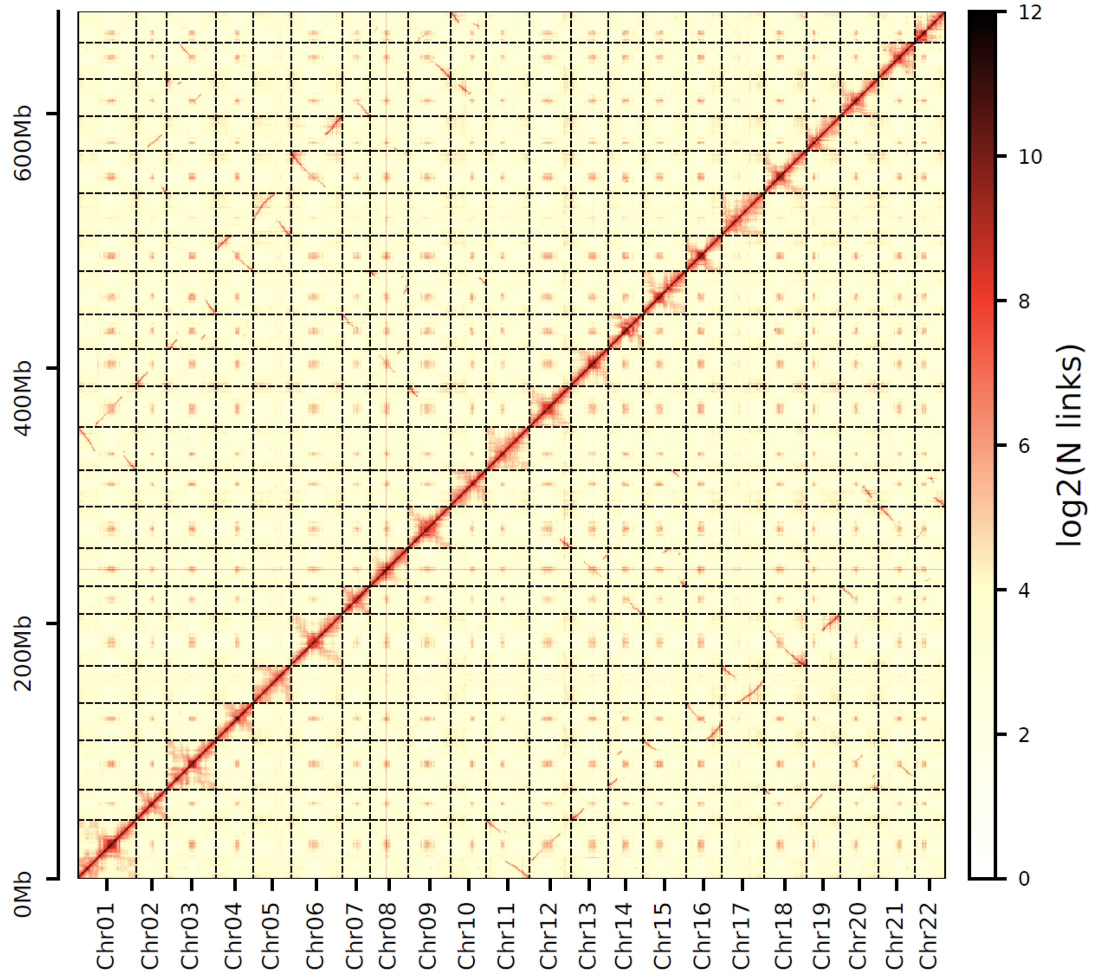

Reviewer #3 (Remarks to the Author):

I reviewed the previous versions of this manuscript twice elsewhere. Because this is a new submission to a different journal, I went through the main text as I did a newly assigned manuscript. To repeat my comment in the first-round review, I think their genomic data are of great value because of the unique phylogenetic position of Acorus being sister to the rest of the extant monocots. And I thank the authors for addressing some of my earlier concerns. However, I regret that the previous rounds of revisions may not have improved the manuscript as efficiently as one might expect. I agree with Reviewer 1 that the authors tended to edit particular points rather than throughout the manuscript, even with a clear note that we only provided a few examples among many similar problems, and the problem seems to persist in several points still. If all the problems already identified have been resolved, it should greatly improve the readability of the manuscript. I encourage the authors to go over the past reviewer comments, as well as comments in this round of review, and revise the manuscript further to fix the remaining problems. This time, I tried to list as many similar problems as possible, but I admit that the lists could not be completed in my limited time.

One good example of the above problem is the points I raised on the syntenic dot plot. My comments in the first- and second-round reviews were:

Comment 1.
>Fig. 3b: The resolution of dot plots is not enough to judge it, but in A. calamus, it looks to me that the top right and bottom left circles mark 2 distinct synteny blocks spanning 2 chromosomes with different Ks values, and if so, this is inappropriate as an example for a recent duplication. Also, arrowheads may be better removed if the arrow orientation has no associated information. This panel will be easier to understand if chromosome numbers are labeled.
Response: Thanks for your professional and helpful comments! I'm sorry we've been ignoring this problem. We modified the region circled in the dot plot of *Ac. calamus*, removed arrows and added chromosome numbers in Fig. 3b. Meanwhile, we enlarged the scale of the picture for easy viewing.

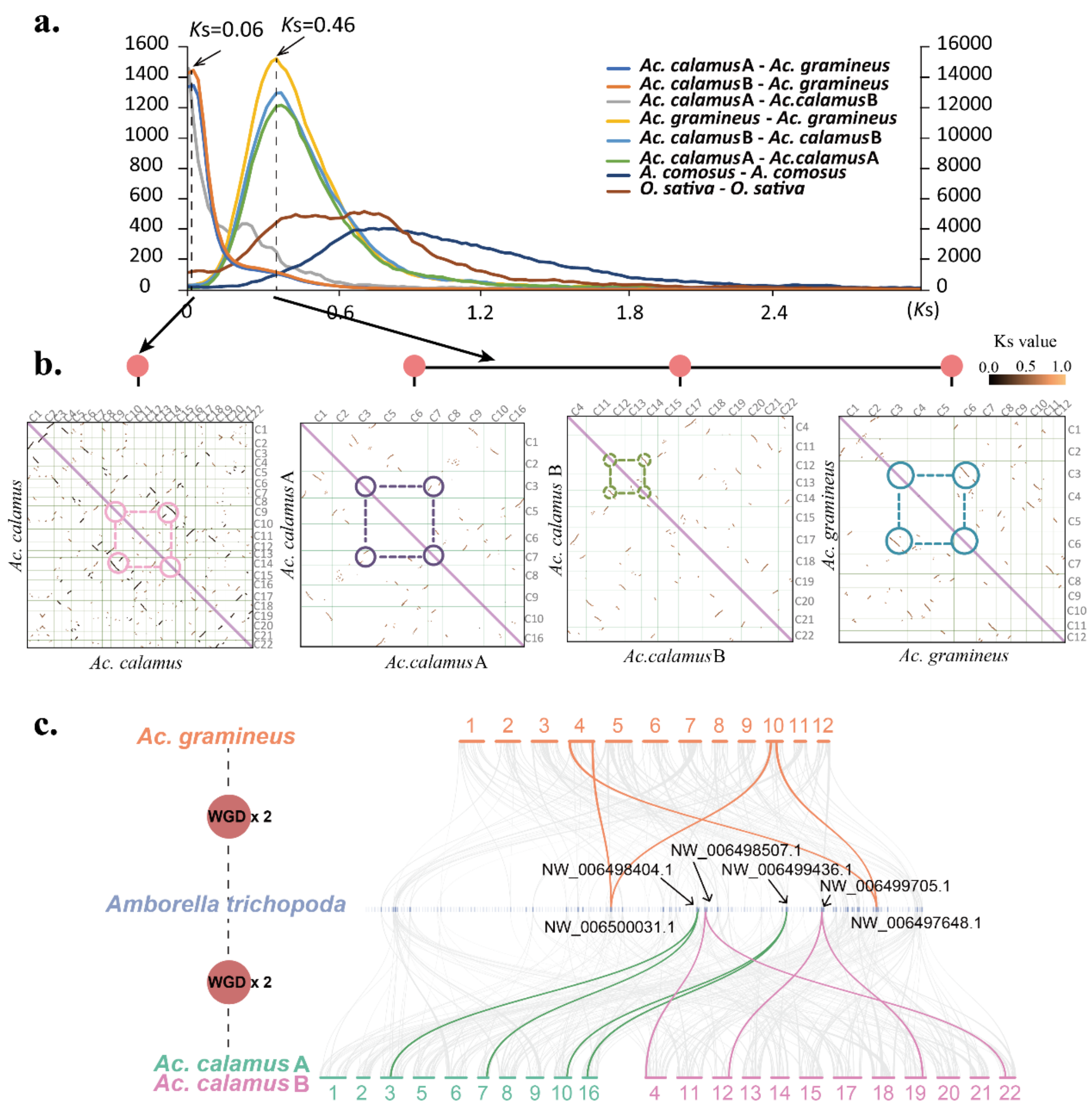

Comment 2

>Comment 22. Thank you for your response. I was surprised that, contrary to your statement that the revision is done, nothing seems to have been changed except for the slight change of the resolution.

Response: Thank you for your advices. We have removed the inaccurate sentence "it's an allotetraploid due to different numbers and size of the chromosomes". Based on the results from genomescope2 and smudgeplot, we have confirmed that *Acorus* is an allopolyploid. Then, we used subphaser to cluster *Acorus* chromosomes into two groups, the results indicated significant differences between its subgenomes. This further supports the fact that *Acorus* is an allopolyploid. As mentioned in the subphaser article, the tool is unable to distinguish between homologous polyploids but works well on neoallopolyploids and homoploid hybrids containing subgenome-specific sequences like wheat, whereas it fails on autopolyploids lacking subgenome-specific sequences like alfalfa.

Comment 3.

These points, except the resolution issue (enlarged images are now included in Supp Fig. 20), have not been addressed even after repeated revisions. I find that, in their response letter, the authors often state that they have addressed the problem, but it actually is not enough in many cases. If there is a justification, the authors are of course entitled not to revise, but the reason should be clarified.

Response: Thank you for your valuable advice! I'm sorry that we have been ignoring this problem. We have modified the circled area in the dot plot of *Ac. calamus*. And we removed all arrows and added chromosome numbers in Supp Fig. 20. Meanwhile, we updated the legend for better understanding.

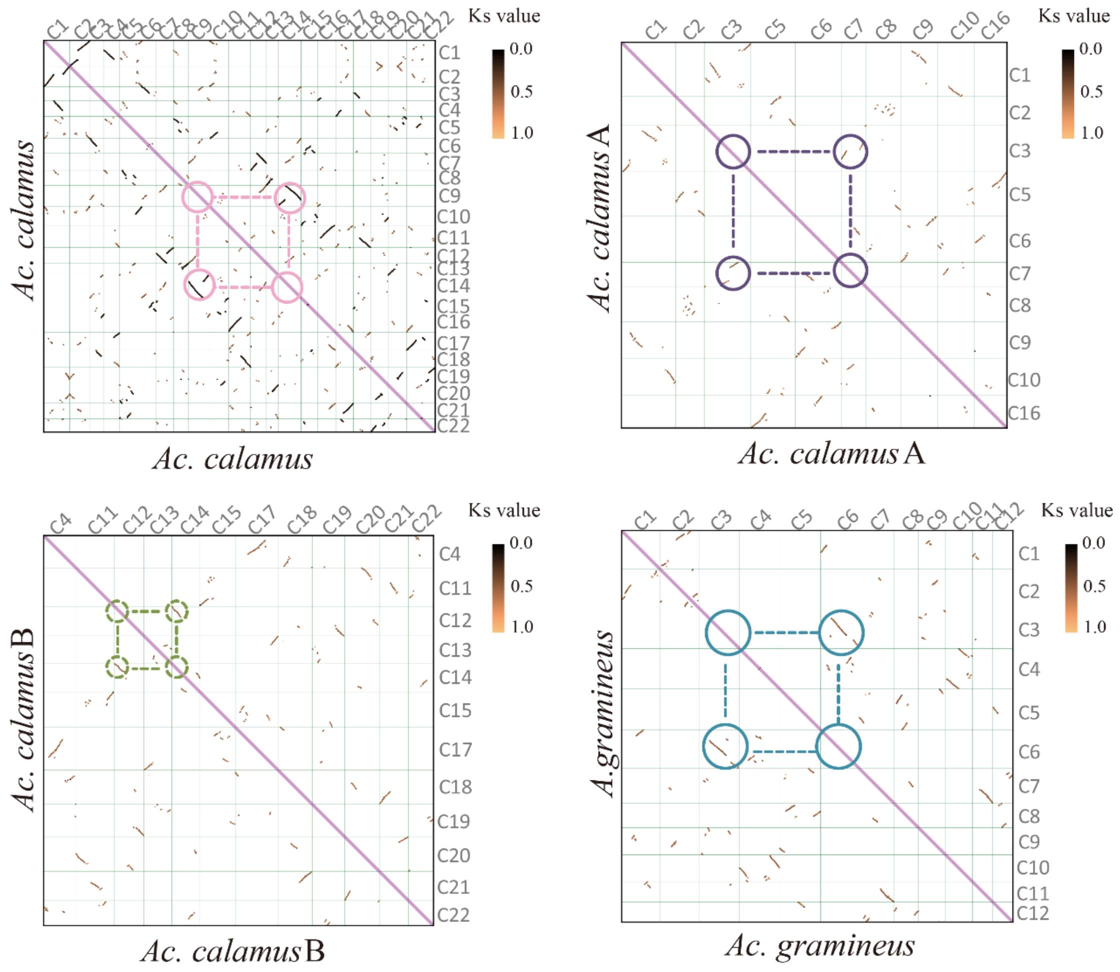

Supplementary Figure 20. Dot plots of paralogues with Ks coloration in the *Ac. calamus* A (3-7 chromosomal relationships in purple circles), *Ac. calamus* B (12-14 chromosomal relationships in green circles) and *Ac. gramineus* (3-6 chromosomal relationships in blue circles) genomes illustrating the shared WGD and paralogues in the *Ac. calamus* genome (9-14 chromosomal relationships in pink circles) clarifying the independent WGD event of *Ac. calamus*. The syntenic gene pairs are colored by the synonymous mutation values.

Comment 4.

Similar problems seem prevalent in their responses to other reviewers as well. For example, in response to Reviewer 1's concern about the availability of reproducible code, they stated that they included their in-house codes, such as get_methylation_gene.py, cal_methylation_distribution_in_genic_region.py, and allelic_gene_expression_compare.py. However, I could not locate any of those codes in the submitted files and in figshare. There are 5 files identified as Supplementary Codes, but none of them match to the above-mentioned codes. Looking into it, they seem to be small codes they used in the k-mer analysis, and I am afraid that those codes do not grant the reproducibility of their analyses presented in this complex paper.

Response: Sorry! We have ignored Codes 6–8**.** Thank you for your advice. We have added Codes 6–8, allelic_gene_expression_compare.py (**Supplementary Code 6**), cal_methylation_distribution_in_genic_region.py (**Supplementary Code 7**), get_methylation_gene.py (**Supplementary Code 8**) into the **Supplementary Codes** (please see **Supplementary Codes 6–8**).

Comment 5.

In this version, the authors updated or added many data. Modified or newly added texts are often incorrect, or very difficult to parse, in terms of sentence structure and punctuation. Examples include: "Color bar, Z-score (frequency), the count (Z-score) of subgenome-specific 13-mer." in the legend of Fig. 1, "the homologous based repeats" in L171, and the sentence structure of L306-309.

Response: Thanks for your excellent advice. We mainly added the results of the ploidy analysis in *Ac. calamus* and *Ac. gramineus* using Genomescope2, Smudgeplot and Subphaser, and updated previous analysis results. We revised the terms of sentence structure and punctuation. The main revised results are as follows.

(1) We have Modified the legend of **Figure 1** as follows.

Figure 1. **Distinguishing allo-subgenomes by collinear Hi-C signal and subgenome reconstruction for *Ac. calamus***. a. Distinguishing allo-subgenomes by collinear Hi-C signal.

We used Illumina sequencing reads from Hi-C libraries to reconstruct physical maps by ordering and clustering the assembled scaffolds into 22 pseudomolecules in the haploid genome of *Ac. calamus*. The vertical color bar on the right of the axis indicates the logarithm (log2) of chromatin contact frequency. B. Subgenome construction (see **Methods**). The 22 pseudomolecules of *Ac. calamus* were divided into two subgenomes, subgenome A including Chr01, 02, 03, 05-10 and 16, and subgenome B including Chr04, 11-15 and 17-22 (**Supplementary Table 6**). The color bar at the top indicates the Z-scaled relative abundance of *k*-mers, the larger the Z score, the higher the relative abundance of a *k*-mer.

(2) We also replaced "the homologous based repeats" to "the predicted repeats based on homology". Please see Page 7 Line 161.

(3) We also have modified the following content: "Chr11 of *Ac. calamus* B shares good collinearity with A1-1 and A1-3 Chr1 of *Ac. calamus* A and was aligned to G4-1 and G4-3 on chromosome 4 and G1-4 on Chr1 of *Ac. gramineus*, suggesting either Chr11 of *Ac. calamus* B or Chr1 of *Ac. calamus* A retained the ancestral karyotype. We further compared Chr1 of *Ac. calamus* A with *Ac. gramineus* (**Extended Data Figures 5**)." Change to "Chr1 of *Ac. calamus* A has good collinearity with Chr11 of *Ac. calamus* B (A1-1 and A1-3), except for A1-2 (**Extended Data Figures 7**), indicating either Chr1 of *Ac. calamus* A or Chr11 of *Ac. calamus* B retained the ancestral karyotype. We further compared Chr1 of *Ac. calamus* A with *Ac. gramineus* (**Extended Data Figures 5**)." Please see Page 12 Lines 305–308.

Comment 6.

Also, the newly presented data tended to be not fully explained and tended to repeat the mistakes that have already been pointed out elsewhere in the previous rounds of reviews. I should mention that insufficient labeling was among the points I have raised since the first revision.

Response: Please see the **Response to Comment 7**.

Comment 7.

Many figures have short, insufficient legends and very brief mentions in the main text, making it extremely difficult to understand the validity of data. Examples include, but may not be limited to: Extended Data Figure 2, Extended Data Figure 3, Extended Data Figure 5, Extended Data Figure 6, Supplementary Figure 19. For example (just as an example. It applies to the others too, and I expect the others to be addressed as well), the authors referenced Extended Data Figure 2 in the main text with the claim that putative centromeres may be detected in 16 and 6 chromosomes in *Ac. calamus* and *Ac. gramineus*, without indicating which of the illustrated peaks they think corresponds to the centromeres. The

authors should clearly indicate what data they are discussing, rather than letting readers chase the authors' logic without sufficient navigation. I should point out that I raised the same concern on another figure in earlier rounds of reviews, and I hope that this specific example of Extended Data Figure 2, as well as similar problems in the other figures, which may be listed above, will be fixed in the next version.

Response: Thanks for your advices. We have revised the legend and added explanation of all figures including Figures 1-5, Extended Data Figures 1-10 and all Supplementary Figures. We also revised and marked the figure of *K*-mer distribution, added the ploidy analysis results by Genomescope2 (please see **Supplementary Figures 3-4**). The figures mentioned have replaced in the text, for example, **Extended Data Figure 2**.

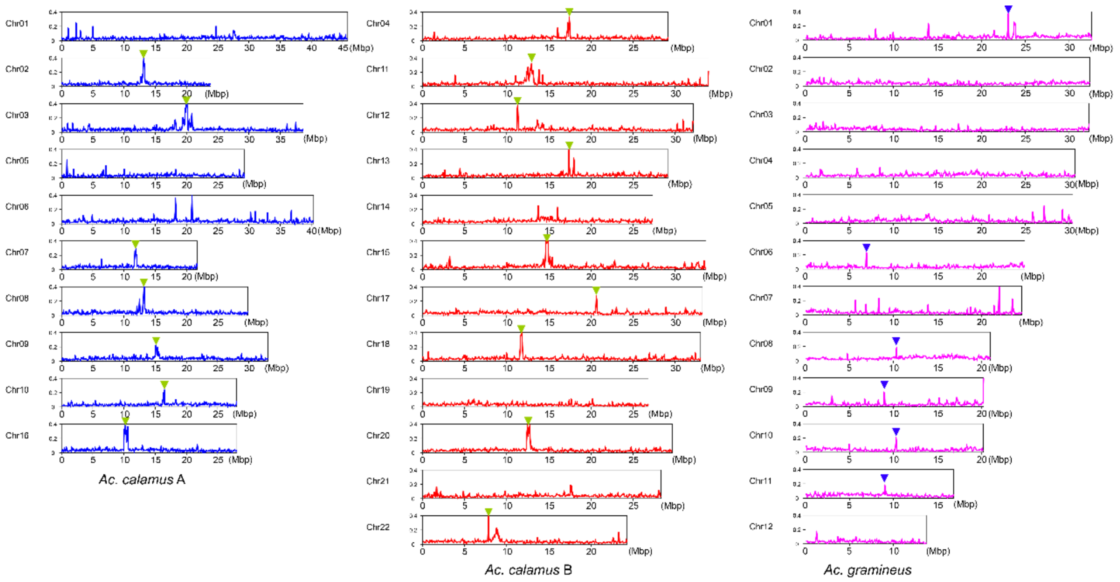

Extended Data Figure 2. Comparison of tandem repeat distribution between *Ac. calamus* A(a) and B (b), and *Ac. gramineus* (c). The 100kb windows was used to calculate the ratio of tandem repeat, the regions enrichment of tandem repeat might represent the centromere/telomeric. In *Ac. calamus*, 16 putative centromeres may be detected (chr2,3,7,8,9,10,16,4,11,12,13,15,17,18,20,22), in *Ac. gramineus,* 6 putative centromeres may be detected (chr1,6,8,9,10,11).

Extended Data Figure 3. **Concatenated and multi-species coalescent (MSC) phylogenetic tree based on nucleotides (a) and amino acids (b) of *Ac. calamus*, *Ac. gramineus* and other 16 species.** The branch length of the concatenated tree indicates the number of substitutions per site and the red numbers indicate the bootstrap. The blue numbers on the MSC tree indicate that q1, q2, and q3 refer to the quartet support for the main topology, the first alternative, and the second alternative.

Supplementary Figure 19. **The distribution of collinear block between *Ac. calamus* (A and B), *Ac. gramineus* and *A. trichopoda* syntenic depths**. The red histogram shows the main pattern of collinear gene ratio between the two genomes. The black histogram shows the no-collinear region and the not-main pattern of collinear gene ratio. JCVI with default parameters was used to make the graph.

Please see **Response to Comment 8** for the legend of Extended Data Figure 5 and Extended Data Figure 6**.**

We also have modified the following content:
"Based on these deductions, we thus reconstructed the MRCA karyotype for *Ac. gramineus* and *Ac. calamus* with ten chromosomes. We found that *Ac. calamus* B and *Ac. gramineus* experienced specific chromosome split events, which may explain why the chromosome number of *Ac. calamus* B and *Ac. gramineus* was 12." change to "Based on these deductions, we thus reconstructed the MRCA karyotype for *Ac. gramineus* and *Ac. calamus* with ten chromosomes (Extended Data Figure 8). We found that *Ac. calamus* B (B15 and B19 in Extended Data Figure 8) and *Ac. gramineus* (G1 and G2 in Extended Data Figure 8) experienced specific chromosome split events, which may explain why the chromosome number of *Ac. calamus* B and *Ac. gramineus* was 12." (Please see page 12, lines 313–318)

Comment 8.
Labels of many figures are not readable because of low image resolution. Examples include, but may not be limited to: Fig. 4d, Fig. 5, Extended Data Figure 2, Extended Data Figure 5, Supp Fig. 6, Extended Data Figure 2.
Response: Thank you for alerting us to this issue. We have met the requirement of 300 pixels for all figures, so we independently uploaded high-definition figures through the submission system. The font size of **"**Fig. 4d" and "Extended Data Figure 2" have been modified to increase clarity. We also have enlarged the labels in the image and updated the legends of "Fig. 5" for easy reading.

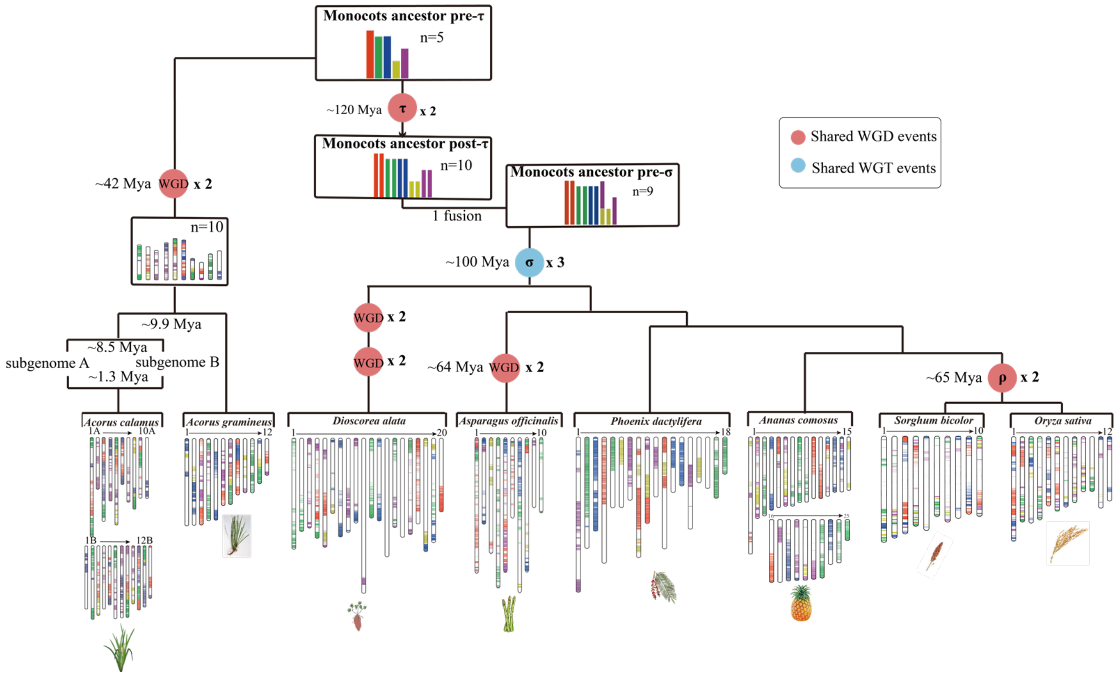

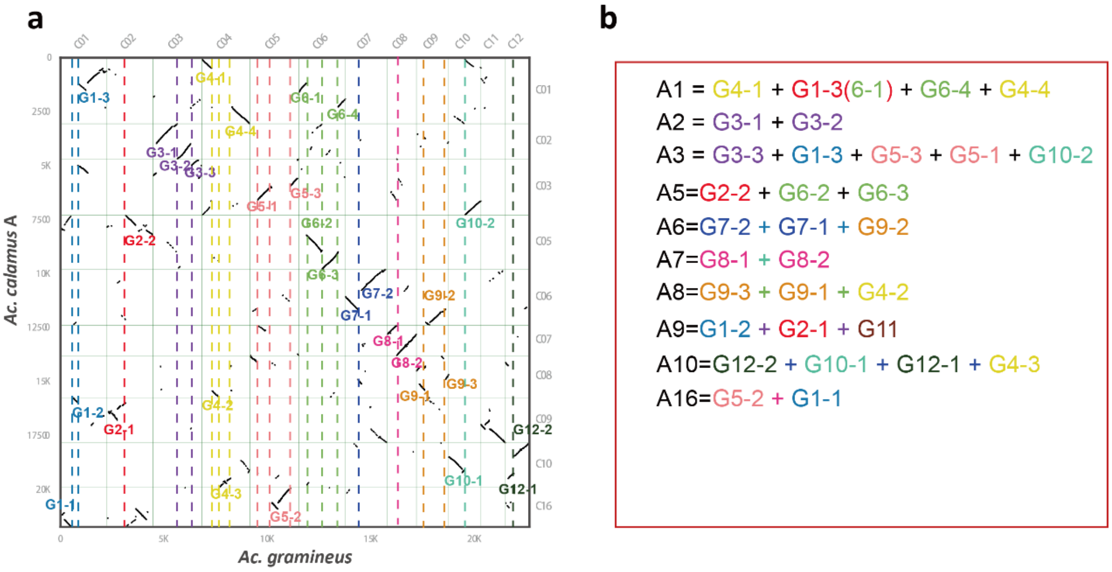

Extended Data Figure 5. Alignment of *Ac. calamus* A chromosomes with *Ac. gramineus* chromosomes. a Genome synteny visualization by dot plots. Different colors represent different chromosomes. The labels on the plot represent different chromosome segments. **b** The collinearity between *Ac. calamus* A and *Ac. gramineus* chromosomes. The colors are the same as in (a).

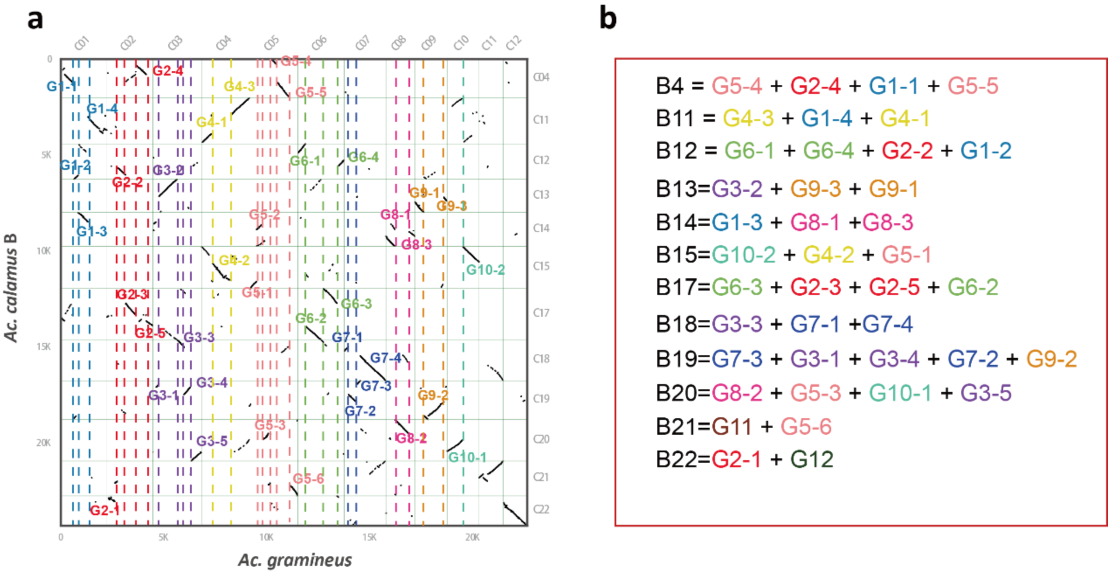

Extended Data Figure 6. Alignment of *Ac. calamus* B chromosomes with *Ac. gramineus* chromosomes. a Genome synteny visualization by dot plots. Different colors represent different chromosomes. The labels on the plot represent different chromosome segments. **b** The collinearity between *Ac. calamus* B and *Ac. gramineus* chromosomes. The colors are the same as in (a).

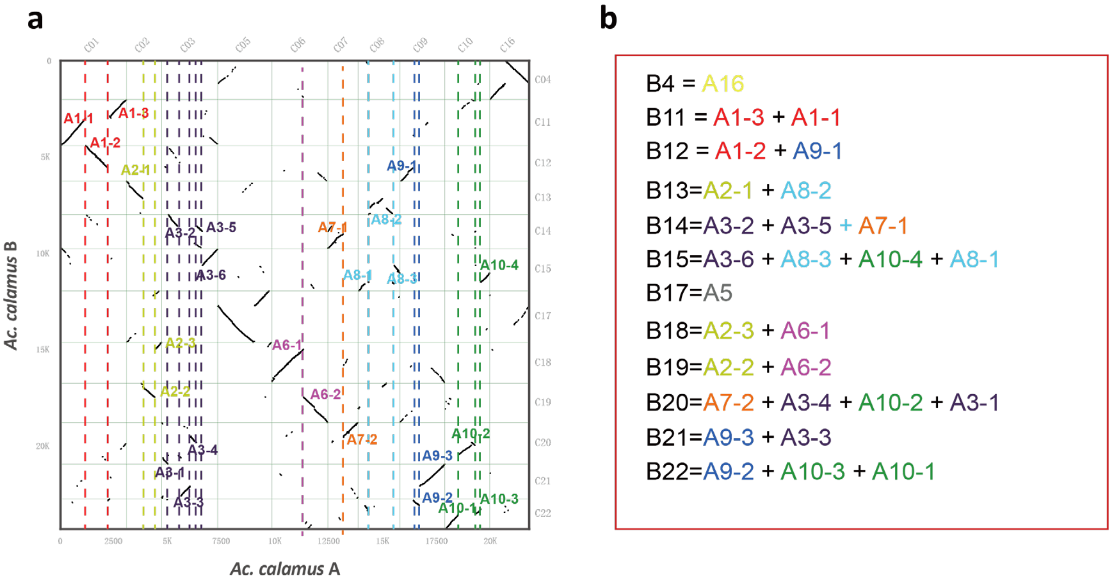

Extended Data Figure 7. Alignment of *Ac. calamus* A chromosomes with *Ac. calamus* B chromosomes. a Genome synteny visualization by dot plots. Different colors represent different chromosomes. The labels on the plot represent different chromosome segments. **b** The collinearity between *Ac. calamus* A and *Ac. calamus* B chromosomes. The colors are the same as in (a).

Comment 9.

All Figs should be self-contained, but labels and legends are missing in many figures. Examples include, but may not be limited to: X tick labels in Fig. 4c, colorbar in Fig. 4d, colors in Extended Data Figure 6, Y axis label of Extended Data Figure 7, colorbars in Extended Data Figure 8, colorbars in Extended Data Figure 10, support values and bars in Extended Data Figure 3, notations in Extended Data Figure 5d-f, dashed lines in Extended Data Figure 4, colorbar in Supplementary Figure 22, colorbar in Supplementary Figure 24.

Response: Thanks for your professional comments. We have checked all figures and ensured the labels and legends are correct. We have modified the coordinate axis name of "Fig. 4c", and added the color bar name of "Fig. 4d". We have enlarged the corresponding labels in the image of "Extended Data Figure 6" for easy viewing and updated the legend. Please see **Extended Data Figure 8**.

Extended Data Figure 8. The ancestral chromosomes reconstruction of the most recent common ancestor (MRCA) for *Acorales*. The first line represents the presumed ancestral karyotype pattern of *Acorales*. Lines 2 to 4 represent the chromosome karyotype of *Ac. calamus* A, *Ac. calamus* B and *Ac. gramineus*, respectively, with each small panel representing the chromosome formation process. The composition of ancestral chromosomes in modern plant genomes is shown below, with different colors representing different ancestral chromosomes.

We included the description of colorbar.

Extended Data Figure 9. Expression profiles of ABCDE MADS-box genes in *Acorus* floral organs. a. Expression profiles of ABCDE MADS-box genes in *Ac. gramineus* floral organs. b. Expression profiles of ABCDE MADS-box genes in *Ac. calamus* floral organs. The gene expression in both *Acorus* transcriptomes is represented by a color gradient from red to green. A heat map was generated to describe the expression level by FPKM of the genes among various floral organs.

The Extended Data Figure 9 was moved to Supplementary Figure 28.

We included the description of colorbar.

Extended Data Figure 10. Canonical biosynthetical pathway of strigolactone and the expression patterns of orthologous genes involved in each biosynthetic step of strigolactone in both *Acorus* species. The gene expression in both *Acorus* transcriptomes is represented by a color gradient from red to green. A heat map was generated to describe the expression level by FPKM of the genes among various tissues. **a** Canonical biosynthetical pathway of strigolactone and the genes involved in each biosynthetic step of strigolactone.

D27: DWARF27, -carotene isomerase. CCD7: Carotenoid Cleavage Dioxygenases 7. CCD8: Carotenoid Cleavage Dioxygenases 8. MAX1: More Axillary Growth1, CYP711A. **b** The orthologous gene IDs with the expression level by FPKM in various tissues of Ac. gramineus. **c** The orthologous gene IDs with the expression level by FPKM in various tissues in A and B genomes of *Ac. calamus*. Fl: flowers; Le: leaves; St: stems; Ro: roots. Bar in the photos of *Ac. gramineus* and *Ac. calamus* are 20 cm.

The Figure legend of "Extended Data Figure 3" has been revised.

In order to solve the problem of low resolution of the original Extended Data Figure 5d-f (**Comment 8**), we split the original figure and explained the symbols in the figure in detail in the note (see Respond to Comment 8 for details):
The legend of **Extended Data Figure 4** has been modified and the description of dotted lines has been added as follow.
Extended Data Figure 4. ***Ks* distribution among *Ac. calamus A*, *Ac. calamus B*, *Ac. gramineus* and *Sp. polyrhiza*.** The enlarged part was the Ks distribution of *Ac. calamus A*, *Ac. calamus B* and *Ac. gramineus*. The dashed lines showed the peaks of *Ks* distribution.

The color bar names have been added in **Supplementary Figure 22** and **Supplementary Figure 24**.

Comment 10.
Abbreviations are not defined in many figures. Examples include, but may not be limited to: X tick labels of Extended Data Figure 10.
Response: Thank you for your comments. We have defined Abbreviations of all figures, including **Extended Data Figure 10** (Please see **Supplementary Table 32).**

Comment 11.
Fig. 2. Green and red in small pie charts are not explained. I assume they represent expanded/contracted gene families, but figure legends should be self-contained without ambiguity. I noticed that the authors correctly explained it in the response letter to Reviewer 2, but did not revise the legend so.
Response: The figure legend has been revised as follow:
Figure 2. **Phylogenetic tree showing divergence times and the evolution of gene family size in 19 species**. The green and red numbers are the numbers of expanded and contracted gene families, respectively. The blue portions of the pie charts represent the gene families whose copy numbers are constant. The orange portions of the pie charts represent the

proportion of the 11,421 gene families found in the most recent common ancestor (MRCA) that expanded or contracted during recent differentiation. Star, showing the time and position of genome polyploidy event, the blue, yellow, red and green stars represent *N. tetragona*, core eudicot, *Acorus* and other monocots, respectively. For each branch, the pie chart shows the gene family number of contracted (green), expanded (red) and stable (blue).

Comment 12.

Extended Data Figure 3: Some species have dashed lines, but others do not. Please explain what it indicates. Also, please define the unit of scale bars. Please define the values around branches. Is there any reason to show branch lengths in coalescence-based trees but not in concatenation-based trees?

Response: The dashed lines have been removed, a scale bar has been added, and the legend has been updated. The revised figure can be found in "CP.combine.tre.V2.pdf". Sorry, the left side should be concatenation-based trees and the right side should be coalescence-based trees. The concatenation-based tree was a phylogram tree, it takes into the number of substitutions per site, so each branch had a different length. But coalescence-based tree was a cladogram, it just integrated the topologies, so the branch lengths was meaningless.

Comment 13.

Extended Data Figure 7: What does the dashed line indicate?

Response: Thank you very much. Extend Data figure 7 was not helpful for understanding our analysis results and could lead to ambiguous interpretations, so we have removed it.

Comment 14.

Extended Data Figure 7: I failed to understand what this figure shows. Legends say the data are from pairwise comparisons, but the labels in the figure indicate only one species, not pairs. Could you clarify more?

Response: Thank you very much. Extend Data figure 7 was not helpful for understanding our analysis results and could lead to ambiguous interpretations, so we have removed it.

Comment 15.

Supplementary Figure 1: Please add a scale bar if available.

Response: We have added a scale in **Supplementary Figure 1**.

Comment 16.

L73 "mosaic genomic features, important for...": could be "likely important for", as provided evidence is only gene expression, which may or may not correlate with phenotypes.

Response: Thank you for your kind suggestion. We changed the sentence to "Our analyses show that the ancestors of monocots exhibited mosaic genomic features, likely important for flower development and cotyledon evolution, vascular cambia, secondary xylem development, and adaptation to wetland environments, providing fundamental insights into the origin, evolution, and diversification of monocots." accordingly. Please see page 3, lines 72−76.

Comment 17.

L386 "most genes having evidence for purifying selection were located on subgenome B ...(Figure 4c).": From what I can see in Fig. 4c, there is very little difference between subgenomes A and B, and most genes in both subgenomes seem to be under purifying selection, given the Ka/Ks distribution being mostly below 1.0. The authors should elaborate on the logic behind their claim, preferably with appropriate statistical testing.

Response: Thank you for your suggestion. We realize that the expression in that sentence was not very accurate. Based on the combination of Fig. 4c and Fig. 4d, the high-expression genes were subjected to stronger purification selection, rather than a particular subgenome undergoing high purification selection. For example, in Class I genes, when expressed at high levels in subgenome A, the ka/ks value of I_A becomes closer to 0, while in Class II genes, when expressed at high levels in subgenome B, the *k*a/*k*s value of II_B becomes closer to 0.

A similar analysis is presented in this article (Xu, P. *et al.* The allotetraploid origin and asymmetrical genome evolution of the common carp *Cyprinus carpio*. *Nat Commun* **10**, 4625 (2019)), and their results are consistent with ours. Therefore, we have modified this sentence to "the dominantly transcribed homoeologous copies, regardless of their subgenome location, were more likely experiencing stronger purifying selection than their homoeologs." Please see page 15 lines 377−379

Comment 18.

L752 "the file of main result was k13_q50_f2.bin.enrich; The file with concrete results is k13_q50_f2.bin.enrich.": Are you providing two sets of results for different analyses? I was confused by how "main result" and "concrete results" could be different, and further by that the same file name was referred to in both cases.

Response: The main result should be the file "k13_q50_f2.bin.group", and more specifically, the result is "k13_q50_f2.bin.enrich". Please see page 28 line 741.

Comment 19

L767 "the most poorly aligned sequences": Could you provide a quantitative threshold?

Response: Thank you very much. The use of non-strict parameters in the blast before

orthoMCL would not affect the clustering results, as orthoMCL algorithm can handle this automatically.

In Chen, F. et al.'s article (Chen, F. *et al*. OrthoMCL-DB: querying a comprehensive multi-species collection of ortholog groups. *Nucleic Acids Res*. **34** (Database issue): D363–D368 (2006)), it is mentioned that "Although this might be expected to include many false positives, rules applied during group identification (reciprocal best/better hits, Markov clustering) eliminate most poorly alignable sequences. Considering the entire clustered dataset, 79% of all pairs within OrthoMCL groups were recognized in the initial BLAST search, and display an average E-value = 1 × 10−114, average percent identity = 53% and average percent coverage = 85%." The original article did not provide a detailed definition for "poorly alignable sequences". Sorry, we can't define "poorly alignable sequences".

Comment 20

L782 "T=Ks / 2r.": r should be defined.

Response: Thanks for your comment. r means the Ks rate, which was 5.26e-9 per site per year for the *Acorus* branch. Please see page 29 lines 769−770.

Comment 21.

L793 "GTR + gamma model": The gamma model should be defined in terms of the number of site-wise rate categories.

Response: Four rate categories are used. According to the tutorial of MrBayes, four rate categories are sufficient in most cases.

Comment 22

L821 P < 0.01 and FDR < 0.05": I failed to understand why significance thresholds are set for both unadjusted P and FDR. Could you clarify the logic behind the design of the analysis?

Response: Thanks. We use both unadjusted P and FDR to reduce false positive biases in identifying homologous genes with biased expression. This method for identifying biased expression of homologous genes is from the published article "Sequencing of allotetraploid cotton (*Gossypium hirsutum* L. acc. TM-1) provides a resource for fiber improvement".

The method described in the article is as follows:

"Homoeologous bias expression was detected in the entire 35 tissue data set through pairwise t-tests (significance was set at P < 0.01, FDR < 0.05 and at least twofold differences in average expression levels)." Please see page 30 lines 807−809.

Comment 23

L823: dot-"p"lot?

Response: We have changed it to “dot-plot”. Please see page 31 line 825.

Comment 24

The authors added RNA-seq data for floral organs in the two Acorus species without sample information (e.g., developmental stages). Appropriate descriptions, as well as photographs of flower anatomy (to show how the authors dissected samples), should be provided. This seems to be the case in other samples as well. For example, How Flo-1 to Flo-3 are staged does not seem to be explained at all.

Response: Thank you for your kind suggestion. We have added description and photographs of flower anatomy as well as the other samples used for RNA-seq data. Please see **Extended Figure 10**, and **Supplementary Figure 29** (The Extended Data Figure 8 was transferred to Supplementary Figure 28).

Extended Data Figure 10. Canonical biosynthetical pathway of strigolactone and the expression patterns of orthologous genes involved in each biosynthetic step of strigolactone in both *Acorus* species. The gene expression in both *Acorus* transcriptomes is represented by a color gradient from red to green. A heat map was generated to describe the expression level by FPKM of the genes among various tissues. a Canonical biosynthetical pathway of strigolactone and the genes involved in each biosynthetic step of strigolactone. D27: DWARF27, -carotene isomerase. CCD7: Carotenoid Cleavage Dioxygenases 7. CCD8: Carotenoid Cleavage Dioxygenases 8. MAX1: More Axillary Growth1, CYP711A. b The orthologous gene IDs with the expression level by FPKM in various tissues of *Ac. gramineus*. c The orthologous gene IDs with the expression level by FPKM in various tissues in A and B genomes of *Ac. calamus*. Fl: flowers; Le: leaves; St: stems; Ro: roots. Bar in the photos of *Ac. gramineus* and *Ac. calamus* are 20cm.

Supplementary Figure 29. Expression profile of MADS-box genes in reproductive organs and vegetative organs. a Expression profile of *Ac. gramineus* MADS Type II genes (Flo, flower; Inf, inflorescence; See, seed; Fri, fruit; Lea, leaf; Roo, root; Ste, stem). Bar, 2mm: flower, seed and fruit; 20 cm: inflorescence, leaf, root and stem. **b** Expression profile of *Ac. calamus* MADS Type II genes (Flo-1, flowering 1 stage (the bud, diameter 1.5 mm); Flo-2, flowering 2 stage (the flower is opening, diameter 2.5 mm); Flo-3, flowering 3 stage (the flower fully opened, diameter 4 mm); Bra, bract; Roo, root; Lea, leaf; Ste, stem). Bar, 1cm: flowering 1-3 stages, bract, root, stem; 10cm: leaf.

Comments 25

To be clear, many of the above points are explained with a few examples among many similar

problems. I expect that all similar cases for individual concerns will be identified throughout the manuscript by the authors, and corrections will be made even though some of them are not individually mentioned in the examples. It is important for the authors to thoroughly address all of these issues in order to improve the readability and clarity of the manuscript.

Response: We deeply appreciated you providing your concerns for our manuscript. We have rechecked the manuscript thoroughly and did our best to address all of your concerns. If there are some points need to be improved, please let us know.

Reviewers' Comments:

Reviewer #2:
Remarks to the Author:
The authors have addressed my original points in this revised manuscript, and I have no further comments and suggestions.

Reviewer #3:
Remarks to the Author:
I would like to thank you for taking the time to address my previous comments, and I am pleased to see that the manuscript has improved in many aspects. I have a few minor concerns that I would like to bring to your attention. However, I have highlighted everything that I can, and I leave the remaining issues to the discretion of the editor.

Firstly, regarding Comment 2, while it appears that the labeling issue has been corrected in many figures, I noticed that the author's response seems to be out of place, addressing something other than my original comment on the Figure labeling.

Secondly, I am still confused by the distinction between "main result" and "concrete results", even after reading the updated manuscript where the file name was corrected, but no further clarification was provided. It would be helpful if you could address this issue in the manuscript.

Thirdly, I understand that the authors were unable to define the "most poorly aligned sequences", but I would suggest that the wording in the manuscript be revised to avoid propagating any inaccuracies.

Fourthly, thank you for your response to Comment 21. It would be useful if you could include an explanation of the use of four gamma categories in the manuscript.

Finally, while I appreciate that the two-step significance thresholding may have been used in previous literature, I would like to note that it is not a standard approach. However, I am satisfied that the data processing is now clear to readers, and I do not request any further changes.

Response to Reviewer's Comments

Reviewer #2 (Remarks to the Author):

The authors have addressed my original points in this revised manuscript, and I have no further comments and suggestions.
Response: Thank you very much for recognizing of our efforts.

Reviewer #3 (Remarks to the Author):

I would like to thank you for taking the time to address my previous comments, and I am pleased to see that the manuscript has improved in many aspects. I have a few minor concerns that I would like to bring to your attention. However, I have highlighted everything that I can, and I leave the remaining issues to the discretion of the editor.
Response: We are grateful for the positive assessment of our efforts by the reviewer. Please find our point to point responses to the comments as follows.

Comment 1
Firstly, regarding Comment 2, while it appears that the labeling issue has been corrected in many figures, I noticed that the author's response seems to be out of place, addressing something other than my original comment on the Figure labeling.
Response: We agree with the reviewer's opinion in the original Comment 22 claiming that "the statement 'it's an allotetraploid due to different numbers and size of chromosomes' is not necessarily true", so that we removed such statement, provided new results based on genomescope2 and smudgeplot, and used both k-mer analysis and subphaser to distinguish the subgenomes *Ac. calamus* in our previous revision.

In the current revision, we have clarified how Hi-C signal hint at a homoeologous relationship between a pair of chromosomes (see page 6, lines 139–144, 147–148 in the main text). Also, we removed the title "Distinguishing allo-subgenomes by collinear Hi-C signal" in Methods and concatenated the detailed methods in the section "The Hi-C scaffolding" (see page 26, lines 687–692).

We have further changed the title of Figure 1, which now reads "Hi-C scaffolding of the allotetraploid *Ac. calamus* genome and subgenome reconstruction" (please see

page 47, Figure 1). Also, the title of Figure 1a now reads “Hi-C contact matrix of the 22 chromosomes in the *Ac. calamus* genome” (please see page 48, Figure 1a).

Comment 2

Secondly, I am still confused by the distinction between "main result" and "concrete results", even after reading the updated manuscript where the file name was corrected, but no further clarification was provided. It would be helpful if you could address this issue in the manuscript.

Response: Thank you so much for spotting the confusing words used in the Methods. To avoid further confusions, we have removed the sentences saying “main result” and “concrete results”, because they were named by the authors for internal communications. Instead, in the current revision, we referred the readers to Supplementary Figure 7c to see the homoeologous exchanges inferred by SubPhaser (Please see page 28, lines 744–749).

Besides, we can explain the two files here as responses to the remarks. What we called “main result” was the file “k13_q50_f2.bin.group”, which contained “grouped bins by potential exchanges based on enrichments”; and what we called “concrete results” was the file “k13_q50_f2.bin.enrich”, which contained “subgenome-specific enrichments by genome window(bin)”. Both files were part of the output of SubPhaser and were used, together with other output files, to generate Supplementary Figure 7.

Comment 3

Thirdly, I understand that the authors were unable to define the "most poorly aligned sequences", but I would suggest that the wording in the manuscript be revised to avoid propagating any inaccuracies.

Response: We modified the relevant sentence into “Because we aimed at identifying orthogroups across angiosperms and OrthoMCL[115] can deal with mis-specified homologous sequence pairs occasionally produced by BLASTP, we filtered the BLASTP results with an E-value threshold of 1×10^{-5}, a similarity threshold of 30%, and a coverage (alignment length divided by query sequence length) threshold of 50%” (Please see page 28, lines 758–764).

Comment 4

Fourthly, thank you for your response to Comment 21. It would be useful if you could include an explanation of the use of four gamma categories in the manuscript.

Response: We have added to following sentences to explain the usage of the discrete

gamma distribution for variable rates across sites:" A phylogenetic analysis of the data set was performed using MrBayes[122] under the GTR + GAMMA model with four categories (Ngammacat = 4) in the discrete Gamma model to take the heterogeneity of substitution rates among sites into consideration. It has been shown that as few as four rate categories in the discrete Gamma model are not only computationally practical but can also approximate the continuous Gamma distribution to model variable rates among sites[123]." (Please see pages 29–30, lines 789–794).

Comment 5

Finally, while I appreciate that the two-step significance thresholding may have been used in previous literature, I would like to note that it is not a standard approach. However, I am satisfied that the data processing is now clear to readers, and I do not request any further changes.

Response: We have added the relevant reference, Zhang et al., 2015 (Sequencing of allotetraploid cotton (*Gossypium hirsutum* L. acc. TM-1) provides a resource for fiber improvement) in the Methods (Please see page 31, lines 822–824).

Reviewers' Comments:

Reviewer #3:
Remarks to the Author:
I thank the authors for the further clarifications. The authors addressed many of my concerns, and I have no further requests.

Response to Reviewer's Comment

Reviewer #3 (Remarks to the Author):

I thank the authors for the further clarifications. The authors addressed many of my concerns, and I have no further requests.

Response: Thank you very much for recognizing of our efforts.
